# Transcriptome analysis of *Anastrepha fraterculus* sp. 1 males, females, and embryos: insights into development, courtship, and reproduction

**DOI:** 10.1186/s12863-020-00943-2

**Published:** 2020-12-18

**Authors:** Alejandra Carla Scannapieco, Claudia Alejandra Conte, Máximo Rivarola, Juan Pedro Wulff, Irina Muntaabski, Andrés Ribone, Fabián Milla, Jorge Luis Cladera, Silvia Beatriz Lanzavecchia

**Affiliations:** 1 Instituto de Genética “E. A. Favret” (IGEAF) gv Instituto de Agrobiotecnología y Biología Molecular (IABIMO), Instituo Nacional de Tecnología Agropecuaria (INTA) - Consejo de Investigaciones Científicas y Técnicas (CONICET), Hurlingham, Buenos Aires, Argentina; 2 Instituto de Biotecnología, IABIMO, INTA – CONICET, Hurlingham, Buenos Aires, Argentina

**Keywords:** Fruit fly, RNA-Seq analysis, Transcript annotation, Differential gene expression, Microsatellite markers

## Abstract

**Background:**

*Anastrepha fraterculus* sp. 1 is considered a quarantine pest in several American countries. Since chemical control applied in an integrated pest management program is the only strategy utilized against this pest, the development of pesticide-free methods, such as the Sterile Insect Technique, is being considered. The search for genes involved in sex-determination and differentiation, and in metabolic pathways associated with communication and mating behaviour, contributes with key information to the development of genetic control strategies. The aims of this work were to perform a comprehensive analysis of *A. fraterculus* sp. 1 transcriptome and to obtain an initial evaluation of genes associated with main metabolic pathways by the expression analysis of specific transcripts identified in embryos and adults.

**Results:**

Sexually mature adults of both sexes and 72 h embryos were considered for transcriptome analysis. The de novo transcriptome assembly was fairly complete (62.9% complete BUSCO orthologs detected) with a total of 86,925 transcripts assembled and 28,756 GO annotated sequences. Paired-comparisons between libraries showed 319 transcripts differently expressed between embryos and females, 1242 between embryos and males, and 464 between sexes. Using this information and genes searches based on published studies from other tephritid species, we evaluated a set of transcripts involved in development, courtship and metabolic pathways. The qPCR analysis evidenced that the early genes *serendipity alpha* and *transformer-2* displayed similar expression levels in the analyzed stages, while *heat shock protein 27* is over-expressed in embryos and females in comparison to males. The expression of genes associated with courtship (*takeout*-like*, odorant-binding protein 50a1*) differed between males and females, independently of their reproductive status (virgin vs mated individuals). Genes associated with metabolic pathways (*maltase 2-*like*, androgen-induced gene 1*) showed differential expression between embryos and adults. Furthermore, 14,262 microsatellite motifs were identified, with 11,208 transcripts containing at least one simple sequence repeat, including 48% of di/trinucleotide motifs.

**Conclusion:**

Our results significantly expand the available gene space of *A. fraterculus* sp. 1, contributing with a fairly complete transcript database of embryos and adults. The expression analysis of the selected candidate genes, along with a set of microsatellite markers, provides a valuable resource for further genetic characterization of *A. fraterculus* sp. 1 and supports the development of specific genetic control strategies.

**Supplementary Information:**

The online version contains supplementary material available at 10.1186/s12863-020-00943-2.

## Background

The South American fruit fly, *Anastrepha fraterculus* Wiedemann (Diptera: Tephritidae), is considered one of the most economically important pest species in several American countries. This species has broad geographical distribution and a wide host range, attacking at least 110 host plant species including major fruit crops [1–5]. Its presence limits international trade because of quarantine regulations to avoid cross-border introductions [6–8]. Although *A. fraterculus* is currently considered a complex of cryptic species [9–12], only one biological entity, named *A. fraterculus* sp. 1 [10] or Brazilian 1 morphotype [11], has been detected in Argentina and southern Brazil.

The most effective control measure against tephritid pests is the Sterile Insect Technique (SIT), in which flies are mass-reared, sterilized by radiation, and released into the wild where they mate with fertile individuals, subsequently laying unfertilized eggs, thereby reducing the pest population size [13, 14]. SIT has been successfully used in eradication programs against the Mediterranean fruit fly *Ceratitis capitata* [15], *Bactrocera dorsalis* and other *Bactrocera* species [16]. Also, it is currently being applied against *Anastrepha ludens*, and *A. obligua* [17–19]. However, only chemical approaches have been used to control *A. fraterculus* populations in the field to date [20].

The implementation of efficient procedures of sexing and sterilization during mass rearing is of importance for an effective SIT application. In particular, the physical sexing mechanisms for male and female separation (genetic sexing strain - GSS) are necessary to produce and release only males and thus avoid the costs of female rearing, mating competition, and oviposition damage to host plants, difficulties observed when bi-sexual strains were applied by SIT [19, 21, 22]. The GSS has been developed using classical approaches for several species of fruit flies, such as *A. ludens* [19, 23], *B. dorsalis* [24], *C. capitata* [25, 26], and *Zeugodacus cucurbitae* [27]. In *A. fraterculus*, six pupal colour-based GSS strains have been developed and are currently under evaluation at the IAEA Insect Pest Control Laboratory [28].

In addition to these developments, genes involved in the sex determination pathways have been described for *A. fraterculus*. For example, those belonging to the primary sex-determining cascade, such as *transformer* [29], *transformer-2* [30] and *doublesex* [31] have been studied in *A. obliqua* and *A. fraterculus,* providing valuable information to improve the existing *A. fraterculus* GSS strains generated by the traditional approach. Furthermore, this knowledge lays the molecular basis to develop GSS in this species by insect genetic engineering systems such as transgenesis and CRISPR Cas9 techniques, successfully utilized in other Tephritid species ([32, 33] and references therein).

The study of the molecular mechanisms underlying the physiological processes involved in communication, courtship, and reproduction is also essential for the development of SIT approaches, including the efficient mass rearing of the insect pest species, and the competitiveness of sterile individuals in the field. For example, mating can induce profound physiological changes and behavioural switches in females, including changes in oviposition patterns, mating refractoriness, and longevity [34–36] which have practical consequences for SIT success. Furthermore, mating success is strongly associated with communication among individuals and the detection of volatile phytochemicals [37, 38]. These volatiles affect the behaviour and physiology of phytophagous insect species and are key components of the mating competitiveness of *A. fraterculus* males in the field [39–41]. Recent studies have utilized a transcriptome approach to identify and differentiate physiological aspects of *Anastrepha* species [42] and deepen the knowledge of the molecular mechanisms involved in olfaction [43–45] and cell processes [46, 47].

In the present study, we performed a full transcriptome analysis of *A. fraterculus* sp. 1 in 72 h embryos and adults to contribute to a fairly complete sequence database of this species. Using our de novo GO annotation, specific gene searches and a strict filtering of transcripts by their expression profiles, we selected putative transcripts involved in development, courtship and metabolic pathways and analyzed their expression by qPCR in embryos, as well as virgin and mated adults. Furthermore, we identified microsatellite motifs to augment the set of available molecular markers of this species to monitor the pest in the field.

## Results

### Sequencing and annotation

The RNA-Seq analysis of the three libraries (72 h embryos, adult virgin females and adult virgin males) from *A. fraterculus* sp. 1 yielded an average of 5,535,000 reads per library, with a mean read length of 151 base pairs (bp) and a low percentage of discarded sequences (< 0.14%; Table 1). A total of 86,925 assembled sequences showed an average length of 682 bp (ranging between 200 and 12,700 bp). A BLASTX search (Expected Value cut off: 1.0x10E-10; Number Blast Hits retrieved: 10; Default scoring matrix: Blossum62) against the complete NR database of NCBI was performed to characterize all the assembled transcripts. A total of 52,655 transcripts had at least one hit (Table 1). Overall, we obtained a total of 471,646 BLAST hits against our *A. fraterculus* sp. 1 transcriptome and evidenced that the most highly represented species (showing the highest number of hits) was *C. capitata* (18.6% of BLAST hits) followed by *Z. cucurbitae* (previously known as *Bactrocera cucurbitae*) (16.7%) and by *B. dorsalis* (14.1%). Conversely, when only the top BLAST hit was analyzed (a total of 52,665 hits), *B. dorsalis* appeared in the first position with 18,642 hits, followed by *Z. cucurbitae* (16,823 hits) and *C. capitata* (15,333 hits).

**Table 1:** Summary of the *A. fraterculus* sp. 1 transcriptome

Total number of reads (72 h embryos/ Adult females/ Adult males)	5,519,495/ 4,765,195/ 6,322,876
Number of discarded reads (72 h embryos/ Adult females/ Adult males)	6640/ 6653/ 6356
Mean read length (bp)	151
Smallest transcript length (bp)	201
Number of largest transcript length (bp)	12,701
Number of overall average length (bp)	682.8
Median transcript length (bp)	410
N50 (bp)	1020
Total assembled sequences	86,925
Number of loci	57,000
Number of GO annotated sequences	28,756
Number of BLAST annotated sequences	52,655
GC% (72 h embryos/ Adult females/ Adult males)	44/ 44/ 42
Complete BUSCOs^a^	1762 (62.9%)
Complete and single-copy BUSCOs ^a^	827 (29.5)
Complete and duplicated BUSCOs ^a^	935 (33.4%)
Fragmented BUSCOs ^a^	484 (17.3%)
Missing BUSCOs^a^	553 (19.8%)

^a^BUSCO completeness assessment results for *A. fraterculus* transcriptome. Results are based on a complete set out of 2799 single-copy orthologs. *bp* base pairs

**Fig. 1:**
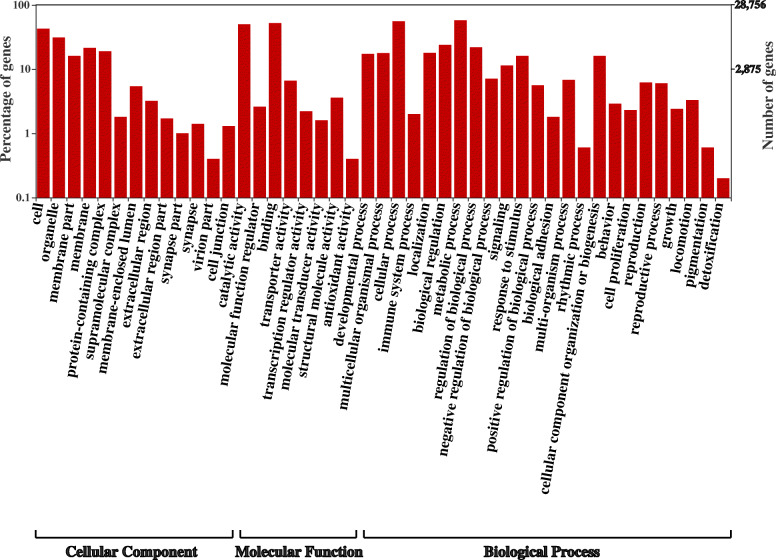
Gene Ontology (GO) assignment of *A. fraterculus* sp. 1 transcripts. The results are summarized in the three main categories “cellular component”, “molecular function” and “biological process”. GO was assigned to 28,756 transcripts in total. The percentage (left Y-axis) and total number (right Y-axis) of transcripts in each category (the second GO level) are shown. Y-axes are in log (10) scale. WEGO was used to produce the graph

Gene Ontology assignment yielded 28,756 GO annotated transcripts (Fig. 1). The distribution of *A. fraterculus*sp.1 annotated sequences in the three main categories showed 23,864 sequences within Molecular Function (GO: 0003674), 23,501 sequences in Biological Process (GO: 0008150), and 15,980 transcripts in Cellular Component (GO: 0005575). The major GO terms within the Molecular Function category were “binding” (GO: 0005488; 14,921 transcripts; 51.9%) and “catalytic activity” (GO: 0003824; 14,353 transcripts; 49.9%). For the Biological Process category, “cellular process” (GO: 0009987; 15,910 transcripts; 55.3%) and “metabolic process” (GO: 0008152; 16,513 transcripts; 57.4%) were the most highly represented subcategories. In the case of the Cellular Component category, “cell” (GO: 0005623) was the most highly represented subcategory with 12,284 transcripts (42.7%) (Fig. 1). In addition, the most highly represented subcategories (the third GO level) were obtained (Additional File 1) including main terms involved in biological processes, development, behaviour, and reproduction (Additional File 2).

### Ortholog prediction and quality of *A. fraterculus* transcriptome

The BUSCO analysis was performed to evaluate the completeness of our transcriptome. We recovered 62.9% of the complete orthologs from the BUSCO Diptera dataset with approximately 20% of these orthologs missing in our assembly (Table 1). Some of the missing orthologs are probably an indication of an incomplete transcriptome assembly, while others are possibly due to extremely low levels of expression in our experimental design. To further inspect and compare our assembly (INTA), we downloaded and ran a de novo assembly adding an RNA-Seq dataset from the USDA-ARS (extracted from the same *A. fraterculus* morphotype and with a similar experimental design; NCBI BioBroject: PRJNA338343). The USDA-INTA de novo assembly performed similarly in terms of BUSCO ortholog detection (70% recovered), but poorly in terms of the new assembled transcripts, retrieving at least one BLASTX hit per sequence. Only 47.2% of USDA-INTA transcripts gave BLASTX hits, while 60.7% of INTA transcripts gave at least one hit. Furthermore, GO annotations from the BLASTX hits of USDA-INTA transcripts resulted in only 1.7% of transcripts with at least one GO annotation (Blast2GO), when compared to 55% from INTA assembled transcripts. According with these comparative results, we continued the data analysis using the INTA transcriptome assembly.

In addition to the BUSCO analysis, which uses nucleotide transcriptome data, a deeper evaluation into closely related species proteomes was performed. Specifically, all open reading frames (ORFs) were predicted regardless of BLASTX hits to NR DB. An in silico analysis using Transdecoder software resulted in the detection of 38,933 putative proteins with at least 100 amino acids. We identified ortholog groups by inter-specific pairwise comparisons (OrthoMCL) considering all predicted proteins of three closely related species [48, 49] with available and fully annotated genomes (RefSeq DB NCBI) and 18S rRNA complete gene sequence (BLASTN search; > 80% query coverage and the highest percentage of identity): *B. oleae* [PRJNA 293367/288990], *Rhagoletis zephyria* [PRJNA331175/321204], and *C. capitata* [PRJNA201381] (Additional File 3). The number of predicted orthologs in the 2-way paired analysis by OrthoMCL showed a similar number of ortholog proteins present in each comparison (Table 2). In addition, we identified the number of orthologs present in two, three, and four proteomes of closely related species, and found a similar trend of orthologs present in each comparison (Additional File 4). These results suggest that the reconstruction of the obtained transcriptome is fairly complete in comparison to closely related species with assembled genomes. Furthermore, BUSCO analysis showed 33.4% of complete and duplicated transcripts (Table 1), indicating that our transcriptome data are somewhat redundant.

**Table 2:** Ortholog prediction analysis for the *A. fraterculus* sp. 1 transcriptome compared to three closely related species (*B. oleae* and *R. zephyria* and *C. capitata*) (Additional File 3)*.* The table shows the number of sequences (predicted proteins) from each species with orthologs in every other proteome

		… with orthologs in …			
		*A. fraterculus*	*B. oleae*	*R. zephyria*	*C. capitata*
Number of proteins from …	*A. fraterculus*		16,420	16,931	16,508
	*B. oleae*	12,627		15,444	16,047
	*R. zephyria*	15,289	19,023		19,664
	*C. capitata*	15,205	20,057	19,413	

### Differential expression analysis between libraries

**Fig. 2:**
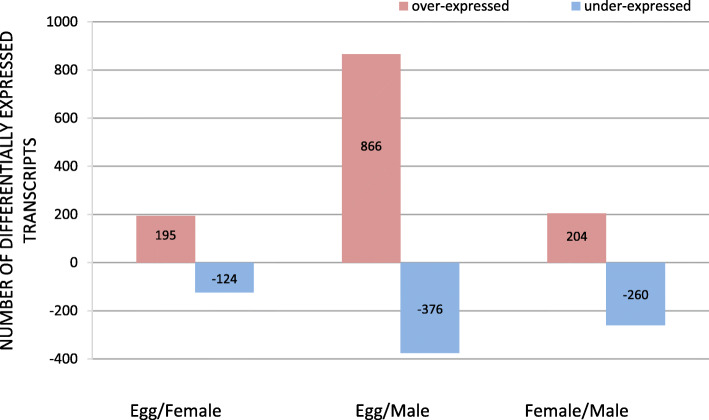
Descriptive analysis of differentially expressed transcripts in paired-comparisons between libraries. Values inside each bar indicate the number of differentially expressed transcripts (Y-axis) for each comparison (72 h embryo/ female, 72 h embryos/ male, female/ male). **72 h embryos/ females:** the number of transcripts over or under expressed in 72 h embryos compared to females. **72 h embryos/ males:** the number of transcripts over or under expressed in 72 h embryos compared to males. **Females/ males:** the number of transcripts over or under expressed in females compared to males. Threshold criteria: from the transcriptome assembly only transcripts with > 10 cpm were considered; FC > 10 over-represented transcripts; FC < 0.1 under-represented transcripts

Paired-comparisons between libraries were performed by DE analysis (EdgeR) considering only transcripts with more than 10 counts per million (cpm > 10) in at least two of the three samples. The Biological Coefficient of Variation (bcv) was set at 0.4, a value recommended by EdgeR [50] for comparing RNA samples of different conditions without replicates. We obtained 7188 differentially expressed transcripts in the 72 h embryo/ female comparison, 15,752 transcripts in 72 h embryo/ male comparison, and 7453 sequences between adults (female/ male comparison) (Additional File 5). Based on this database of differentially expressed transcripts, a subsequent manual filtering (threshold criterion of 10 fold-change [FC > 10 for over-expression] and 0.1 fold-change [FC < 0.1 for under-expression]) was performed. Results showed 319 differentially expressed transcripts in the 72 h embryo/ female comparison, 1242 transcripts in the 72 h embryo/male comparison, and 464 transcripts between females and males (Fig. 2; Additional File 6 A and B).

### Selection of transcripts for qPCR assay

Based on the information of the differentially expressed transcripts among libraries (Fig. 2; Additional File 6 A and B) and their predicted functions (GO annotation or the best BLAST hits), a selection was performed in order to identify those transcripts potentially associated to development, reproduction, courtship and communication in the *A. fraterculus* sp. 1 transcriptome. We detected transcripts annotated as heat shock proteins (*hsp23, hsp27* and *hsp70*), odorant-binding proteins (*obp1, obp19 and obp99*), and transcripts associated to female and male physiology (Additional File 7 A).

Our transcriptome was also screened for genes previously reported in the literature associated to early development and sex determination. This screening revealed the presence of several transcripts with a high similitude to early developmental genes, such as *serendipity alpha* and *slow-as-molasses* and to genes of the sex determination cascade (*transformer*, *transformer 2*, *doublesex*) (Additional File 7 B). In addition, we detected transcripts with high similarity to odorant-binding protein genes (*odorant-binding protein 49* and *odorant-binding protein 50a1)* previously described for *A. fraterculus* by Campanini et al. [45] (Additional File 7 B).

To perform a final selection of transcripts to be evaluated by qPCR, we filtered the transcripts from Additional File 7 (A and B) database by the unambiguous identification of the transcript (e.g. unique transcript sequence associated to a particular annotated gene without potential isoforms or with identifiable gene variants for the univocal analysis of its expression profile) (Table 3).

**Table 3:** Description, annotation and qPCR characteristics of selected transcripts

ID/ gene name^a^	GO term assigned	Accession no.^b^	Primer sequence 5′-3′	Product size (bp)	qPCR efficiency
***Development***					
Trinity DN12595_c0_g1_i2*/ serendipity alpha*	0016020/ 0007349/ 0005737	JQ599256	Fw: TGAGAGTTTGCGCACAGTGA Rv: CACTGCTGCACCTGAGTCAT	154	1.882
Trinity DN15480_c0_g1_i3*/ transformer-2*	–	KY204955	Fw: TGGAAATCGATGATCGCCGT Rv: GACGACGGGAGCGATAATCA	144	1.957
Trinity DN10186_c0_g1_i4*/ heat shock protein 27*	0005875/ 0008340/ 0042026/ 0042595	XM011197636	Fw: TTTCGGCTTTGGCTTGCATC Rv: TGCACACTTGGAAGCCATCT	200	1.929
***Courtship behavior/communication***					
Trinity DN11516_c0_g1_i3*/ takeout-*like	–	JQ048622	Fw: TTTACGGCGTTGGTAGTGCC Rv: CCGATCAACTCAGCTTTGGG	106	1.924
Trinity DN11027_c0_g1_i1*/ odorant-binding protein 50a1*	–	KU317977	Fw: GCTGCAGTCGTTGTCCATTG Rv: GTGTCGACATGCAACTCAGC	85	1.934
***Physiological pathways***					
Trinity DN15916_c0_g4_i3*/ androgen-induced gene 1 protein*	0012505/ 0016021	XM029042282	Fw: GCGTGTTGGATGCAGTGTTT Rv: CGTTTGGGATAGGCACGGAA	109	1.905
Trinity DN12527_c0_g1_i1*/ maltase 2-*like	–	XM011180327	Fw: CCAGCCCGAATACGGTACAA Rv: CGGTCGACGGACTTCTGAAA	138	1.907
***Reference genes***					
Trinity DN6544_c0_g1_i2*/ ribosomal protein L18*	0000022/ 0003735/ 0022625/ 0006412	NM139834	Fw: GAAAGGCTCCTGGTGTTCCA Rv: TTTGTAACCGCAGCTTGACC	104	1.963
Trinity DN12142_c0_g1_i2*/ elongation factor - 1α*	0008340/ 0003746/ 0006414/ 0005525/ 0006184/ 0005829/ 0005853	GU339154	Fw: GCACCACGAAGCTTTAGCAG Rv: ACTGGAGTGTAACCGTTGGC	198	1.959

^a^ Gene name from the best BLAST hit or GO annotation

^b^ Accession number from the annotated sequence with the best BLAST hit

### Expression analysis of candidate genes

The expression levels of the seven candidate transcripts and the two reference genes (Table 3) were evaluated by means of qPCR to validate the RNA-Seq expression data. Our results showed a highly significant positive correlation between RNA-Seq and qPCR datasets in each paired-comparison: embryos*/* virgin female (Spearman Correlation; r = 0.87; *P* < 0.0001); embryos/ virgin male (Spearman Correlation; r = 0.93; *P* < 0.0001); and virgin male/ virgin female (Spearman Correlation; r = 0.87; *P* < 0.0001) (Additional File 8).

**Fig. 3:**
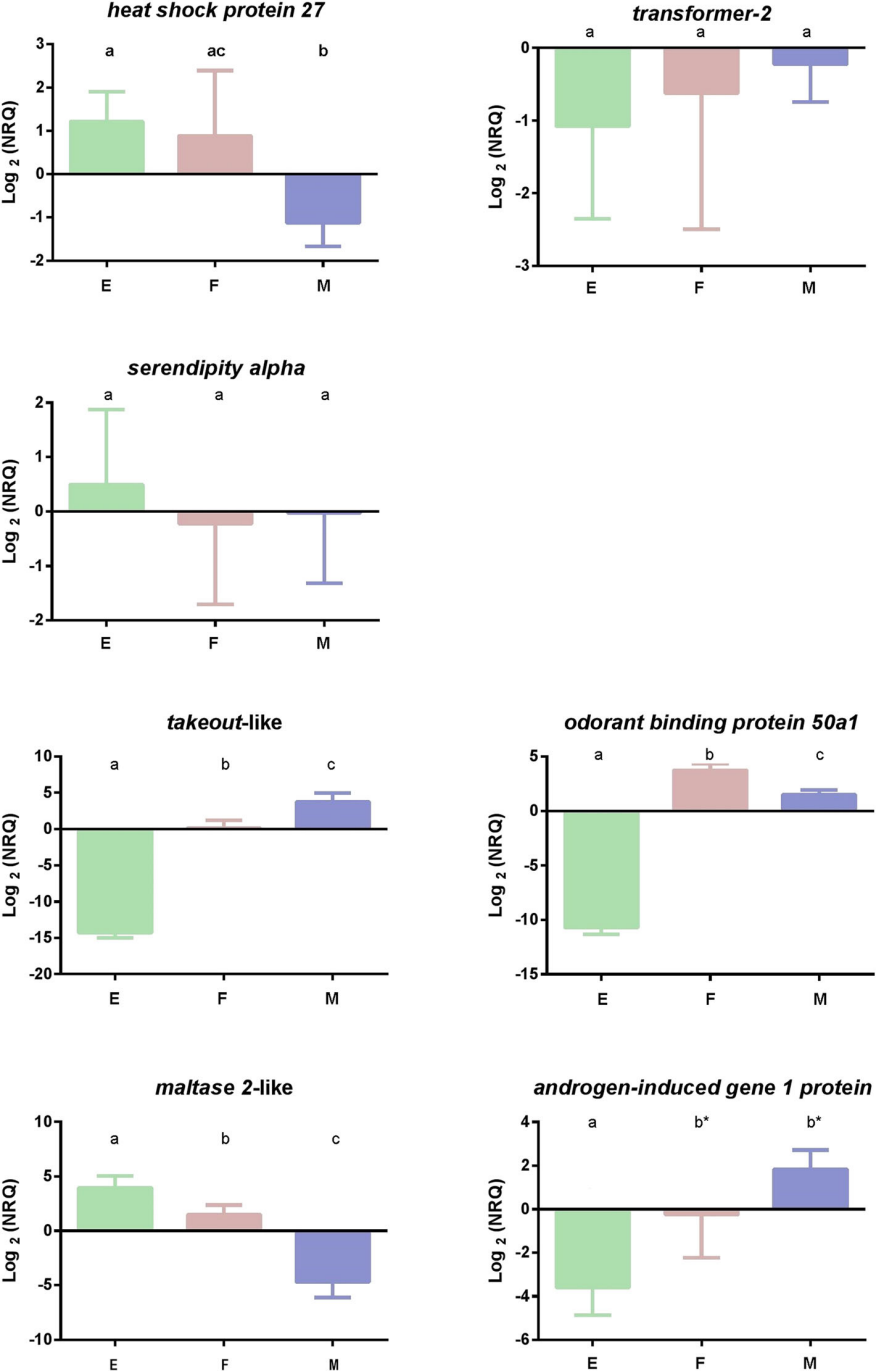
Comparative expression profiles of candidate genes obtained by qPCR among E (72 h embryos), sexually mature and virgin adults females (F) and males (M). NRQ are Expression Units. Different letters indicate significant differences (*P* < 0.05) between stages (t-test results). Letters marked with an asterisk (*) showed statistically marginal differences (P = 0.08). Reference genes for qPCR: *ribosomal protein L18* (*rpL18*) and *elongation factor-1a* (*ef-1a*)

The expression profiles obtained by qPCR evidenced an over-expression in embryos and females compared to males for the transcript annotated as *heat shock protein 27*, as we expected. Conversely, *serendipity alpha* and *transformer-2* transcripts were equally expressed in the compared treatments (72 h embryos; sexually mature virgin females and males) (Fig. 3). Significant differences in gene expression were detected for transcripts annotated as *takeout*-like and *odorant-binding protein 50a1* between all compared treatments (Fig. 3). Specifically, *takeout*-like and *odorant-binding protein 50a1* were under-expressed in embryos, compared to adults. Males showed a higher expression level than females in the case of *takeout*-like, and the opposite pattern was found for *odorant-binding protein 50a1.* The results of *maltase 2-*like transcript evidenced an over-expression in embryos relative to adults and in females relative to males (Fig. 3). Conversely, males evidenced greater levels of *androgen-induced gene 1 protein* in comparison to embryos and females, but the difference between sexes was only marginally significant (*P* = 0.08; Fig. 3).

**Fig. 4:**
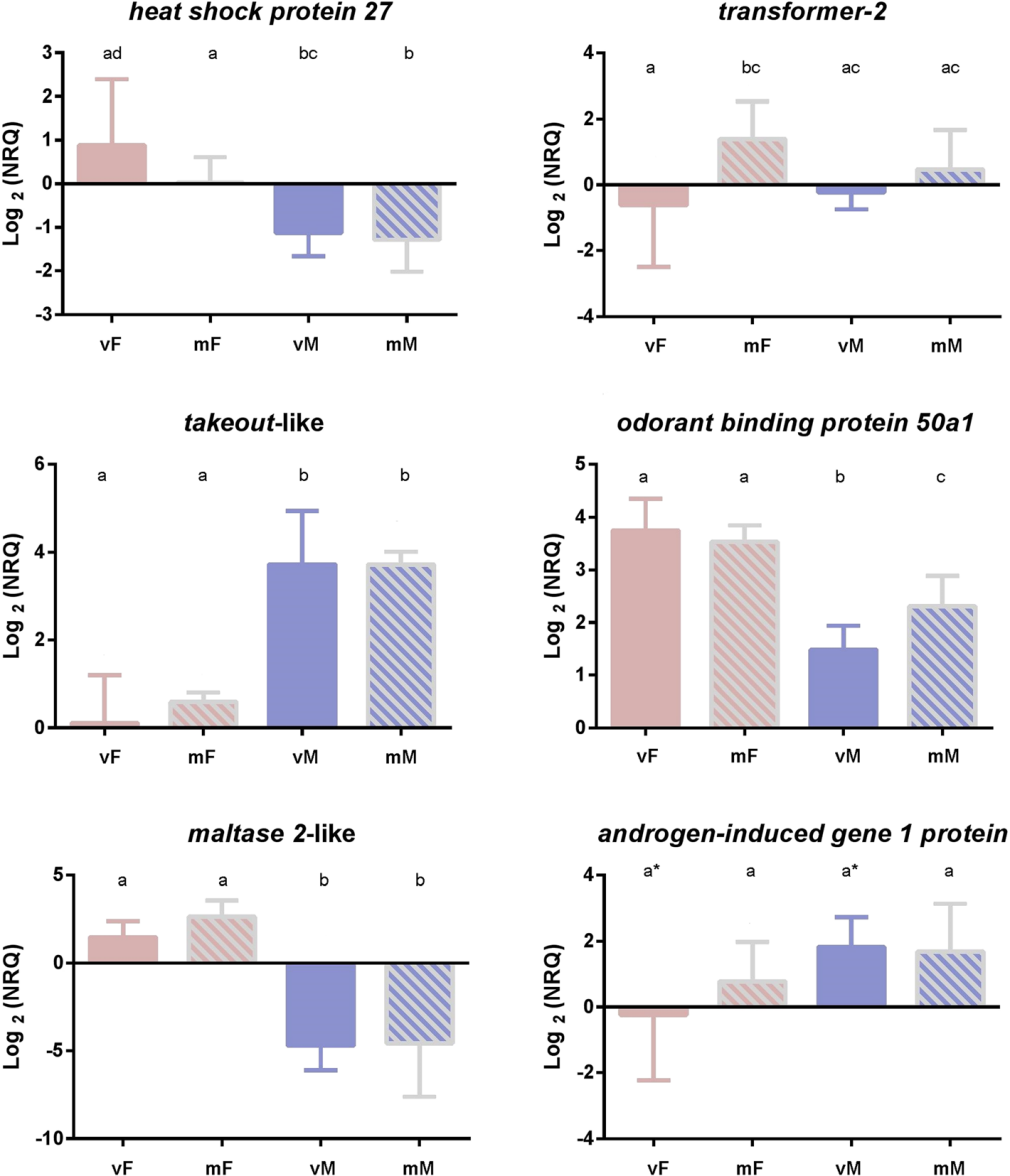
Comparative expression profiles of candidate genes obtained by qPCR among virgin vs mated females (vF, mF) and males (vM, mM). NRQ are Expression Units. Different letters indicate significant differences (*P* < 0.05) between treatments (t-test results). Letters marked with an asterisk (*) showed statistically marginal differences (*P* = 0.08). Reference genes for qPCR: *ribosomal protein L18* (*rpL18*) and *elongation factor-1a* (*ef-1a*)

The expression levels of most of the analyzed transcripts (except for *serendipity alpha,* which was not evaluated in this case) were similar between virgin and mated adults (Fig. 4). In particular, the expression levels of *transformer-2* and *odorant-binding protein 50a1* were higher in mated than virgin females and males, respectively (Fig. 4).

### Genetic diversity: prediction of molecular markers

**Fig. 5:**
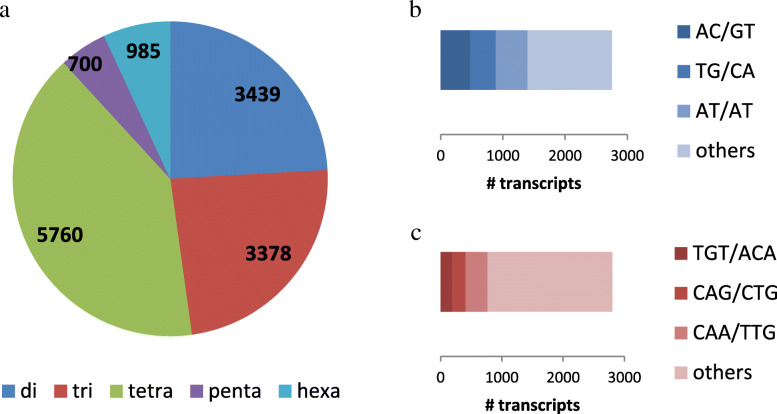
Microsatellite marker prediction. **a** Distribution of microsatellite motif by repeat type classes. Numbers represent percentage of transcripts within each repeat class (2: di-, 3: tri-, 4: tetra- and 5: pentanucleotide motifs, respectively). **b** Distribution and composition of di-nucleotide repeats. **c** Distribution and composition of tri-nucleotide repeats

After analyzing 86,925 sequences, we identified 12,956 simple sequence repeat (SSRs or microsatellites) motifs in the *A. fraterculus* sp. 1 transcriptome as potential molecular markers for this species. We detected 11,208 transcripts with at least one SSR motif and 2216 sequences with more than one SSR. In addition, 1306 SSRs were found in compound formation (including at least two different types of microsatellite motif) (Additional File 9). The most frequent type of microsatellite corresponded to tetranucleotide (40%), dinucleotide (24%) and trinucleotide (24%) repeats, with penta and hexanucleotide motifs (5 and 6%, respectively) being less abundant (Fig. 5a; Additional File 9).

The most frequent combinations for tetranucleotide motifs were TATG/CATA (12.4%) and ATAG/CTAT (8.4%); AT/AT (18.5%) and AC/GT (17.3%) for dinucleotide motif and CAA/TTG (12.9%) and CAG/CTG (7.8%) for trinucleotide repeats (Fig. 5b and c; Additional File 9). In addition, we performed in silico PCRs on all SRRs using the assembled transcriptome (in-house script). We obtained 10,968 SSRs (84.7%) that had adequate flanking sequences to allow the design of appropriate unique primers (Additional File 10).

## Discussion

In this study, we performed a comprehensive transcriptome analysis of *A. fraterculus* sp. 1 and utilized a bioinformatic approach to identify differentially expressed genes among males, females and embryos. We validated the in silico information by qPCR and compared the expression level of several candidate genes associated with development, communication and reproduction.

The transcriptome assembly showed 52,655 BLAST annotated transcripts and 28,756 sequences with an assigned GO term. BLASTX results showed a high number of hits with *C. capitata*, the best-annotated tephritid species, probably reflecting the presence of high quality and quantity of annotated sequences available on NCBI databases for this species. Conversely, when top BLAST hits were considered, *B. dorsalis* appeared as the most highly represented species, showing a considerable number of annotated sequences registered in the NCBI databases and a close phylogenetic relationship with the species under study. A gene ontology assessment revealed a good representation of GO terms in our transcriptome related to developmental process, behaviour and reproduction.

The analysis of predicted orthologs showed that the *A. fraterculus* sp. 1 transcriptome contains a quite complete and reliable database of transcripts. To further compare our assembly, we also performed a de novo transcriptome assembly adding an RNA-Seq dataset from the USDA-ARS (performed on the same *A. fraterculus* morphotype and with similar experimental design). Despite similar results in terms of BUSCO ortholog detection was obtained in the combined assembly, far fewer assembled and annotated transcripts were recovered. We suspect that datasets in which different populations are combined, can potentially generate quimeras and truncated transcripts that overwhelm the assembly process. Therefore, combining datasets from different populations and sequenced by different groups does not necessarily improve the yielded transcriptome assembly.

When comparisons were made considering all predicted proteins from the genomes of three closely related species (*B. oleae, R. zephyria,* and *C. capitata*), *A. fraterculus* sp. 1 transcriptome showed a good sequence representation (indicated by BUSCO and OrthoMCL results). Furthermore, BUSCO analysis showed 33.4% of complete and duplicated transcripts, showing that the information is somewhat redundant, probably evidencing the presence of potential ambiguously identified isoforms or due to the presence of fragmented sequences. However, a very strict filtering process using EdgeR and a deep manual processing allowed us to unambiguously identify a set of transcripts associated with early development, sex determination, and physiological pathways related to courtship and communications, which were further evaluated by qPCR. In fact, we detected a high positive correlation between RNA-Seq and qPCR data which suggests our methodology was robust enough.

The expression analysis of selected transcripts by qPCR in 72 h embryos, as well as virgin and mated males and females of *A. fraterculus* sp. 1 showed differential patterns mainly associated with their developmental stage and physiological status. The *transformer-2* gene has been previously reported as an essential switch for the sex determination cascade in several dipteran insect species [30, 51–53]. Recently, Zheng et al. [54] found that this gene was up-regulated in sexually mature females of *B. dorsalis* compared to their newly emerged counterparts and showed a higher expression in ovaries than in other tissues. In the present study, we found overall low expression level of this gene in both 72 h embryos and virgin and mated adults of *A. fraterculus*. Interestingly, the expression level of *transformer-2* was higher in mated than in virgin females. This result is in line with Zheng et al. [54], who suggested that sex determination related genes, such as *transformer-2*, may also function during sex maturation. We identified in our transcriptome two other transcripts with high similarity to genes involved in sex-determination and developmental pathways (*serendipity alpha* and *heat shock protein 27*). While *serendipity alpha* showed early expression in *C. capitata* [55] and *B. oleae* [56], our expression analysis revealed similar and low levels of this transcript in both 72 h embryos and adults of *A. fraterculus.* The analysis of 72 h embryos may not be the proper developmental time to detect the expected high expression of *serendipity alpha* for this species, which it probably reduces during late embryogenesis, as was previously described for other tephritid species [52, 53, 55]. The *heat shock protein 27* transcript did show an over-expression in embryos relative to males, and in females relative to males, for both virgin and mated individuals. These results are in line with those obtained in *C. capitata* [57, 58], which showed high level of *heat shock protein 27* RNA in mature ovaries and newly laid eggs, possibly indicating that this early embryonic RNA comes from the mature eggs and remains at a high expression during late embryogenesis. In addition to the analysis of these transcripts, the screening of the *A. fraterculus* transcriptome allowed to identify the presence of other early genes involved in development and sex-determination pathways (*transformer*, *sex-lethal*, *daughterless*, *doublesex* and *slow-as-molasses*), all of which showed a low expression level at the stages evaluated in this study.

Two of the candidate genes (*takeout*-like and *odorant-binding protein 50a1*) involved in courtship [59–61] and mate choice [45, 62–65] in insects, respectively, showed very low expression in embryos and differences in their expression level between males and females of *A. fraterculus*. Specifically, the transcript annotated as *takeout*-like was over-expressed in males relative to females independently of their reproductive status (virgin vs mated individuals). While sexual maturation and mating in insects are generally accompanied by major physiological and behavioural changes [66, 67], in the present study we found no changes in *takeout*-like expression between virgin and mated flies, despite the physiological changes occurring after copulation. In fact, in a transcriptomic analysis performed on *C. capitata*, the *takeout* gene showed enriched transcript abundance in mature relative to virgin females [66]. In relation to the *odorant-binding protein 50a1* transcript, we found that, in both virgins and mated adults, this transcript was over-expressed in females compared to males. Our results showed non- statistically significant differences between virgin vs mated females, consistently with previous results obtained by Campanini et al. [45] for an unidentified *A. fraterculus* morphotype from Brazil. However, we did detect higher expression of this transcript in mated than in virgin males. Conversely, Campanini et al. [45] showed non-significant difference for this comparison. Interestingly, these authors suggested that the up-regulation in post-mating males observed for other several odorant-binding proteins in *A. fraterculus* would be related to the involvement of mature males in lek formation, also supported by previous study performed in *C. capitata* [66] even after initial mating. Present results support the hypothesis that *odorant-binding protein 50a1* may be associated with courtship and mating in *A. fraterculus* sp.1, as proposed for other odorant-binding proteins for which expression level changed after mating. It is worth noting that our results were obtained by analyzing the gene expression in a narrow post-copulation temporal window of whole-body adults. Further studies in this *A. fraterculus* morphotype should include the analysis of different tissues and maturation statuses as well as a wider pre- and post-mating temporal window. Studying these conditions will shed light on the effect of the expression of *odorant-binding protein 50a1* and *takeout-*like genes, along with other selected genes (e.g. transcripts associated with response to stimulus, odorant receptors, co-receptors, chemosensory proteins) on the physiological modifications associated with the mating behaviour in males and females of *A. fraterculus.* In addition, the expression profile and physiological role of other related odorant-binding proteins and their potential isoforms must be further evaluated.

Finally, we analyzed the expression profiles of two genes associated with metabolic processes (*maltase 2*-like and *androgen-induced gene 1*). The transcript annotated as *maltase 2-*like evidenced high homology with the predicted *maltase-2-*like of *Z. cucurbitae* and also showed considerable similitude with the gene *maltase B2* of *D. melanogaster* involved in the metabolism of D-glucose [68]. The analysis of this transcript showed differential expression between embryos and male and female adults. In particular, 72 h embryos and females exhibited higher level of this transcript in comparison to males, probably indicating the differential energetic demands of the embryos and females. Interestingly, a member of the same family, maltase-D (A2 in *D. melanogaster*) is the most up-regulated gene in males after mating in *B. dorsalis* [69]. Although no differences were observed between virgin and mated individuals of both sexes, further research is needed to determine whether *maltase 2-*like could also have a role in mating in *A. fraterculus.* With respect to the *androgen-induced gene 1,* our results showed an over-expression of this transcript in males relative to embryos, but no significant differences between the sexes or physiological status (virgin vs mated individuals). This gene was found to be associated with the regulation of processes in the male’s accessory glands, enhanced by mating, and in combination with the bone morphogenetic protein (BMP) and the ecdysone receptor (EcR) [70]. Regarding tephritid flies, *B. dorsalis* is the species for which this gene was studied, and six isoforms have been identified [71, 72], but no functional characterization has been performed to date. Although we detected and analyzed only one isoform for this specific gene (*androgen-induced gene 1* X1 isoform), isoforms of genes of interest should be considered, in general, for further physiological and functional studies in order to distinguish their action separately.

A final contribution of our transcriptome analysis is the discovery of thousands of microsatellite markers. Here we described the presence of more than 11,000 transcripts containing microsatellite motifs, with the potential to be used in the characterization of the genetic variability of natural and laboratory populations of *A. fraterculus* from Argentina, and these markers can be added to those previously developed for this species [73–75]. These markers could also be useful in developing diagnostic molecular tools to analyze the genetic quality of laboratory populations and mass rearing production of this pest species (e.g. inbreeding or deleterious genotype analyses) and the incidence of evolutionary forces, such as selection and genetic drift acting in conjunction with ecological processes [76–78]. Furthermore, these transcriptome-derived microsatellite markers (commonly situated close to or within genes) increase their cross-species transferability, potentially being of help to elucidate the cryptic species within the *A. fraterculus* complex if more genomic information is generated from *A. fraterculus* morphotypes and close related species.

## Conclusion

Here we present a fairly complete *A. fraterculus* sp. 1 transcriptome of 72 h embryos and adults. The identification of transcripts and the expression analysis of candidate genes potentially involved in early development, reproduction and courtship provide a resource for future isolation and functional characterization of genes underlying relevant metabolic pathways, expanding our knowledge on physiological aspects of this pest species and also supporting the development of genetic control strategies. In addition, the set of identified microsatellites provides potentially polymorphic markers useful to monitor the pest in the field and supports the characterization of laboratory strains for SIT purpose.

## Methods

### Fly culture and stocks

*A. fraterculus* IGEAF laboratory strain kept at the National Institute of Agricultural Technology (INTA) (Hurlingham, Buenos Aires, Argentina) was used in this study. It was established in 2007 with approximately 10,000 *A. fraterculus* sp. 1 pupae and maintained under standard rearing conditions for 120 generations, following the procedures described by Vera et al. [79] and Jaldo et al. [80] without refreshing (i.e. no wild material was introduced to refresh the genetic background). This laboratory strain was derived from a semi-mass rearing colony kept at Estación Experimental Agroindustrial Obispo Colombres, Tucumán, Argentina, initiated in 1997 with wild pupae recovered from infested guavas (*Psidium guajava L.*) collected in the vicinity of Tafí Viejo, Tucumán, Argentina [81]. The strains were identified as *A. fraterculus* by Dr. R. Zucchi and Dr. V. Hernandez-Ortiz.

### Adult and egg collection

*A. fraterculus* sp. 1 eggs were collected from 15-day-old mated females following standard procedures [79]. Briefly, adults were exposed to oviposition units for 30 min and then the eggs were collected, washed with tap water, and immersed in water in an incubator with agitation for 72 h. Next, 72 h embryos were immediately processed for RNA isolation and transcriptome analysis. This procedure was repeated increasing the number of eggs collected in order to obtain the adequate quantity of eggs to obtain four replicates for qPCR assays.

For adult collection, 30 *A. fraterculus* sp. 1pupae were recovered from the laboratory experimental rearing. Upon emergence, males and females were separated and kept in 1 L glass flask with water and food [79, 80]. Five 15-day-old adults (sexually mature unmated males and females) were randomly sampled and processed for RNA isolation and transcriptome analysis. This procedure was repeated increasing the number of pupae recovered in order to obtain the adequate quantity of adults to obtain four replicates of each sex for qPCR assays.

### RNA isolation and libraries preparation for RNA-Seq analysis

Total RNA was isolated from two pools of five adult individuals (one pool of each sex; whole-body adult insects) and a pool of 25 mg of 72 h embryos. The TRIzol® Reagent (Thermo Fisher Scientific) was used for RNA isolation following the manufacturer’s instructions. The integrity of RNA was assessed by 1% w/v agarose gel electrophoresis, and the quantity and quality of all RNA samples were evaluated using a spectrophotometer (NanoDrop1000, Thermo Fisher Scientific) and a fluorometer (Qubit 4, Thermo Fisher Scientific) at the Genomic Unit UGB (Instituto de Biotecnología, IABIMO, INTA - CONICET).

Approximately 50 ng of total RNA from whole-body adults (virgin females, virgin males) and eggs (72 h embryos) of *A. fraterculus* were used for library construction (Illumina TruSeq Stranded mRNA kit) and subsequent sequencing via Illumina MiSeq platform. Paired-end reads of 2 × 150 bases were generated for further analysis and processed at the Genomic Unit (UGB, Instituto de Biotecnología, IABIMO, INTA - CONICET).

### Bioinformatic analysis

After raw reads were obtained, quality visualized with FastQC [82]. Trimmomatic [83] was used to trim the low-quality ends of all reads and adapter sequences and to discard low-quality reads. Briefly, the trimming process included the following steps: i) Remove adapters (TruSeq2-PE), leading low quality or N bases (below quality 3; LEADING: 3) and trailing low quality or N bases (below quality 3; TRAILING: 3); ii) Scan the read with a 4-base wide sliding window, cutting when the average quality per base drops below 15; iii) Drop reads below the 50 bases long; and iv) Remove the first 11 bases from reads. The remaining reads were merged into one longer read if both paired reads could align with each other using the Flash software [84]. All paired and unpaired reads of the libraries were assembled to construct the reference transcriptome using the Trinity v2.1.1 assembler [85] with a k-mer of 25, a max_memory 100G, and 10 CPU max usage.

The *A. fraterculus* assembly was evaluated using the BUSCO (benchmarking universal single-copy orthologs) software with the Diptera database (version 2019-11-20), which uses 2799 near-universal single-copy orthologs to assess the relative completeness of genome and transcriptome assemblies [86].

Annotation of the assembled sequences was obtained by aligning to the NCBI non-redundant (nr) protein database using BLASTX [87] and collecting the GO annotations with the BLAST2GO tool [88].

### Ortholog prediction and quality analysis of *A. fraterculus* transcriptome data

#### Selection of closely related species

A selection of related Tephritidae species was done considering previous phylogenetic studies of the family [48, 49] and organisms which fit the following criteria: i) Available and fully annotated genome (RefSeq Genomes DB NCBI) (https://www.ncbi.nlm.nih.gov/datasets/genomes/?txid=7211&source_db=RefSeq); ii) One species from each genus; and iii) Available 18S rRNA nucleotide sequence (BLASTN search; RefSeq Genomes DB; Query: 18S rRNA gene complete sequence [AF187101.2]); considering E value, % of identity and query coverage parameters) (Additional File 3).

All predicted proteins were identified using the TransDecoder software with default parameters and each predicted protein was at least 100 amino acids long. The general ortholog prediction was performed with OrthoMCL software using the complete proteomes of selected closely related Tephritidae species.

### Analysis of differentially expressed transcripts

Low-quality bases (average Q-score below 20) and adapter sequences were trimmed, and sequences less than 36 bases long were removed with Trimmomatic [83]. The remaining reads were mapped to the assembled transcriptome with Bowtie2, using the global-sensitive parameter and allowing all multi-mapping reads to be mapped [89]. The expectation-maximization (EM) algorithm of eXpress was used for more reliable transcriptome quantification [90]. Differential gene expression analysis was performed with the program EdgeR [50]. Briefly, transcripts with less than 10 counts per million (cpm) in one or more samples were filtered out. We performed normalization of the data using the trimmed mean of M-values (TMM) method. Read counts for each gene were fit to a negative binomial model and gene-wise exact tests were computed for differences in the means between two groups using the exact-Test function of EdgeR. As was previously mentioned, we set the common Biological coefficient of variation (bcv) value to 0.4 as proposed for an experimental design without biological replicates. Typical values for the common bcv (square- root-dispersion) range from 0.4 in human experiments to 0.1 for data on genetically identical model organisms [50]. Transcripts were considered differentially expressed between groups if they had a *P* value corrected by False Discovery Rate (FDR) lower than 0.05.

### Selection of candidate genes for qPCR analysis

We performed a filtering process based on the following criteria: transcripts with more than 10 counts per million (cpm > 10) in paired-comparisons between libraries; a threshold criterion of FC (fold change, defined as a ratio of cpm between compared libraries) with FC > 10 for over-representation and FC < 0.1 for under-representation; the annotation (or predicted function) considering the GO groups assigned, and/or the best BLAST hit searches.

The selection of transcripts was based on differences in the number of counts per million (cpm) obtained in paired-comparisons between libraries and their predicted function (Additional File 6 B). In addition, our transcriptome was screened for genes previously reported in the literature associated with reproduction and early expression (not present in the database mentioned above) based on previous studies performed in related species (Additional File 7 A and B).

### Differential expression analysis of selected transcripts by qPCR

For the expression analysis of selected candidate genes (*heat shock protein 27*, *odorant-binding protein 50a1*, *transformer-2*, *takeout*-like, *maltase 2-*like, *androgen-induced gene 1 protein* and *serendipity alpha*), total RNA was extracted from four pools of five sexually mature 15-day-old unmated males, five sexually mature 15-day-old unmated females (four biological replicates of each sex) and four pools of 72 h embryos (25 mg each). To obtain these biological replicates of 72 h embryos and adults, we followed the same procedures described above (adult and egg collection). In addition, the expression analysis of a subset of the selected candidate genes (*heat shock protein 27*, *odorant-binding protein 50a1*, *transformer-2*, *takeout-*like, *maltase 2-*like, *androgen-induced gene 1 protein*) was compared between virgin and mated sexually mature females and males. Briefly, sexually mature 15-day-old individuals of both sexes were mixed in the same glass flask and allowed to mate. Each couple were separated from the flask and observed. The couples for which copulation lasted more than 30 min were considered “mated”. After 2 h, the insects were processed for RNA isolation. Four pools of five mated females and five mated males were prepared (four biological pool replicates of each sex). RNA was extracted using TRIzol®Reagent (Thermo Fisher Scientific).

The resultant RNA was resuspended in 20 μl of DEPC-treated water. The quantity and quality of RNA were assessed using a Nanodrop spectrophotometer (Thermo Scientific Nanodrop 1000) and agarose gel electrophoresis (1% w/v). About 1 μg of total RNA was used as a template to synthesize first-strand cDNA using ImProm-II Reverse Transcriptase (Promega) and Oligo (dT) primers (Promega), following the manufacturer’s protocol. The resultant cDNA was diluted 1/10 for further use in real-time quantitative PCR (qPCR) experiments.

Specific primers for the amplification of candidate genes were designed by Primer-BLAST (http://www.ncbi.nlm.nih.gov/tools/primer-BLAST) from the selected transcripts (Table 3; Additional File 7 A and B).

The qPCR assays were performed in a Light Cycler 96 (Roche), using the cDNA as a template. Each reaction was performed in a total volume of 10 μl, containing 5 μl of Fast Start Essential DNA Green Master (Roche), 0.25 μl (50 mM) of each forward and reverse primers, 4 μl of ultrapure H_2_O (Roche), and 0.5 μl of cDNA template (1:10 dilution). The cycling parameters were 95 °C for 5 min followed by 40 cycles of 95 °C for 10 s and 60 °C for 45 s, ending with a melting curve product amplification. All qPCRs were performed in triplicate (i.e. three technical replicates). A total of four biological replicates for each of the five groups (72 h embryos, unmated females, unmated males, mated females, mated males) were performed. Expression values were calculated relative to the reference *rpL18* and *ef-1a* genes (Table 3) based on the recommendations made by Campanini et al. [45]. The stability and suitability of these reference genes were evaluated with the BestKeeper algorithm [91].

To analyze the expression profiles of the candidate genes, we applied the NRQ model, which consists in converting quantification cycle values (Cq) into NRQ (normalized relative quantities) values, adjusting for differences in PCR efficiency between the amplicons [92] and normalizing the data using multiple reference genes [93]. We calculated the relative quantities and normalized the data following the formulas in [93]. Differences in log (NRQ) between groups were evaluated using the t-test.

### Validation of transcriptome expression data with qPCR

Gene expression levels obtained by RNA-Seq and qPCR techniques for the nine transcripts (Table 3) were compared using their Fold Change (FC) values and analyzed with the Spearman Rank Correlation index to observe consistency. Correlations were performed for each of the three comparisons (72 h embryos vs virgin females, 72 h embryos vs virgin males, virgin males vs virgin females) using the transcript log2 FC for RNA-Seq and the mean NRQ log2 FC for qPCR data. Correlation coefficient r and *P* values were obtained for each comparison. Data used to generate FC values are listed in Additional File 7 A and B (RNA-Seq) and Fig. 3 (qPCR).

### SSR discovery

To identify SSRs markers, we ran the software MISA [http://pgrc.ipk-gatersleben.de/misa/misa.html]. The criteria used for SSR selection were based on the minimum number of repeats as follows: five for dinucleotide, four for trinucleotide, three for tetra, penta, and hexanucleotide motifs.

To design pairs of primers with no genome information available that amplify the corresponding SSR marker in every transcript, we ran an in-house script that uses EPRIMER32 from the EMBOSS package (http://emboss.open-bio.org/wiki/Appdocs). An in silico PCR was performed on all SSR using the assembled transcriptome (in-house script).

## Supplementary Information

**Additional file 1. ***A. fraterculus* sp. 1 transcripts GO assignment. The results of level 3 GO annotation are summarized in the three main categories “cellular component”, “molecular function” and “biological process”. A total of 28,756 transcripts were assigned. The percentage (left Y-axis) and total number (right Y-axis) of transcripts in each category are shown. Y-axes are in log (10) scale. WEGO was used to produce the graph.

**Additional file 2. **Gene Ontology (GO) analysis of *A. fraterculus* sp. 1 transcripts. The table shows main terms at the second and third GO levels involved in biological processes, development, behaviour, and reproduction.

**Additional file 3.** Sequence information of related Tephritidae species available in the NCBI RefSeq_Genomes database.

**Additional file 4. **Prediction of orthologs from closely related species (*B. oleae* and *R. zephyria*) and the best-annotated tephritid species (*C. capitata*).

**Additional file 5. **EdgeR results. Each Excel sheet shows the paired-comparison between libraries (72 h embryo vs male; female vs male; and female vs 72 h embryo). LogFC: logarithm of the fold change for each contrast performed; LogCPM: logarithm of the counts per million for each transcript; *P* value: statistical P value for the individual test performed; FDR: false discovery rate (adjusted P value considering multiple testing).

**Additional file 6. A.** List of differentially expressed transcripts obtained in paired-comparisons of libraries. **A.1.** 72 h embryos vs female (**A.1.1.** transcripts over-expressed in embryos; **A.1.2.** transcripts over-expressed in females); **A.2**. 72 h embryos vs male (**A.2.1.** transcripts over-expressed in embryos; **A.2.2.** transcripts over-expressed in males); and **A.3.** female vs male (**A.3.1.** transcripts over-expressed in females; **A.3.2.** transcripts over-expressed in males). **B.** Functional annotation of differentially expressed transcripts between libraries. Only transcripts with CPM > 10 and 70–100% complete sequence (related to the best BLAST hit) are shown. Over-expressed transcripts: sequences with FC > 10; Under-expressed transcripts: sequences with FC < 0.1.

**Additional file 7. **Selection of putative transcripts associated with function of interest. **A.** Transcripts selected according to their differential expression among libraries and predicted function (GO or BLAST hit). **B.** Transcript selected according to the literature associated to early development, sex determination, and communication.

**Additional file 8. **Correlation between RNA-Seq and qPCR datasets for the nine analyzed transcripts (Table 3) in 72 h embryos, virgin males and virgin females of *A. fraterculus*. For each comparison, transcripts log2 FC from RNA-Seq analysis are plotted against the mean NRQs log2 fold change from qPCR analysis. Spearman’s rank correlation coefficients (r) for each comparison are shown. For the three comparisons, r values were highly significant (*P* < 0.0001) and demonstrate a high degree of correlation between the two datasets. Data used to create these graphs are detailed in Additional File 7 (RNA-Seq data) and Fig. 4 (qPCR data).

**Additional file 9.** List of transcripts containing microsatellite motifs and description by repeat type classes and number of repeating motif.

**Additional file 10.** List of transcripts containing microsatellite motif and primers designed available to be tested.

## Data Availability

Raw sequence reads of *A. fraterculus* transcriptome have been submitted to NCBI Sequence Read Archive (SRA) under the accession number of BioProject ID: PRJNA606292 (SRR11095951–53).

## Abbreviations

RNA-seq: Whole transcriptome sequencing
SIT: Sterile Insect Technique
GSS: Genetic sexing strain
CRISPR: Clustered regularly interspaced short palindromic repeats
GO: Gene ontology
qPCR: quantitative PCR
bp: base pairs
BLAST: Basic Local Alignment Search Tool
NR: Non-redundant
NCBI: National Center for Biotechnology Information
BUSCO: Benchmarking universal single-copy orthologs
ORFs: Open reading frames
DE: Differential expressed
Cpm: Counts per million
FC: Fold-change
SSR: Simple sequence repeat
BMP: Bone morphogenetic protein
EcR: Ecdysone receptor
rRNA: ribosomal RNA
TMM: Trimmed mean of M-values
bcv: Biological coefficient of variation
FDR: False Discovery Rate
NRQ: Normalized relative quantities
