# Supplementary material for: Transcriptome analysis of Anastrepha fraterculus sp. 1 males, females, and embryos: insights into development, courtship, and reproduction
Source: BMC Genet. 2020 Dec 18;21(Suppl 2):136. doi: 10.1186/s12863-020-00943-2 (PMC7747455; doi:10.1186/s12863-020-00943-2)
Supplement: Supplementary file 2 — Additional file 2. Gene Ontology (GO) analysis of A. fraterculus sp. 1 transcripts. The table shows main terms at the second and third GO levels involved in biological processes, development, behaviour, and reproduction. [file 12863_2020_943_MOESM2_ESM.docx]

| **Gene Ontology Terms** | | **# transcripts** |
| --- | --- | --- |
| GO:0009987 | *C*ellular process | 57 |
| GO:0007154 | - Cell communication | 10 |
| GO:0032502 | Developmental process | 4981 |
| GO:0007349 | - cellularization | 84 |
| GO:0009790 | - embryo development | 18 |
| GO:0007389 | - pattern specification process | 12 |
| GO:0048066 | - pigmentation during development | 17 |
| GO:0040029 | - regulation of gene expression, epigenetic | 2 |
| GO:0007530 | - sex determination | 4 |
| GO:0007548 | - sex differentiation | 7 |
| GO:0019827 | - stem cell maintenance | 12 |
| GO:0040007 | Growth | 30 |
| GO:0008152 | Metabolic process | 1955 |
| GO:0065007 | Biological regulation | 2 |
| GO:0050790 | - regulation of enzyme activity | 65 |
| GO:0000003 | - reproduction | 5 |
| GO:0050896 | - response to stimulus | 23 |
| GO:0006950 | - response to stress | 25 |
| GO:0050795 | - regulation of behaviour | 103 |
| GO:0007610 | Behaviour | 821 |
| GO:0060756 | - Foraging behaviour | 2 |
| GO:0019098 | - Reproductive behaviour | 114 |
| GO:0030534 | - Adult behaviour | 244 |
| GO:0007631 | - Feeding behaviour | 59 |
| GO:0007611 | - Learing or memory | 279 |
| GO:0007635 | - Chemosensory behaviour | 210 |
| GO:0048266 | - Behavioural response to pain | 2 |
| GO:0040040 | - Thermosensory behaviour | 20 |
| GO:0007638 | - Mechanosensory behaviour | 9 |
| GO:0051780 | - Behavioural response to nutrient | 2 |
| GO:0035640 | - Exploration behaviour | 1 |
| GO:0000003 | Reproduction | 1779 |
| GO:0022414 | - Reproductive process | 1713 |
| GO:0019953 | - Sexual reproduction | 1574 |
| GO:0022412 | - Cellular process involved in reproduction | 1454 |
| GO:0003006 | - Developmental process involved in reproduction | 1355 |
| GO:0045450 | - Bicoid mRNA localization | 13 |
| GO:2000241 | - Regulation of reproductive process | 161 |
| GO:2000243 | - Positive regulation of reproductive process | 26 |
| GO:0031128 | - developmental induction | 6 |
| GO:0009791 | - post-embryonic development | 1178 |
