# Supplementary material for: Transcriptome analysis of Anastrepha fraterculus sp. 1 males, females, and embryos: insights into development, courtship, and reproduction
Source: BMC Genet. 2020 Dec 18;21(Suppl 2):136. doi: 10.1186/s12863-020-00943-2 (PMC7747455; doi:10.1186/s12863-020-00943-2)
Supplement: Supplementary file 3 — Additional file 3. Sequence information of related Tephritidae species available in the NCBI RefSeq_Genomes database. [file 12863_2020_943_MOESM3_ESM.docx]

| **Species ID** | **Genome Assembly**  **(RefSeq ID)*** | ***18S rRNA -* BLASTN results**** | | | |
| --- | --- | --- | --- | --- | --- |
|  |  | **Top hits** | **Query Cover** | **E value** | **Per. ident** |
| *Bactrocera oleae*  olive fruit fly  Isolate: Demokritus | [GCF_001188975.1](https://www.ncbi.nlm.nih.gov/assembly/GCF_001188975.1/) | unplaced genomic scaffold/ NW_013582318.1 | 100 | 0.0 | 98.14 |
| *Rhagoletis zephyria*  snowberry fruit fly  Isolate: East Lansing | [GCF_001687245.1](https://www.ncbi.nlm.nih.gov/assembly/GCF_001687245.1/) | unplaced genomic scaffold/ NW_016169255.1 | 100 | 0.0 | 97.99 |
| *Ceratitis capitata* Mediterranean fruit fly | [GCF_000347755.3](https://www.ncbi.nlm.nih.gov/assembly/GCF_000347755.3/) | unplaced genomic scaffold/ NW_019376901.1 | 84 | 0.0 | 96.31 |
| *Zeugodacus cucurbitae*  melon fly  Strain: USDA-PBARC White Pupae T1 | [GCF_000806345.1](https://www.ncbi.nlm.nih.gov/assembly/GCF_000806345.1/) | unplaced genomic scaffold NW_011863795.1 | 35 | 0.0 | 94.11 |
| *Bactrocera latifrons*  Isolate: USDA-ARS-PBARC  rearing strain | [GCF_001853355.1](https://www.ncbi.nlm.nih.gov/assembly/GCF_001853355.1/) | unplaced genomic scaffold NW_017537126.1 | 22 | 0.0 | 96.81 |
| *Bactrocera dorsalis* oriental fruit fly  Strain: Punador | [GCF_000789215.1](https://www.ncbi.nlm.nih.gov/assembly/GCF_000789215.1/) | - | - | - | - |

* ID of available genomes of Tephritidae species (NCBI RefSeq_genomes database).

** BLASTN searches against Tephritidae Refseq_genomes database (NCBI) using *A. fraterculus* 18S ribosomal RNA gene complete sequence (AF_187101.2) as a query.
