# Supplementary material for: Transcriptome analysis of Anastrepha fraterculus sp. 1 males, females, and embryos: insights into development, courtship, and reproduction
Source: BMC Genet. 2020 Dec 18;21(Suppl 2):136. doi: 10.1186/s12863-020-00943-2 (PMC7747455; doi:10.1186/s12863-020-00943-2)
Supplement: Supplementary file 4 — Additional file 4. Prediction of orthologs from closely related species (B. oleae and R. zephyria) and the best-annotated tephritid species (C. capitata). [file 12863_2020_943_MOESM4_ESM.docx]

| **Species** | **Total proteins** | **At least 1 ortholog** | **Proteins with orthologs in at least two species** | **Proteins with orthologs in at least three species** | **Orthologs quadruple reciprocal** |
| --- | --- | --- | --- | --- | --- |
| *A. fraterculus* | 38,933 | 18,496 | 16,621 | 14,742 | 14,119 |
| *B. oleae* | 18,715 | 16,849 | 15,618 | 11,651 | 10,746 |
| *R. zephyria* | 34,587 | 20,954 | 19,010 | 13,761 | 12,788 |
| *C. capitata* | 22,949 | 21,230 | 19,706 | 13,990 | 12,941 |
