# Supplementary material for: Transcriptome analysis of Anastrepha fraterculus sp. 1 males, females, and embryos: insights into development, courtship, and reproduction
Source: BMC Genet. 2020 Dec 18;21(Suppl 2):136. doi: 10.1186/s12863-020-00943-2 (PMC7747455; doi:10.1186/s12863-020-00943-2)
Supplement: Supplementary file 6 — Additional file 6. A. List of differentially expressed transcripts obtained in paired-comparisons of libraries. A.1. 72 h embryos vs female (A.1.1. transcripts over-expressed in embryos; A.1.2. transcripts over-expressed in females); A.2. 72 h embryos vs male (A.2.1. transcripts over-expressed in embryos; A.2.2. transcripts over-expressed in males); and A.3. female vs male (A.3.1. transcripts over-expressed in females; A.3.2. transcripts over-expressed in males). B. Functional annotation of differentially expressed transcripts between libraries. Only transcripts with CPM > 10 and 70–100% complete sequence (related to the best BLAST hit) are shown. Over-expressed transcripts: sequences with FC > 10; Under-expressed transcripts: sequences with FC < 0.1. [file 12863_2020_943_MOESM6_ESM.docx]

**List of transcript differentially represented between paired-comparison of libraries.**

**Manual filtering using a threshold criterion of 10 fold-change (FC>10) and 0.1 fold-change (FC<0.1)**

**A.1. Comparison: 72 h embryo *vs* female**

**A.1.1. Transcripts over represented in embryos**

| **Transcript ID** | **Female** | **Embryo** | **FC** |
| --- | --- | --- | --- |
| TRINITY_DN12470_c0_g2_i3 | 10 | 1810 | 181 |
| TRINITY_DN14948_c0_g8_i1 | 22 | 3065 | 139,318182 |
| TRINITY_DN14948_c0_g16_i1 | 118 | 14405 | 122,076271 |
| TRINITY_DN11027_c0_g1_i1 | 25 | 2211 | 88,44 |
| TRINITY_DN14141_c1_g8_i2 | 16 | 1409 | 88,0625 |
| TRINITY_DN15733_c4_g31_i2 | 55 | 3806 | 69,2 |
| TRINITY_DN13268_c0_g2_i1 | 22 | 1314 | 59,7272727 |
| TRINITY_DN16051_c0_g2_i1 | 24 | 1277 | 53,2083333 |
| TRINITY_DN6532_c0_g3_i3 | 44 | 1962 | 44,5909091 |
| TRINITY_DN14062_c0_g1_i13 | 14 | 513 | 36,6428571 |
| TRINITY_DN13677_c0_g10_i1 | 15 | 549 | 36,6 |
| TRINITY_DN15181_c2_g8_i2 | 48 | 1750 | 36,4583333 |
| TRINITY_DN10350_c0_g1_i5 | 11 | 394 | 35,8181818 |
| TRINITY_DN13560_c0_g1_i8 | 59 | 2081 | 35,2711864 |
| TRINITY_DN14385_c0_g3_i1 | 72 | 2258 | 31,3611111 |
| TRINITY_DN14497_c0_g6_i1 | 16 | 491 | 30,6875 |
| TRINITY_DN16256_c0_g13_i1 | 11 | 328 | 29,8181818 |
| TRINITY_DN17502_c0_g2_i1 | 10 | 293 | 29,3 |
| TRINITY_DN9887_c0_g1_i2 | 56 | 1596 | 28,5 |
| TRINITY_DN17414_c0_g5_i1 | 12 | 338 | 28,1666667 |
| TRINITY_DN16256_c0_g2_i1 | 27 | 744 | 27,5555556 |
| TRINITY_DN17414_c0_g4_i2 | 10 | 271 | 27,1 |
| TRINITY_DN14445_c1_g23_i1 | 52 | 1404 | 27 |
| TRINITY_DN17671_c4_g5_i1 | 21 | 557 | 26,5238095 |
| TRINITY_DN9325_c0_g1_i1 | 21 | 554 | 26,3809524 |
| TRINITY_DN15528_c0_g1_i1 | 11 | 288 | 26,1818182 |
| TRINITY_DN14418_c0_g2_i1 | 35 | 897 | 25,6285714 |
| TRINITY_DN14889_c1_g1_i1 | 233 | 5781 | 24,8111588 |
| TRINITY_DN15181_c2_g20_i1 | 15 | 364 | 24,2666667 |
| TRINITY_DN12629_c0_g1_i2 | 14 | 333 | 23,7857143 |
| TRINITY_DN13692_c0_g3_i1 | 113 | 2643 | 23,3893805 |
| TRINITY_DN14062_c0_g1_i9 | 11 | 253 | 23 |
| TRINITY_DN16851_c0_g3_i1 | 14 | 321 | 22,9285714 |
| TRINITY_DN12195_c0_g1_i1 | 13 | 297 | 22,8461538 |
| TRINITY_DN14990_c1_g1_i17 | 14 | 318 | 22,7142857 |
| TRINITY_DN16698_c0_g2_i2 | 16 | 360 | 22,5 |
| TRINITY_DN13095_c0_g2_i1 | 17 | 375 | 22,0588235 |
| TRINITY_DN15285_c0_g1_i2 | 157 | 3446 | 21,9490446 |
| TRINITY_DN17073_c0_g1_i2 | 11 | 238 | 21,6363636 |
| TRINITY_DN12137_c0_g10_i1 | 11 | 237 | 21,5454545 |
| TRINITY_DN17714_c0_g4_i1 | 13 | 279 | 21,4615385 |
| TRINITY_DN13552_c0_g2_i6 | 30 | 636 | 21,2 |
| TRINITY_DN10248_c0_g2_i1 | 12 | 253 | 21,0833333 |
| TRINITY_DN13343_c0_g2_i1 | 23 | 484 | 21,0434783 |
| TRINITY_DN13718_c0_g1_i2 | 10 | 209 | 20,9 |
| TRINITY_DN11311_c0_g1_i2 | 24 | 498 | 20,75 |
| TRINITY_DN12044_c0_g13_i1 | 40 | 824 | 20,6 |
| TRINITY_DN9709_c0_g1_i2 | 10 | 205 | 20,5 |
| TRINITY_DN17414_c0_g2_i1 | 95 | 1947 | 20,4947368 |
| TRINITY_DN13343_c0_g1_i1 | 18 | 367 | 20,3888889 |
| TRINITY_DN14382_c0_g1_i2 | 18 | 356 | 19,7777778 |
| TRINITY_DN12631_c1_g1_i1 | 69 | 1353 | 19,6086957 |
| TRINITY_DN10514_c0_g2_i1 | 14 | 273 | 19,5 |
| TRINITY_DN11957_c0_g2_i1 | 14 | 264 | 18,8571429 |
| TRINITY_DN17005_c0_g1_i1 | 11 | 205 | 18,6363636 |
| TRINITY_DN13268_c0_g5_i1 | 140 | 2466 | 17,6142857 |
| TRINITY_DN17105_c0_g7_i2 | 27 | 475 | 17,5925926 |
| TRINITY_DN12498_c0_g4_i1 | 11 | 191 | 17,3636364 |
| TRINITY_DN16256_c0_g11_i1 | 17 | 293 | 17,2352941 |
| TRINITY_DN13143_c0_g2_i3 | 20 | 344 | 17,2 |
| TRINITY_DN17247_c0_g1_i2 | 12 | 206 | 17,1666667 |
| TRINITY_DN16930_c0_g1_i3 | 15 | 255 | 17 |
| TRINITY_DN16851_c0_g2_i1 | 14 | 237 | 16,9285714 |
| TRINITY_DN13889_c0_g1_i4 | 10 | 169 | 16,9 |
| TRINITY_DN16820_c0_g2_i4 | 10 | 168 | 16,8 |
| TRINITY_DN17456_c0_g1_i3 | 31 | 513 | 16,5483871 |
| TRINITY_DN3817_c0_g1_i1 | 71 | 1137 | 16,0140845 |
| TRINITY_DN14715_c0_g1_i6 | 58 | 928 | 16 |
| TRINITY_DN16568_c0_g1_i8 | 10 | 159 | 15,9 |
| TRINITY_DN17728_c0_g3_i1 | 32 | 506 | 15,8125 |
| TRINITY_DN16015_c0_g3_i1 | 26 | 411 | 15,8076923 |
| TRINITY_DN10350_c0_g1_i3 | 47 | 742 | 15,787234 |
| TRINITY_DN14445_c1_g10_i1 | 14 | 218 | 15,5714286 |
| TRINITY_DN9228_c0_g2_i2 | 300 | 4648 | 15,4933333 |
| TRINITY_DN14637_c2_g3_i2 | 11 | 170 | 15,4545455 |
| TRINITY_DN9838_c0_g3_i2 | 29 | 446 | 15,3793103 |
| TRINITY_DN8632_c0_g1_i1 | 10 | 153 | 15,3 |
| TRINITY_DN17210_c1_g1_i3 | 15 | 227 | 15,1333333 |
| TRINITY_DN17339_c0_g7_i2 | 14 | 211 | 15,0714286 |
| TRINITY_DN12566_c0_g1_i3 | 16 | 241 | 15,0625 |
| TRINITY_DN16851_c0_g2_i2 | 351 | 5225 | 14,8860399 |
| TRINITY_DN17005_c0_g3_i1 | 24 | 354 | 14,75 |
| TRINITY_DN14889_c1_g1_i3 | 25 | 366 | 14,64 |
| TRINITY_DN6779_c0_g1_i1 | 13 | 190 | 14,6153846 |
| TRINITY_DN17315_c1_g8_i9 | 12 | 175 | 14,5833333 |
| TRINITY_DN11254_c0_g1_i4 | 25 | 363 | 14,52 |
| TRINITY_DN16256_c0_g8_i1 | 12 | 174 | 14,5 |
| TRINITY_DN12704_c0_g1_i2 | 515 | 7463 | 14,4912621 |
| TRINITY_DN16287_c0_g1_i1 | 190 | 2750 | 14,4736842 |
| TRINITY_DN16027_c2_g1_i7 | 110 | 1591 | 14,4636364 |
| TRINITY_DN14419_c0_g1_i1 | 11 | 158 | 14,3636364 |
| TRINITY_DN17414_c0_g13_i2 | 11 | 156 | 14,1818182 |
| TRINITY_DN14340_c2_g1_i2 | 29 | 410 | 14,137931 |
| TRINITY_DN13659_c0_g2_i1 | 34 | 476 | 14 |
| TRINITY_DN13268_c0_g4_i1 | 20 | 280 | 14 |
| TRINITY_DN15599_c0_g6_i1 | 12 | 167 | 13,9166667 |
| TRINITY_DN16374_c0_g14_i1 | 19 | 262 | 13,7894737 |
| TRINITY_DN12549_c1_g1_i1 | 37 | 510 | 13,7837838 |
| TRINITY_DN17007_c0_g1_i3 | 44 | 604 | 13,7272727 |
| TRINITY_DN11233_c0_g1_i1 | 11 | 151 | 13,7272727 |
| TRINITY_DN14178_c0_g1_i1 | 11 | 151 | 13,7272727 |
| TRINITY_DN24436_c0_g1_i1 | 12 | 164 | 13,6666667 |
| TRINITY_DN9611_c0_g1_i1 | 11 | 150 | 13,6363636 |
| TRINITY_DN13933_c0_g1_i2 | 11 | 150 | 13,6363636 |
| TRINITY_DN14062_c0_g1_i2 | 10 | 136 | 13,6 |
| TRINITY_DN14004_c0_g1_i1 | 32 | 424 | 13,25 |
| TRINITY_DN15431_c0_g1_i4 | 29 | 380 | 13,1034483 |
| TRINITY_DN8906_c0_g3_i1 | 14 | 183 | 13,0714286 |
| TRINITY_DN17339_c0_g8_i1 | 15 | 196 | 13,0666667 |
| TRINITY_DN11556_c1_g1_i2 | 16 | 209 | 13,0625 |
| TRINITY_DN15360_c0_g1_i2 | 22 | 287 | 13,0454545 |
| TRINITY_DN14178_c0_g1_i2 | 27 | 351 | 13 |
| TRINITY_DN12272_c0_g3_i1 | 17 | 221 | 13 |
| TRINITY_DN8975_c243_g1_i1 | 24 | 310 | 12,9166667 |
| TRINITY_DN13762_c0_g1_i1 | 22 | 284 | 12,9090909 |
| TRINITY_DN13268_c0_g7_i1 | 11 | 140 | 12,7272727 |
| TRINITY_DN15451_c3_g6_i1 | 240 | 3042 | 12,675 |
| TRINITY_DN17637_c0_g8_i2 | 15 | 190 | 12,6666667 |
| TRINITY_DN14074_c0_g1_i1 | 17 | 215 | 12,6470588 |
| TRINITY_DN16580_c0_g1_i3 | 17 | 215 | 12,6470588 |
| TRINITY_DN9904_c0_g3_i1 | 12 | 151 | 12,5833333 |
| TRINITY_DN9550_c0_g1_i1 | 20 | 249 | 12,45 |
| TRINITY_DN15469_c0_g1_i6 | 23 | 286 | 12,4347826 |
| TRINITY_DN14630_c0_g2_i6 | 31 | 383 | 12,3548387 |
| TRINITY_DN13771_c0_g1_i2 | 15 | 184 | 12,2666667 |
| TRINITY_DN17544_c0_g1_i7 | 13 | 159 | 12,2307692 |
| TRINITY_DN12498_c0_g1_i1 | 17 | 207 | 12,1764706 |
| TRINITY_DN14385_c0_g3_i2 | 12 | 146 | 12,1666667 |
| TRINITY_DN13156_c0_g1_i1 | 18 | 217 | 12,0555556 |
| TRINITY_DN15548_c0_g1_i3 | 41 | 493 | 12,0243902 |
| TRINITY_DN17297_c0_g5_i4 | 56 | 668 | 11,9285714 |
| TRINITY_DN11259_c0_g1_i2 | 902 | 10697 | 11,8592018 |
| TRINITY_DN15489_c1_g1_i1 | 38 | 449 | 11,8157895 |
| TRINITY_DN12524_c0_g1_i3 | 10 | 118 | 11,8 |
| TRINITY_DN2413_c0_g1_i1 | 22 | 259 | 11,7727273 |
| TRINITY_DN30172_c0_g1_i1 | 25 | 294 | 11,76 |
| TRINITY_DN15145_c0_g1_i4 | 12 | 141 | 11,75 |
| TRINITY_DN14886_c0_g3_i2 | 53 | 618 | 11,6603774 |
| TRINITY_DN15709_c0_g1_i7 | 62 | 722 | 11,6451613 |
| TRINITY_DN168_c0_g2_i1 | 16 | 186 | 11,625 |
| TRINITY_DN17542_c1_g3_i2 | 1274 | 14788 | 11,6075353 |
| TRINITY_DN16712_c0_g1_i6 | 12 | 139 | 11,5833333 |
| TRINITY_DN14307_c1_g4_i1 | 16 | 184 | 11,5 |
| TRINITY_DN17075_c1_g3_i4 | 19 | 217 | 11,4210526 |
| TRINITY_DN14295_c0_g1_i5 | 47 | 536 | 11,4042553 |
| TRINITY_DN16941_c0_g1_i9 | 58 | 659 | 11,362069 |
| TRINITY_DN9327_c1_g1_i3 | 18 | 204 | 11,3333333 |
| TRINITY_DN5599_c0_g1_i1 | 10 | 113 | 11,3 |
| TRINITY_DN25097_c0_g1_i1 | 13 | 146 | 11,2307692 |
| TRINITY_DN17315_c1_g8_i10 | 169 | 1896 | 11,2189349 |
| TRINITY_DN16168_c0_g6_i2 | 16 | 179 | 11,1875 |
| TRINITY_DN17734_c1_g7_i9 | 16 | 179 | 11,1875 |
| TRINITY_DN5597_c0_g1_i1 | 17 | 190 | 11,1764706 |
| TRINITY_DN15881_c0_g2_i1 | 38 | 423 | 11,1315789 |
| TRINITY_DN4827_c0_g1_i1 | 45 | 500 | 11,1111111 |
| TRINITY_DN9154_c0_g2_i3 | 10 | 111 | 11,1 |
| TRINITY_DN13103_c0_g1_i3 | 11 | 122 | 11,0909091 |
| TRINITY_DN2247_c0_g1_i1 | 11 | 122 | 11,0909091 |
| TRINITY_DN16273_c0_g1_i7 | 22 | 243 | 11,0454545 |
| TRINITY_DN14068_c0_g1_i3 | 12 | 132 | 11 |
| TRINITY_DN12498_c0_g3_i1 | 11 | 121 | 11 |
| TRINITY_DN11246_c0_g1_i1 | 10 | 110 | 11 |
| TRINITY_DN16027_c2_g1_i11 | 35 | 382 | 10,9142857 |
| TRINITY_DN13644_c0_g4_i1 | 11 | 120 | 10,9090909 |
| TRINITY_DN15268_c0_g3_i1 | 10 | 109 | 10,9 |
| TRINITY_DN11556_c1_g3_i1 | 47 | 512 | 10,893617 |
| TRINITY_DN15560_c0_g1_i1 | 15 | 163 | 10,8666667 |
| TRINITY_DN16753_c0_g1_i2 | 22 | 239 | 10,8636364 |
| TRINITY_DN16236_c0_g3_i2 | 16 | 173 | 10,8125 |
| TRINITY_DN15250_c0_g2_i1 | 12 | 129 | 10,75 |
| TRINITY_DN15178_c1_g5_i1 | 34 | 365 | 10,7352941 |
| TRINITY_DN12549_c0_g1_i2 | 44 | 472 | 10,7272727 |
| TRINITY_DN17544_c0_g1_i3 | 11 | 118 | 10,7272727 |
| TRINITY_DN17511_c0_g4_i2 | 10 | 107 | 10,7 |
| TRINITY_DN16633_c0_g3_i11 | 20 | 213 | 10,65 |
| TRINITY_DN15404_c0_g1_i2 | 11 | 116 | 10,5454545 |
| TRINITY_DN14432_c0_g1_i4 | 28 | 295 | 10,5357143 |
| TRINITY_DN9341_c0_g1_i1 | 40 | 421 | 10,525 |
| TRINITY_DN12916_c0_g2_i3 | 13 | 136 | 10,4615385 |
| TRINITY_DN15570_c0_g1_i2 | 33 | 345 | 10,4545455 |
| TRINITY_DN17008_c1_g1_i2 | 19 | 198 | 10,4210526 |
| TRINITY_DN16653_c3_g17_i1 | 18 | 186 | 10,3333333 |
| TRINITY_DN16057_c0_g1_i3 | 38 | 392 | 10,3157895 |
| TRINITY_DN15522_c0_g1_i2 | 12 | 123 | 10,25 |
| TRINITY_DN4728_c0_g1_i1 | 13 | 133 | 10,2307692 |
| TRINITY_DN9680_c0_g1_i1 | 66 | 674 | 10,2121212 |
| TRINITY_DN10422_c0_g2_i1 | 19 | 194 | 10,2105263 |
| TRINITY_DN11264_c0_g1_i2 | 15 | 153 | 10,2 |
| TRINITY_DN15250_c0_g3_i1 | 10 | 102 | 10,2 |
| TRINITY_DN17448_c0_g16_i2 | 12 | 122 | 10,1666667 |
| TRINITY_DN16229_c0_g4_i9 | 18 | 182 | 10,1111111 |
| TRINITY_DN16905_c0_g2_i7 | 25 | 251 | 10,04 |
| TRINITY_DN17315_c1_g8_i1 | 19 | 190 | 10 |
| TRINITY_DN17323_c0_g1_i1 | 15 | 150 | 10 |
| TRINITY_DN9381_c0_g1_i2 | 12 | 120 | 10 |

**A.1. Comparison: 72 h embryo *vs* female**

**A.1.2. Transcripts over represented in females**

| **Transcript ID** | **Female** | **Embryo** | **FC** |
| --- | --- | --- | --- |
| TRINITY_DN13610_c0_g2_i7 | 13202 | 17 | 0,00128768 |
| TRINITY_DN16414_c0_g1_i1 | 31892 | 43 | 0,0013483 |
| TRINITY_DN13678_c0_g1_i1 | 20264 | 30 | 0,00148046 |
| TRINITY_DN13647_c0_g1_i6 | 6708 | 10 | 0,00149076 |
| TRINITY_DN12145_c0_g1_i3 | 7214 | 11 | 0,00152481 |
| TRINITY_DN16414_c0_g1_i2 | 3436 | 10 | 0,00291036 |
| TRINITY_DN17060_c1_g7_i4 | 2670 | 11 | 0,00411985 |
| TRINITY_DN14543_c0_g1_i3 | 1867 | 11 | 0,00589181 |
| TRINITY_DN11245_c0_g1_i1 | 1057 | 11 | 0,01040681 |
| TRINITY_DN15068_c0_g1_i4 | 1001 | 11 | 0,01098901 |
| TRINITY_DN13190_c0_g1_i1 | 6328 | 73 | 0,01153603 |
| TRINITY_DN12780_c0_g1_i1 | 2844 | 34 | 0,01195499 |
| TRINITY_DN17415_c0_g1_i5 | 1641 | 23 | 0,01401584 |
| TRINITY_DN14543_c0_g1_i2 | 617 | 10 | 0,01620746 |
| TRINITY_DN8182_c0_g1_i1 | 983 | 16 | 0,0162767 |
| TRINITY_DN17307_c1_g1_i1 | 768 | 13 | 0,01692708 |
| TRINITY_DN16480_c0_g3_i3 | 5506 | 95 | 0,0172539 |
| TRINITY_DN10865_c0_g29_i1 | 568 | 10 | 0,01760563 |
| TRINITY_DN14198_c3_g9_i1 | 2805 | 51 | 0,01818182 |
| TRINITY_DN15542_c0_g1_i3 | 900 | 17 | 0,01888889 |
| TRINITY_DN17060_c1_g4_i2 | 2729 | 52 | 0,0190546 |
| TRINITY_DN15356_c0_g1_i2 | 498 | 11 | 0,02208835 |
| TRINITY_DN10429_c0_g16_i1 | 560 | 13 | 0,02321429 |
| TRINITY_DN15631_c0_g1_i1 | 2625 | 61 | 0,0232381 |
| TRINITY_DN9616_c0_g1_i1 | 957 | 23 | 0,02403344 |
| TRINITY_DN13272_c0_g1_i6 | 420 | 12 | 0,02857143 |
| TRINITY_DN15806_c9_g4_i2 | 473 | 14 | 0,02959831 |
| TRINITY_DN14198_c2_g1_i1 | 639 | 19 | 0,02973396 |
| TRINITY_DN17706_c1_g18_i1 | 763 | 23 | 0,03014417 |
| TRINITY_DN16647_c0_g2_i1 | 481 | 15 | 0,03118503 |
| TRINITY_DN10357_c0_g1_i1 | 320 | 10 | 0,03125 |
| TRINITY_DN15804_c0_g1_i1 | 651 | 21 | 0,03225806 |
| TRINITY_DN14396_c0_g2_i3 | 924 | 30 | 0,03246753 |
| TRINITY_DN15804_c0_g2_i1 | 1197 | 40 | 0,03341688 |
| TRINITY_DN15139_c0_g3_i1 | 321 | 12 | 0,03738318 |
| TRINITY_DN11806_c0_g1_i1 | 1123 | 42 | 0,03739982 |
| TRINITY_DN9842_c0_g1_i2 | 293 | 11 | 0,03754266 |
| TRINITY_DN10960_c0_g2_i2 | 850 | 34 | 0,04 |
| TRINITY_DN16718_c1_g1_i1 | 572 | 23 | 0,04020979 |
| TRINITY_DN15627_c0_g1_i2 | 341 | 14 | 0,04105572 |
| TRINITY_DN16247_c1_g2_i9 | 412 | 17 | 0,04126214 |
| TRINITY_DN9911_c0_g1_i3 | 261 | 11 | 0,04214559 |
| TRINITY_DN13101_c0_g3_i2 | 2344 | 99 | 0,04223549 |
| TRINITY_DN17164_c0_g1_i2 | 1062 | 46 | 0,0433145 |
| TRINITY_DN527_c0_g2_i1 | 385 | 17 | 0,04415584 |
| TRINITY_DN15806_c9_g3_i1 | 8499 | 380 | 0,04471114 |
| TRINITY_DN15604_c0_g2_i3 | 948 | 43 | 0,04535865 |
| TRINITY_DN11302_c0_g1_i3 | 209 | 10 | 0,04784689 |
| TRINITY_DN14198_c1_g1_i2 | 532 | 26 | 0,04887218 |
| TRINITY_DN15227_c0_g1_i4 | 978 | 48 | 0,04907975 |
| TRINITY_DN15609_c0_g2_i1 | 902 | 45 | 0,04988914 |
| TRINITY_DN14569_c0_g1_i1 | 239 | 12 | 0,05020921 |
| TRINITY_DN10390_c0_g1_i1 | 26776 | 1373 | 0,05127726 |
| TRINITY_DN27424_c0_g1_i1 | 439 | 23 | 0,0523918 |
| TRINITY_DN14420_c0_g1_i6 | 303 | 16 | 0,05280528 |
| TRINITY_DN10160_c0_g1_i1 | 774 | 41 | 0,05297158 |
| TRINITY_DN15806_c9_g8_i1 | 1144 | 61 | 0,05332168 |
| TRINITY_DN15163_c0_g5_i1 | 291 | 16 | 0,05498282 |
| TRINITY_DN16226_c0_g2_i2 | 5934 | 333 | 0,05611729 |
| TRINITY_DN14240_c0_g1_i2 | 938 | 53 | 0,0565032 |
| TRINITY_DN14350_c1_g2_i3 | 224 | 13 | 0,05803571 |
| TRINITY_DN17494_c0_g1_i3 | 549 | 32 | 0,0582878 |
| TRINITY_DN17341_c0_g1_i6 | 265 | 16 | 0,06037736 |
| TRINITY_DN14966_c1_g7_i2 | 160 | 10 | 0,0625 |
| TRINITY_DN13275_c0_g3_i3 | 158 | 10 | 0,06329114 |
| TRINITY_DN10688_c1_g12_i1 | 205 | 13 | 0,06341463 |
| TRINITY_DN13190_c0_g1_i2 | 172 | 11 | 0,06395349 |
| TRINITY_DN9102_c0_g2_i1 | 170 | 11 | 0,06470588 |
| TRINITY_DN14483_c0_g1_i3 | 153 | 10 | 0,06535948 |
| TRINITY_DN10688_c1_g15_i1 | 363 | 24 | 0,0661157 |
| TRINITY_DN188_c0_g1_i1 | 211 | 14 | 0,06635071 |
| TRINITY_DN17691_c2_g4_i2 | 450 | 30 | 0,06666667 |
| TRINITY_DN11012_c0_g2_i5 | 223 | 15 | 0,06726457 |
| TRINITY_DN10167_c1_g1_i1 | 1587 | 107 | 0,06742281 |
| TRINITY_DN14656_c0_g1_i2 | 162 | 11 | 0,06790123 |
| TRINITY_DN10688_c1_g4_i1 | 146 | 10 | 0,06849315 |
| TRINITY_DN12706_c1_g1_i1 | 276 | 19 | 0,06884058 |
| TRINITY_DN13608_c0_g1_i1 | 789 | 55 | 0,06970849 |
| TRINITY_DN16334_c0_g2_i3 | 155 | 11 | 0,07096774 |
| TRINITY_DN19037_c0_g1_i1 | 6904 | 500 | 0,07242178 |
| TRINITY_DN14198_c3_g11_i1 | 512 | 38 | 0,07421875 |
| TRINITY_DN24620_c0_g1_i1 | 404 | 30 | 0,07425743 |
| TRINITY_DN16719_c0_g3_i3 | 377 | 28 | 0,07427056 |
| TRINITY_DN13449_c0_g4_i6 | 175 | 13 | 0,07428571 |
| TRINITY_DN13236_c0_g2_i9 | 200 | 15 | 0,075 |
| TRINITY_DN15258_c0_g2_i8 | 146 | 11 | 0,07534247 |
| TRINITY_DN15856_c0_g3_i1 | 132 | 10 | 0,07575758 |
| TRINITY_DN10186_c0_g1_i4 | 300 | 23 | 0,07666667 |
| TRINITY_DN10167_c1_g2_i1 | 399 | 31 | 0,07769424 |
| TRINITY_DN10429_c0_g2_i1 | 218 | 17 | 0,07798165 |
| TRINITY_DN15217_c0_g1_i1 | 128 | 10 | 0,078125 |
| TRINITY_DN17639_c1_g2_i9 | 305 | 24 | 0,07868852 |
| TRINITY_DN17444_c1_g14_i2 | 633 | 50 | 0,07898894 |
| TRINITY_DN15741_c0_g1_i3 | 291 | 23 | 0,0790378 |
| TRINITY_DN9816_c0_g1_i1 | 227 | 18 | 0,07929515 |
| TRINITY_DN17736_c0_g2_i8 | 275 | 22 | 0,08 |
| TRINITY_DN15745_c0_g1_i2 | 337 | 27 | 0,08011869 |
| TRINITY_DN14588_c1_g3_i1 | 1484 | 119 | 0,08018868 |
| TRINITY_DN16644_c0_g1_i7 | 171 | 14 | 0,08187135 |
| TRINITY_DN27389_c0_g1_i1 | 216 | 18 | 0,08333333 |
| TRINITY_DN17738_c0_g3_i1 | 3076 | 261 | 0,08485046 |
| TRINITY_DN16842_c0_g1_i4 | 137 | 12 | 0,08759124 |
| TRINITY_DN14402_c0_g2_i3 | 421 | 37 | 0,08788599 |
| TRINITY_DN24136_c0_g1_i1 | 147 | 13 | 0,08843537 |
| TRINITY_DN15650_c0_g3_i5 | 350 | 31 | 0,08857143 |
| TRINITY_DN17295_c2_g1_i7 | 315 | 28 | 0,08888889 |
| TRINITY_DN17551_c0_g3_i1 | 135 | 12 | 0,08888889 |
| TRINITY_DN17628_c0_g1_i4 | 1102 | 98 | 0,08892922 |
| TRINITY_DN13813_c0_g1_i1 | 168 | 15 | 0,08928571 |
| TRINITY_DN10722_c0_g3_i1 | 347 | 31 | 0,08933718 |
| TRINITY_DN23191_c0_g1_i1 | 134 | 12 | 0,08955224 |
| TRINITY_DN8717_c0_g10_i1 | 1205 | 108 | 0,08962656 |
| TRINITY_DN16026_c0_g1_i2 | 278 | 25 | 0,08992806 |
| TRINITY_DN17622_c1_g1_i6 | 120 | 11 | 0,09166667 |
| TRINITY_DN16356_c0_g3_i15 | 316 | 29 | 0,09177215 |
| TRINITY_DN14581_c0_g2_i3 | 141 | 13 | 0,09219858 |
| TRINITY_DN15308_c0_g3_i1 | 170 | 16 | 0,09411765 |
| TRINITY_DN16484_c1_g1_i5 | 137 | 13 | 0,09489051 |
| TRINITY_DN13322_c0_g2_i1 | 126 | 12 | 0,0952381 |
| TRINITY_DN9934_c0_g1_i6 | 125 | 12 | 0,096 |
| TRINITY_DN23104_c0_g1_i1 | 103 | 10 | 0,09708738 |
| TRINITY_DN14365_c0_g5_i2 | 163 | 16 | 0,09815951 |
| TRINITY_DN14540_c0_g2_i1 | 233 | 23 | 0,09871245 |
| TRINITY_DN16923_c0_g1_i1 | 100 | 10 | 0,1 |

**A.2. Comparison: 72 h embryo *vs* male**

**A.2.1. Transcripts over represented in embryos**

| **Transcript ID** | **Male** | **Embryo** | **FC** |
| --- | --- | --- | --- |
| TRINITY_DN12575_c0_g1_i1 | 10 | 23838 | 2383,8 |
| TRINITY_DN12527_c0_g1_i1 | 14 | 18302 | 1307,28571 |
| TRINITY_DN10186_c0_g1_i4 | 14 | 7463 | 533,071429 |
| TRINITY_DN12527_c0_g1_i2 | 15 | 7161 | 477,4 |
| TRINITY_DN16414_c0_g1_i2 | 13 | 3806 | 292,769231 |
| TRINITY_DN10167_c1_g2_i1 | 10 | 2750 | 275 |
| TRINITY_DN14198_c3_g11_i1 | 21 | 5225 | 248,809524 |
| TRINITY_DN16414_c0_g1_i1 | 15 | 3065 | 204,333333 |
| TRINITY_DN10167_c1_g1_i1 | 26 | 4648 | 178,769231 |
| TRINITY_DN11245_c0_g1_i1 | 11 | 1962 | 178,363636 |
| TRINITY_DN12288_c0_g1_i2 | 10 | 1596 | 159,6 |
| TRINITY_DN14198_c3_g9_i1 | 10 | 1596 | 159,6 |
| TRINITY_DN13678_c0_g1_i1 | 114 | 14405 | 126,359649 |
| TRINITY_DN11877_c0_g3_i1 | 44 | 5380 | 122,272727 |
| TRINITY_DN17694_c1_g3_i1 | 10 | 1015 | 101,5 |
| TRINITY_DN10167_c1_g3_i1 | 10 | 987 | 98,7 |
| TRINITY_DN16897_c1_g8_i1 | 10 | 957 | 95,7 |
| TRINITY_DN17291_c0_g3_i3 | 15 | 1432 | 95,4666667 |
| TRINITY_DN17054_c0_g1_i1 | 11 | 1000 | 90,9090909 |
| TRINITY_DN12954_c0_g1_i1 | 57 | 5118 | 89,7894737 |
| TRINITY_DN16566_c0_g2_i1 | 18 | 1586 | 88,1111111 |
| TRINITY_DN10357_c0_g1_i1 | 31 | 2643 | 85,2580645 |
| TRINITY_DN10198_c0_g3_i1 | 20 | 1658 | 82,9 |
| TRINITY_DN12882_c0_g6_i6 | 30 | 2393 | 79,7666667 |
| TRINITY_DN10186_c0_g1_i5 | 19 | 1492 | 78,5263158 |
| TRINITY_DN12006_c0_g1_i4 | 12 | 855 | 71,25 |
| TRINITY_DN14105_c0_g2_i3 | 17 | 1185 | 69,7058824 |
| TRINITY_DN12504_c0_g1_i1 | 20 | 1351 | 67,55 |
| TRINITY_DN14538_c0_g2_i6 | 10 | 667 | 66,7 |
| TRINITY_DN17593_c0_g5_i1 | 21 | 1359 | 64,7142857 |
| TRINITY_DN13225_c0_g1_i2 | 23 | 1482 | 64,4347826 |
| TRINITY_DN13129_c0_g1_i1 | 23 | 1445 | 62,826087 |
| TRINITY_DN14198_c1_g1_i3 | 20 | 1194 | 59,7 |
| TRINITY_DN12780_c0_g2_i2 | 14 | 826 | 59 |
| TRINITY_DN17721_c1_g2_i2 | 18 | 1058 | 58,7777778 |
| TRINITY_DN14730_c0_g3_i1 | 12 | 668 | 55,6666667 |
| TRINITY_DN17141_c1_g2_i1 | 57 | 3091 | 54,2280702 |
| TRINITY_DN15074_c0_g9_i1 | 41 | 2195 | 53,5365854 |
| TRINITY_DN17348_c0_g4_i3 | 23 | 1223 | 53,173913 |
| TRINITY_DN10112_c0_g1_i1 | 14 | 744 | 53,1428571 |
| TRINITY_DN17606_c0_g5_i1 | 12 | 624 | 52 |
| TRINITY_DN17744_c1_g1_i1 | 13 | 674 | 51,8461538 |
| TRINITY_DN16583_c0_g1_i1 | 11 | 568 | 51,6363636 |
| TRINITY_DN17304_c0_g1_i1 | 57 | 2942 | 51,6140351 |
| TRINITY_DN13637_c0_g1_i2 | 30 | 1456 | 48,5333333 |
| TRINITY_DN9211_c0_g3_i1 | 10 | 483 | 48,3 |
| TRINITY_DN16314_c0_g1_i1 | 13 | 567 | 43,6153846 |
| TRINITY_DN2750_c0_g1_i1 | 13 | 562 | 43,2307692 |
| TRINITY_DN14466_c0_g1_i1 | 19 | 810 | 42,6315789 |
| TRINITY_DN15472_c0_g2_i1 | 25 | 1034 | 41,36 |
| TRINITY_DN15472_c0_g6_i1 | 49 | 2004 | 40,8979592 |
| TRINITY_DN17691_c2_g4_i2 | 19 | 742 | 39,0526316 |
| TRINITY_DN15806_c9_g4_i2 | 23 | 897 | 39 |
| TRINITY_DN16247_c1_g2_i1 | 13 | 503 | 38,6923077 |
| TRINITY_DN14392_c0_g2_i7 | 11 | 419 | 38,0909091 |
| TRINITY_DN15700_c0_g3_i4 | 19 | 718 | 37,7894737 |
| TRINITY_DN17640_c0_g1_i1 | 13 | 490 | 37,6923077 |
| TRINITY_DN17733_c1_g1_i2 | 72 | 2696 | 37,4444444 |
| TRINITY_DN4968_c0_g1_i1 | 20 | 746 | 37,3 |
| TRINITY_DN17264_c0_g1_i4 | 63 | 2329 | 36,968254 |
| TRINITY_DN16390_c0_g1_i1 | 13 | 480 | 36,9230769 |
| TRINITY_DN17671_c5_g14_i1 | 33 | 1207 | 36,5757576 |
| TRINITY_DN5005_c0_g2_i1 | 91 | 3328 | 36,5714286 |
| TRINITY_DN17706_c1_g17_i2 | 13 | 474 | 36,4615385 |
| TRINITY_DN8024_c0_g1_i1 | 19 | 692 | 36,4210526 |
| TRINITY_DN17040_c0_g1_i1 | 33 | 1188 | 36 |
| TRINITY_DN16427_c1_g3_i4 | 12 | 432 | 36 |
| TRINITY_DN58_c0_g1_i1 | 11 | 394 | 35,8181818 |
| TRINITY_DN17119_c1_g1_i1 | 10 | 355 | 35,5 |
| TRINITY_DN16639_c0_g5_i1 | 13 | 461 | 35,4615385 |
| TRINITY_DN14784_c0_g3_i1 | 23 | 814 | 35,3913043 |
| TRINITY_DN17085_c0_g1_i1 | 11 | 388 | 35,2727273 |
| TRINITY_DN15074_c0_g10_i1 | 33 | 1145 | 34,6969697 |
| TRINITY_DN17235_c0_g9_i2 | 11 | 381 | 34,6363636 |
| TRINITY_DN17369_c0_g14_i4 | 23 | 794 | 34,5217391 |
| TRINITY_DN17475_c0_g3_i2 | 22 | 756 | 34,3636364 |
| TRINITY_DN17054_c0_g5_i1 | 27 | 924 | 34,2222222 |
| TRINITY_DN12667_c0_g2_i1 | 394 | 13286 | 33,7208122 |
| TRINITY_DN16810_c0_g1_i7 | 10 | 336 | 33,6 |
| TRINITY_DN17299_c0_g3_i6 | 21 | 686 | 32,6666667 |
| TRINITY_DN15496_c0_g1_i4 | 12 | 392 | 32,6666667 |
| TRINITY_DN16802_c0_g2_i2 | 12 | 391 | 32,5833333 |
| TRINITY_DN15264_c0_g2_i1 | 22 | 702 | 31,9090909 |
| TRINITY_DN17642_c0_g1_i1 | 17 | 539 | 31,7058824 |
| TRINITY_DN17683_c0_g1_i3 | 13 | 411 | 31,6153846 |
| TRINITY_DN13940_c0_g1_i3 | 35 | 1098 | 31,3714286 |
| TRINITY_DN16187_c1_g8_i2 | 39 | 1182 | 30,3076923 |
| TRINITY_DN6607_c0_g1_i1 | 10 | 302 | 30,2 |
| TRINITY_DN17506_c0_g4_i1 | 41 | 1229 | 29,9756098 |
| TRINITY_DN17587_c1_g2_i1 | 27 | 807 | 29,8888889 |
| TRINITY_DN13630_c0_g2_i3 | 13 | 371 | 28,5384615 |
| TRINITY_DN13242_c0_g1_i1 | 10 | 283 | 28,3 |
| TRINITY_DN17566_c0_g3_i1 | 17 | 481 | 28,2941176 |
| TRINITY_DN14806_c0_g1_i3 | 21 | 593 | 28,2380952 |
| TRINITY_DN14537_c0_g1_i1 | 17 | 480 | 28,2352941 |
| TRINITY_DN12862_c0_g1_i2 | 14 | 395 | 28,2142857 |
| TRINITY_DN15806_c9_g8_i1 | 17 | 475 | 27,9411765 |
| TRINITY_DN16225_c0_g3_i3 | 10 | 279 | 27,9 |
| TRINITY_DN14806_c0_g1_i1 | 30 | 835 | 27,8333333 |
| TRINITY_DN14198_c1_g1_i2 | 70 | 1947 | 27,8142857 |
| TRINITY_DN11563_c0_g2_i2 | 76 | 2110 | 27,7631579 |
| TRINITY_DN17709_c0_g2_i1 | 11 | 305 | 27,7272727 |
| TRINITY_DN16508_c1_g1_i1 | 11 | 303 | 27,5454545 |
| TRINITY_DN15859_c0_g2_i4 | 10 | 275 | 27,5 |
| TRINITY_DN8717_c0_g5_i1 | 60 | 1647 | 27,45 |
| TRINITY_DN12957_c0_g2_i1 | 13 | 355 | 27,3076923 |
| TRINITY_DN15777_c0_g1_i5 | 18 | 491 | 27,2777778 |
| TRINITY_DN15712_c0_g1_i1 | 63 | 1715 | 27,2222222 |
| TRINITY_DN16022_c0_g1_i3 | 32 | 863 | 26,96875 |
| TRINITY_DN13055_c0_g1_i4 | 84 | 2258 | 26,8809524 |
| TRINITY_DN15700_c0_g2_i1 | 16 | 429 | 26,8125 |
| TRINITY_DN11870_c0_g3_i1 | 44 | 1176 | 26,7272727 |
| TRINITY_DN10952_c0_g1_i1 | 27 | 720 | 26,6666667 |
| TRINITY_DN10537_c0_g2_i1 | 10 | 266 | 26,6 |
| TRINITY_DN17175_c0_g1_i1 | 12 | 319 | 26,5833333 |
| TRINITY_DN13647_c0_g1_i5 | 65 | 1727 | 26,5692308 |
| TRINITY_DN14859_c0_g3_i4 | 10 | 264 | 26,4 |
| TRINITY_DN10659_c0_g1_i2 | 40 | 1050 | 26,25 |
| TRINITY_DN12197_c0_g1_i1 | 36 | 935 | 25,9722222 |
| TRINITY_DN14773_c0_g1_i3 | 10 | 259 | 25,9 |
| TRINITY_DN17709_c1_g8_i1 | 14 | 360 | 25,7142857 |
| TRINITY_DN17691_c2_g2_i3 | 38 | 969 | 25,5 |
| TRINITY_DN17067_c0_g3_i5 | 12 | 305 | 25,4166667 |
| TRINITY_DN16255_c0_g2_i2 | 28 | 711 | 25,3928571 |
| TRINITY_DN16436_c0_g3_i1 | 14 | 355 | 25,3571429 |
| TRINITY_DN15631_c0_g1_i1 | 22 | 557 | 25,3181818 |
| TRINITY_DN17329_c2_g1_i4 | 14 | 354 | 25,2857143 |
| TRINITY_DN16653_c3_g4_i4 | 29 | 731 | 25,2068966 |
| TRINITY_DN29004_c0_g1_i1 | 17 | 427 | 25,1176471 |
| TRINITY_DN16243_c0_g2_i1 | 17 | 427 | 25,1176471 |
| TRINITY_DN16094_c0_g2_i4 | 22 | 550 | 25 |
| TRINITY_DN12612_c1_g1_i2 | 10 | 249 | 24,9 |
| TRINITY_DN11707_c0_g2_i3 | 19 | 472 | 24,8421053 |
| TRINITY_DN15606_c0_g1_i1 | 16 | 396 | 24,75 |
| TRINITY_DN12145_c0_g1_i1 | 103 | 2544 | 24,6990291 |
| TRINITY_DN17367_c0_g4_i1 | 42 | 1033 | 24,5952381 |
| TRINITY_DN16676_c0_g2_i3 | 20 | 491 | 24,55 |
| TRINITY_DN13714_c0_g5_i1 | 32 | 783 | 24,46875 |
| TRINITY_DN14933_c0_g1_i2 | 55 | 1342 | 24,4 |
| TRINITY_DN12987_c0_g1_i2 | 138 | 3344 | 24,2318841 |
| TRINITY_DN17614_c0_g11_i1 | 11 | 266 | 24,1818182 |
| TRINITY_DN15109_c0_g3_i5 | 14 | 338 | 24,1428571 |
| TRINITY_DN14400_c0_g2_i10 | 10 | 241 | 24,1 |
| TRINITY_DN11829_c0_g1_i3 | 102 | 2440 | 23,9215686 |
| TRINITY_DN17442_c0_g2_i2 | 17 | 405 | 23,8235294 |
| TRINITY_DN17465_c0_g1_i1 | 23 | 544 | 23,6521739 |
| TRINITY_DN11053_c0_g1_i6 | 11 | 260 | 23,6363636 |
| TRINITY_DN14872_c0_g1_i2 | 20 | 470 | 23,5 |
| TRINITY_DN17673_c0_g10_i1 | 12 | 282 | 23,5 |
| TRINITY_DN17263_c0_g1_i2 | 19 | 443 | 23,3157895 |
| TRINITY_DN12738_c0_g2_i1 | 80 | 1865 | 23,3125 |
| TRINITY_DN7011_c0_g1_i1 | 12 | 279 | 23,25 |
| TRINITY_DN12607_c0_g4_i3 | 22 | 510 | 23,1818182 |
| TRINITY_DN14794_c0_g3_i1 | 11 | 254 | 23,0909091 |
| TRINITY_DN17469_c0_g1_i1 | 15 | 346 | 23,0666667 |
| TRINITY_DN16998_c0_g2_i7 | 18 | 412 | 22,8888889 |
| TRINITY_DN17460_c0_g1_i1 | 34 | 777 | 22,8529412 |
| TRINITY_DN14957_c0_g4_i1 | 10 | 228 | 22,8 |
| TRINITY_DN9816_c0_g1_i1 | 21 | 476 | 22,6666667 |
| TRINITY_DN13373_c0_g1_i8 | 20 | 453 | 22,65 |
| TRINITY_DN17670_c0_g1_i2 | 29 | 656 | 22,6206897 |
| TRINITY_DN14806_c0_g1_i4 | 15 | 339 | 22,6 |
| TRINITY_DN17024_c0_g2_i1 | 12 | 269 | 22,4166667 |
| TRINITY_DN15149_c0_g1_i6 | 11 | 246 | 22,3636364 |
| TRINITY_DN16363_c1_g2_i5 | 10 | 223 | 22,3 |
| TRINITY_DN17401_c0_g7_i1 | 14 | 311 | 22,2142857 |
| TRINITY_DN13897_c0_g9_i1 | 20 | 441 | 22,05 |
| TRINITY_DN13821_c0_g1_i1 | 28 | 617 | 22,0357143 |
| TRINITY_DN17494_c0_g1_i5 | 14 | 308 | 22 |
| TRINITY_DN11563_c0_g2_i1 | 170 | 3735 | 21,9705882 |
| TRINITY_DN31041_c0_g1_i1 | 14 | 304 | 21,7142857 |
| TRINITY_DN13508_c0_g5_i2 | 11 | 238 | 21,6363636 |
| TRINITY_DN17415_c0_g1_i2 | 63 | 1359 | 21,5714286 |
| TRINITY_DN17607_c1_g3_i3 | 11 | 237 | 21,5454545 |
| TRINITY_DN16936_c0_g17_i2 | 19 | 408 | 21,4736842 |
| TRINITY_DN16518_c0_g3_i2 | 13 | 279 | 21,4615385 |
| TRINITY_DN13217_c1_g5_i2 | 14 | 297 | 21,2142857 |
| TRINITY_DN7905_c0_g1_i2 | 11 | 231 | 21 |
| TRINITY_DN6049_c0_g1_i1 | 10 | 210 | 21 |
| TRINITY_DN17299_c0_g3_i1 | 20 | 418 | 20,9 |
| TRINITY_DN17626_c0_g1_i1 | 24 | 501 | 20,875 |
| TRINITY_DN17597_c2_g2_i4 | 25 | 521 | 20,84 |
| TRINITY_DN17060_c1_g7_i2 | 91 | 1896 | 20,8351648 |
| TRINITY_DN10558_c0_g1_i3 | 93 | 1935 | 20,8064516 |
| TRINITY_DN13926_c0_g1_i1 | 19 | 395 | 20,7894737 |
| TRINITY_DN17730_c0_g1_i8 | 17 | 353 | 20,7647059 |
| TRINITY_DN12289_c0_g1_i1 | 10 | 207 | 20,7 |
| TRINITY_DN16591_c0_g1_i1 | 23 | 476 | 20,6956522 |
| TRINITY_DN15949_c1_g4_i3 | 13 | 268 | 20,6153846 |
| TRINITY_DN20047_c0_g1_i1 | 13 | 268 | 20,6153846 |
| TRINITY_DN12052_c0_g1_i2 | 20 | 412 | 20,6 |
| TRINITY_DN11363_c0_g2_i1 | 17 | 350 | 20,5882353 |
| TRINITY_DN12034_c0_g1_i2 | 14 | 288 | 20,5714286 |
| TRINITY_DN14276_c0_g1_i2 | 34 | 696 | 20,4705882 |
| TRINITY_DN17676_c2_g1_i3 | 13 | 266 | 20,4615385 |
| TRINITY_DN17175_c0_g1_i6 | 10 | 204 | 20,4 |
| TRINITY_DN14198_c2_g1_i1 | 284 | 5781 | 20,3556338 |
| TRINITY_DN5778_c0_g1_i1 | 30 | 610 | 20,3333333 |
| TRINITY_DN14543_c0_g1_i1 | 32 | 649 | 20,28125 |
| TRINITY_DN17206_c0_g2_i2 | 15 | 304 | 20,2666667 |
| TRINITY_DN8810_c0_g1_i2 | 859 | 17403 | 20,2596042 |
| TRINITY_DN16867_c0_g1_i2 | 172 | 3482 | 20,244186 |
| TRINITY_DN17141_c1_g1_i2 | 110 | 2223 | 20,2090909 |
| TRINITY_DN15414_c0_g4_i1 | 16 | 323 | 20,1875 |
| TRINITY_DN10903_c0_g1_i1 | 17 | 343 | 20,1764706 |
| TRINITY_DN15912_c0_g1_i8 | 12 | 242 | 20,1666667 |
| TRINITY_DN16810_c0_g1_i2 | 88 | 1772 | 20,1363636 |
| TRINITY_DN16112_c0_g2_i2 | 24 | 482 | 20,0833333 |
| TRINITY_DN7469_c0_g2_i1 | 11 | 220 | 20 |
| TRINITY_DN17433_c0_g1_i1 | 16 | 319 | 19,9375 |
| TRINITY_DN13388_c0_g1_i1 | 10 | 199 | 19,9 |
| TRINITY_DN17655_c1_g1_i1 | 66 | 1306 | 19,7878788 |
| TRINITY_DN10242_c0_g1_i1 | 14 | 277 | 19,7857143 |
| TRINITY_DN16819_c0_g3_i1 | 12 | 237 | 19,75 |
| TRINITY_DN15805_c0_g1_i1 | 749 | 14788 | 19,7436582 |
| TRINITY_DN13013_c0_g1_i1 | 10 | 197 | 19,7 |
| TRINITY_DN16863_c0_g1_i1 | 19 | 373 | 19,6315789 |
| TRINITY_DN16260_c0_g1_i1 | 44 | 862 | 19,5909091 |
| TRINITY_DN16374_c0_g16_i1 | 21 | 411 | 19,5714286 |
| TRINITY_DN12603_c0_g1_i1 | 14 | 274 | 19,5714286 |
| TRINITY_DN10198_c0_g12_i2 | 47 | 918 | 19,5319149 |
| TRINITY_DN17329_c2_g1_i3 | 19 | 370 | 19,4736842 |
| TRINITY_DN17670_c1_g1_i2 | 22 | 428 | 19,4545455 |
| TRINITY_DN16436_c0_g2_i1 | 16 | 311 | 19,4375 |
| TRINITY_DN17636_c0_g2_i1 | 30 | 583 | 19,4333333 |
| TRINITY_DN15472_c0_g6_i2 | 33 | 639 | 19,3636364 |
| TRINITY_DN4176_c0_g2_i1 | 11 | 213 | 19,3636364 |
| TRINITY_DN17352_c0_g1_i4 | 15 | 290 | 19,3333333 |
| TRINITY_DN14824_c0_g2_i2 | 15 | 289 | 19,2666667 |
| TRINITY_DN15644_c0_g2_i1 | 224 | 4313 | 19,2544643 |
| TRINITY_DN13149_c0_g4_i5 | 17 | 327 | 19,2352941 |
| TRINITY_DN16729_c0_g1_i1 | 10 | 192 | 19,2 |
| TRINITY_DN13963_c0_g1_i6 | 18 | 345 | 19,1666667 |
| TRINITY_DN10738_c0_g2_i1 | 13 | 249 | 19,1538462 |
| TRINITY_DN17536_c0_g1_i7 | 27 | 517 | 19,1481481 |
| TRINITY_DN13897_c0_g9_i3 | 32 | 611 | 19,09375 |
| TRINITY_DN13736_c0_g1_i2 | 75 | 1431 | 19,08 |
| TRINITY_DN17401_c0_g5_i2 | 16 | 303 | 18,9375 |
| TRINITY_DN14190_c0_g1_i1 | 11 | 206 | 18,7272727 |
| TRINITY_DN14658_c0_g1_i2 | 10 | 187 | 18,7 |
| TRINITY_DN16245_c0_g3_i1 | 10 | 187 | 18,7 |
| TRINITY_DN13118_c0_g4_i1 | 40 | 744 | 18,6 |
| TRINITY_DN17556_c0_g2_i1 | 34 | 632 | 18,5882353 |
| TRINITY_DN17060_c1_g7_i4 | 71 | 1314 | 18,5070423 |
| TRINITY_DN3509_c0_g1_i1 | 12 | 222 | 18,5 |
| TRINITY_DN13398_c0_g3_i4 | 11 | 203 | 18,4545455 |
| TRINITY_DN15266_c0_g1_i5 | 23 | 424 | 18,4347826 |
| TRINITY_DN17538_c0_g3_i1 | 43 | 792 | 18,4186047 |
| TRINITY_DN15178_c1_g7_i6 | 10 | 184 | 18,4 |
| TRINITY_DN8717_c0_g2_i1 | 24 | 441 | 18,375 |
| TRINITY_DN15629_c0_g2_i2 | 64 | 1174 | 18,34375 |
| TRINITY_DN12047_c0_g1_i3 | 16 | 293 | 18,3125 |
| TRINITY_DN12678_c0_g1_i5 | 15 | 274 | 18,2666667 |
| TRINITY_DN17722_c1_g3_i5 | 17 | 310 | 18,2352941 |
| TRINITY_DN14300_c0_g2_i2 | 25 | 455 | 18,2 |
| TRINITY_DN16928_c0_g1_i2 | 20 | 364 | 18,2 |
| TRINITY_DN17322_c1_g2_i4 | 22 | 400 | 18,1818182 |
| TRINITY_DN12957_c0_g2_i10 | 11 | 200 | 18,1818182 |
| TRINITY_DN8717_c0_g11_i1 | 11 | 200 | 18,1818182 |
| TRINITY_DN11899_c0_g1_i2 | 32 | 581 | 18,15625 |
| TRINITY_DN17449_c0_g2_i1 | 28 | 508 | 18,1428571 |
| TRINITY_DN17566_c0_g3_i6 | 14 | 254 | 18,1428571 |
| TRINITY_DN9763_c0_g1_i1 | 12 | 217 | 18,0833333 |
| TRINITY_DN17687_c0_g3_i6 | 20 | 361 | 18,05 |
| TRINITY_DN17742_c2_g4_i1 | 18 | 324 | 18 |
| TRINITY_DN4874_c0_g2_i2 | 16 | 288 | 18 |
| TRINITY_DN12821_c0_g1_i3 | 11 | 198 | 18 |
| TRINITY_DN10976_c0_g1_i2 | 15 | 269 | 17,9333333 |
| TRINITY_DN13469_c0_g1_i3 | 10 | 179 | 17,9 |
| TRINITY_DN11330_c0_g1_i1 | 34 | 607 | 17,8529412 |
| TRINITY_DN10688_c1_g4_i1 | 25 | 446 | 17,84 |
| TRINITY_DN17481_c0_g2_i1 | 60 | 1069 | 17,8166667 |
| TRINITY_DN14445_c1_g20_i1 | 37 | 659 | 17,8108108 |
| TRINITY_DN13881_c0_g1_i2 | 14 | 249 | 17,7857143 |
| TRINITY_DN10584_c0_g2_i3 | 85 | 1510 | 17,7647059 |
| TRINITY_DN10952_c0_g1_i2 | 109 | 1936 | 17,7614679 |
| TRINITY_DN15334_c0_g1_i1 | 15 | 266 | 17,7333333 |
| TRINITY_DN16371_c0_g2_i1 | 10 | 177 | 17,7 |
| TRINITY_DN15557_c0_g6_i3 | 61 | 1078 | 17,6721311 |
| TRINITY_DN13273_c0_g1_i1 | 10 | 176 | 17,6 |
| TRINITY_DN8285_c0_g4_i1 | 12 | 211 | 17,5833333 |
| TRINITY_DN17741_c1_g2_i1 | 12 | 211 | 17,5833333 |
| TRINITY_DN17510_c0_g1_i2 | 19 | 334 | 17,5789474 |
| TRINITY_DN16676_c0_g5_i1 | 21 | 368 | 17,5238095 |
| TRINITY_DN14851_c0_g1_i2 | 37 | 647 | 17,4864865 |
| TRINITY_DN17653_c0_g2_i1 | 23 | 400 | 17,3913043 |
| TRINITY_DN17258_c0_g1_i4 | 13 | 225 | 17,3076923 |
| TRINITY_DN15303_c0_g1_i1 | 11 | 190 | 17,2727273 |
| TRINITY_DN16660_c0_g2_i9 | 23 | 397 | 17,2608696 |
| TRINITY_DN14153_c0_g1_i2 | 20 | 345 | 17,25 |
| TRINITY_DN17014_c0_g3_i6 | 22 | 379 | 17,2272727 |
| TRINITY_DN14422_c0_g1_i4 | 22 | 378 | 17,1818182 |
| TRINITY_DN12096_c0_g5_i1 | 11 | 189 | 17,1818182 |
| TRINITY_DN16245_c0_g2_i1 | 19 | 326 | 17,1578947 |
| TRINITY_DN17596_c0_g4_i1 | 12 | 205 | 17,0833333 |
| TRINITY_DN10697_c0_g2_i1 | 13 | 222 | 17,0769231 |
| TRINITY_DN15091_c0_g1_i5 | 29 | 493 | 17 |
| TRINITY_DN17145_c0_g3_i7 | 16 | 272 | 17 |
| TRINITY_DN17494_c0_g1_i3 | 15 | 255 | 17 |
| TRINITY_DN14489_c0_g1_i6 | 11 | 187 | 17 |
| TRINITY_DN17690_c0_g10_i1 | 10 | 170 | 17 |
| TRINITY_DN16960_c0_g1_i2 | 10 | 170 | 17 |
| TRINITY_DN15806_c9_g14_i1 | 10 | 169 | 16,9 |
| TRINITY_DN16479_c0_g2_i3 | 12 | 202 | 16,8333333 |
| TRINITY_DN8602_c0_g1_i1 | 16 | 269 | 16,8125 |
| TRINITY_DN8556_c0_g1_i1 | 13 | 217 | 16,6923077 |
| TRINITY_DN17543_c1_g2_i1 | 25 | 417 | 16,68 |
| TRINITY_DN12733_c0_g1_i1 | 117 | 1948 | 16,6495726 |
| TRINITY_DN16175_c0_g1_i1 | 41 | 681 | 16,6097561 |
| TRINITY_DN16936_c0_g10_i2 | 31 | 514 | 16,5806452 |
| TRINITY_DN10591_c0_g1_i3 | 15 | 248 | 16,5333333 |
| TRINITY_DN11922_c0_g2_i1 | 12 | 198 | 16,5 |
| TRINITY_DN14412_c0_g3_i3 | 12 | 198 | 16,5 |
| TRINITY_DN17592_c0_g1_i1 | 38 | 626 | 16,4736842 |
| TRINITY_DN17621_c0_g1_i1 | 30 | 494 | 16,4666667 |
| TRINITY_DN13372_c0_g1_i1 | 47 | 771 | 16,4042553 |
| TRINITY_DN12039_c0_g1_i2 | 26 | 426 | 16,3846154 |
| TRINITY_DN14504_c0_g6_i3 | 18 | 293 | 16,2777778 |
| TRINITY_DN7868_c0_g1_i1 | 24 | 390 | 16,25 |
| TRINITY_DN14337_c0_g1_i2 | 12 | 195 | 16,25 |
| TRINITY_DN16835_c0_g7_i1 | 10 | 162 | 16,2 |
| TRINITY_DN16313_c0_g1_i2 | 21 | 339 | 16,1428571 |
| TRINITY_DN17713_c0_g3_i5 | 15 | 241 | 16,0666667 |
| TRINITY_DN16641_c0_g1_i1 | 19 | 305 | 16,0526316 |
| TRINITY_DN17518_c0_g1_i4 | 41 | 654 | 15,9512195 |
| TRINITY_DN22526_c0_g1_i1 | 16 | 254 | 15,875 |
| TRINITY_DN17607_c1_g1_i1 | 14 | 222 | 15,8571429 |
| TRINITY_DN11567_c0_g1_i2 | 14 | 222 | 15,8571429 |
| TRINITY_DN9980_c0_g2_i1 | 12 | 190 | 15,8333333 |
| TRINITY_DN10688_c1_g15_i1 | 32 | 506 | 15,8125 |
| TRINITY_DN17386_c0_g3_i4 | 11 | 173 | 15,7272727 |
| TRINITY_DN17184_c0_g1_i4 | 11 | 173 | 15,7272727 |
| TRINITY_DN15753_c0_g2_i2 | 10 | 157 | 15,7 |
| TRINITY_DN17557_c0_g1_i1 | 13 | 204 | 15,6923077 |
| TRINITY_DN17663_c0_g5_i1 | 18 | 282 | 15,6666667 |
| TRINITY_DN16171_c0_g1_i4 | 71 | 1112 | 15,6619718 |
| TRINITY_DN12967_c0_g1_i5 | 11 | 172 | 15,6363636 |
| TRINITY_DN17151_c0_g1_i2 | 19 | 297 | 15,6315789 |
| TRINITY_DN16171_c0_g1_i3 | 272 | 4249 | 15,6213235 |
| TRINITY_DN17651_c0_g1_i4 | 13 | 202 | 15,5384615 |
| TRINITY_DN16668_c0_g2_i3 | 14 | 217 | 15,5 |
| TRINITY_DN17735_c0_g3_i2 | 12 | 186 | 15,5 |
| TRINITY_DN16994_c0_g2_i3 | 10 | 155 | 15,5 |
| TRINITY_DN17608_c0_g1_i6 | 16 | 247 | 15,4375 |
| TRINITY_DN14378_c1_g1_i1 | 24 | 370 | 15,4166667 |
| TRINITY_DN17142_c0_g1_i5 | 16 | 246 | 15,375 |
| TRINITY_DN13238_c0_g6_i1 | 41 | 630 | 15,3658537 |
| TRINITY_DN16484_c1_g1_i4 | 25 | 384 | 15,36 |
| TRINITY_DN9934_c0_g1_i6 | 14 | 215 | 15,3571429 |
| TRINITY_DN10160_c0_g2_i1 | 262 | 4023 | 15,3549618 |
| TRINITY_DN16831_c0_g1_i2 | 15 | 230 | 15,3333333 |
| TRINITY_DN7682_c0_g1_i1 | 12 | 184 | 15,3333333 |
| TRINITY_DN6334_c0_g1_i1 | 16 | 245 | 15,3125 |
| TRINITY_DN16450_c2_g3_i1 | 13 | 199 | 15,3076923 |
| TRINITY_DN8838_c0_g1_i1 | 10 | 153 | 15,3 |
| TRINITY_DN13485_c0_g1_i1 | 30 | 456 | 15,2 |
| TRINITY_DN17170_c0_g2_i1 | 10 | 152 | 15,2 |
| TRINITY_DN15493_c0_g4_i1 | 16 | 242 | 15,125 |
| TRINITY_DN10655_c0_g2_i4 | 12 | 181 | 15,0833333 |
| TRINITY_DN13520_c0_g1_i1 | 12 | 181 | 15,0833333 |
| TRINITY_DN14536_c0_g1_i5 | 11 | 165 | 15 |
| TRINITY_DN15414_c0_g3_i1 | 10 | 150 | 15 |
| TRINITY_DN24136_c0_g1_i1 | 10 | 150 | 15 |
| TRINITY_DN15417_c0_g1_i5 | 43 | 643 | 14,9534884 |
| TRINITY_DN3738_c0_g2_i1 | 17 | 254 | 14,9411765 |
| TRINITY_DN16482_c2_g3_i1 | 58 | 866 | 14,9310345 |
| TRINITY_DN15354_c1_g2_i3 | 24 | 358 | 14,9166667 |
| TRINITY_DN13809_c0_g3_i1 | 23 | 343 | 14,9130435 |
| TRINITY_DN16480_c0_g3_i3 | 22 | 328 | 14,9090909 |
| TRINITY_DN16070_c0_g1_i5 | 16 | 238 | 14,875 |
| TRINITY_DN32097_c0_g1_i1 | 13 | 193 | 14,8461538 |
| TRINITY_DN17642_c0_g1_i6 | 17 | 252 | 14,8235294 |
| TRINITY_DN11173_c0_g1_i1 | 11 | 163 | 14,8181818 |
| TRINITY_DN8060_c0_g1_i1 | 16 | 237 | 14,8125 |
| TRINITY_DN13737_c0_g1_i2 | 15 | 222 | 14,8 |
| TRINITY_DN14540_c0_g1_i2 | 15 | 222 | 14,8 |
| TRINITY_DN16974_c0_g1_i1 | 10 | 148 | 14,8 |
| TRINITY_DN13863_c0_g1_i1 | 13 | 192 | 14,7692308 |
| TRINITY_DN12408_c0_g1_i4 | 13 | 192 | 14,7692308 |
| TRINITY_DN16604_c0_g1_i3 | 16 | 236 | 14,75 |
| TRINITY_DN15507_c0_g1_i2 | 12 | 177 | 14,75 |
| TRINITY_DN12714_c0_g1_i4 | 11 | 162 | 14,7272727 |
| TRINITY_DN17251_c0_g2_i2 | 24 | 353 | 14,7083333 |
| TRINITY_DN16239_c0_g3_i2 | 10 | 147 | 14,7 |
| TRINITY_DN12410_c0_g3_i2 | 16 | 235 | 14,6875 |
| TRINITY_DN16718_c1_g1_i2 | 19 | 279 | 14,6842105 |
| TRINITY_DN15227_c0_g1_i4 | 25 | 367 | 14,68 |
| TRINITY_DN10774_c0_g5_i1 | 21 | 308 | 14,6666667 |
| TRINITY_DN15252_c0_g3_i2 | 15 | 220 | 14,6666667 |
| TRINITY_DN16892_c0_g1_i1 | 32 | 469 | 14,65625 |
| TRINITY_DN16857_c0_g1_i2 | 11 | 161 | 14,6363636 |
| TRINITY_DN18104_c0_g1_i1 | 11 | 161 | 14,6363636 |
| TRINITY_DN15046_c0_g1_i3 | 11 | 161 | 14,6363636 |
| TRINITY_DN14920_c0_g1_i2 | 38 | 555 | 14,6052632 |
| TRINITY_DN12538_c0_g5_i1 | 19 | 277 | 14,5789474 |
| TRINITY_DN16708_c1_g2_i7 | 14 | 204 | 14,5714286 |
| TRINITY_DN15648_c0_g1_i1 | 74 | 1078 | 14,5675676 |
| TRINITY_DN8687_c0_g3_i1 | 11 | 160 | 14,5454545 |
| TRINITY_DN25024_c0_g1_i1 | 11 | 160 | 14,5454545 |
| TRINITY_DN10875_c1_g1_i4 | 13 | 189 | 14,5384615 |
| TRINITY_DN15240_c0_g2_i6 | 13 | 189 | 14,5384615 |
| TRINITY_DN16634_c0_g2_i2 | 12 | 174 | 14,5 |
| TRINITY_DN12689_c0_g1_i1 | 10 | 145 | 14,5 |
| TRINITY_DN11526_c1_g1_i1 | 10 | 145 | 14,5 |
| TRINITY_DN15586_c0_g2_i2 | 25 | 362 | 14,48 |
| TRINITY_DN4251_c0_g1_i1 | 19 | 275 | 14,4736842 |
| TRINITY_DN17208_c0_g1_i5 | 30 | 434 | 14,4666667 |
| TRINITY_DN492_c0_g1_i1 | 15 | 217 | 14,4666667 |
| TRINITY_DN17556_c0_g1_i3 | 11 | 159 | 14,4545455 |
| TRINITY_DN17076_c1_g1_i3 | 18 | 260 | 14,4444444 |
| TRINITY_DN17596_c0_g3_i1 | 17 | 245 | 14,4117647 |
| TRINITY_DN16398_c0_g3_i1 | 10 | 144 | 14,4 |
| TRINITY_DN16429_c0_g1_i1 | 36 | 518 | 14,3888889 |
| TRINITY_DN12300_c0_g1_i2 | 13 | 187 | 14,3846154 |
| TRINITY_DN17253_c0_g3_i1 | 36 | 516 | 14,3333333 |
| TRINITY_DN14190_c0_g2_i10 | 100 | 1433 | 14,33 |
| TRINITY_DN17186_c0_g2_i1 | 22 | 315 | 14,3181818 |
| TRINITY_DN13723_c1_g2_i1 | 11 | 157 | 14,2727273 |
| TRINITY_DN13811_c0_g1_i1 | 11 | 157 | 14,2727273 |
| TRINITY_DN15240_c0_g2_i2 | 17 | 242 | 14,2352941 |
| TRINITY_DN17156_c0_g4_i2 | 41 | 583 | 14,2195122 |
| TRINITY_DN12774_c0_g2_i2 | 23 | 327 | 14,2173913 |
| TRINITY_DN15815_c0_g1_i14 | 15 | 213 | 14,2 |
| TRINITY_DN14197_c0_g1_i1 | 33 | 468 | 14,1818182 |
| TRINITY_DN16917_c0_g1_i3 | 11 | 156 | 14,1818182 |
| TRINITY_DN15813_c0_g1_i2 | 17 | 241 | 14,1764706 |
| TRINITY_DN16161_c0_g2_i5 | 60 | 850 | 14,1666667 |
| TRINITY_DN11186_c0_g1_i4 | 17 | 240 | 14,1176471 |
| TRINITY_DN16924_c0_g2_i2 | 10 | 141 | 14,1 |
| TRINITY_DN17621_c0_g1_i2 | 31 | 437 | 14,0967742 |
| TRINITY_DN14555_c0_g3_i2 | 16 | 225 | 14,0625 |
| TRINITY_DN17558_c0_g2_i3 | 20 | 281 | 14,05 |
| TRINITY_DN17687_c0_g2_i1 | 20 | 281 | 14,05 |
| TRINITY_DN16541_c0_g3_i7 | 32 | 449 | 14,03125 |
| TRINITY_DN17739_c1_g2_i1 | 35 | 491 | 14,0285714 |
| TRINITY_DN17131_c0_g3_i3 | 41 | 574 | 14 |
| TRINITY_DN11437_c0_g2_i1 | 26 | 364 | 14 |
| TRINITY_DN3365_c0_g2_i1 | 17 | 238 | 14 |
| TRINITY_DN17185_c0_g3_i1 | 14 | 196 | 14 |
| TRINITY_DN16231_c0_g2_i4 | 13 | 182 | 14 |
| TRINITY_DN3711_c0_g1_i1 | 13 | 182 | 14 |
| TRINITY_DN16462_c0_g6_i4 | 13 | 182 | 14 |
| TRINITY_DN16065_c0_g1_i3 | 11 | 154 | 14 |
| TRINITY_DN17237_c0_g1_i1 | 23 | 321 | 13,9565217 |
| TRINITY_DN16544_c0_g1_i2 | 23 | 320 | 13,9130435 |
| TRINITY_DN13029_c0_g2_i1 | 22 | 306 | 13,9090909 |
| TRINITY_DN15328_c0_g1_i4 | 11 | 153 | 13,9090909 |
| TRINITY_DN22998_c0_g1_i1 | 11 | 153 | 13,9090909 |
| TRINITY_DN8549_c0_g3_i1 | 17 | 236 | 13,8823529 |
| TRINITY_DN17481_c0_g3_i1 | 30 | 416 | 13,8666667 |
| TRINITY_DN17600_c0_g2_i3 | 30 | 416 | 13,8666667 |
| TRINITY_DN14166_c0_g8_i1 | 20 | 277 | 13,85 |
| TRINITY_DN14577_c0_g3_i2 | 47 | 650 | 13,8297872 |
| TRINITY_DN17105_c0_g10_i8 | 23 | 318 | 13,826087 |
| TRINITY_DN8901_c0_g2_i1 | 17 | 235 | 13,8235294 |
| TRINITY_DN17500_c0_g2_i4 | 10 | 138 | 13,8 |
| TRINITY_DN12672_c0_g1_i2 | 10 | 138 | 13,8 |
| TRINITY_DN16865_c0_g1_i5 | 12 | 165 | 13,75 |
| TRINITY_DN13476_c0_g1_i2 | 11 | 151 | 13,7272727 |
| TRINITY_DN10388_c0_g1_i2 | 11 | 151 | 13,7272727 |
| TRINITY_DN16077_c0_g1_i1 | 18 | 247 | 13,7222222 |
| TRINITY_DN16732_c0_g1_i1 | 17 | 233 | 13,7058824 |
| TRINITY_DN13370_c0_g3_i2 | 10 | 137 | 13,7 |
| TRINITY_DN16495_c0_g2_i5 | 10 | 137 | 13,7 |
| TRINITY_DN15609_c0_g3_i1 | 10 | 137 | 13,7 |
| TRINITY_DN14340_c2_g7_i1 | 27 | 369 | 13,6666667 |
| TRINITY_DN17149_c0_g4_i3 | 26 | 355 | 13,6538462 |
| TRINITY_DN15078_c4_g4_i1 | 17 | 232 | 13,6470588 |
| TRINITY_DN17293_c0_g1_i7 | 14 | 191 | 13,6428571 |
| TRINITY_DN8423_c0_g2_i1 | 19 | 259 | 13,6315789 |
| TRINITY_DN15535_c1_g1_i2 | 10 | 136 | 13,6 |
| TRINITY_DN16900_c0_g3_i1 | 10 | 136 | 13,6 |
| TRINITY_DN14858_c0_g1_i9 | 54 | 734 | 13,5925926 |
| TRINITY_DN13374_c0_g2_i1 | 12 | 163 | 13,5833333 |
| TRINITY_DN14662_c0_g2_i1 | 23 | 312 | 13,5652174 |
| TRINITY_DN14806_c0_g1_i6 | 80 | 1085 | 13,5625 |
| TRINITY_DN15074_c0_g6_i1 | 33 | 447 | 13,5454545 |
| TRINITY_DN13373_c0_g1_i7 | 17 | 230 | 13,5294118 |
| TRINITY_DN12682_c0_g2_i2 | 23 | 311 | 13,5217391 |
| TRINITY_DN10688_c1_g12_i1 | 38 | 513 | 13,5 |
| TRINITY_DN16074_c0_g1_i2 | 10 | 135 | 13,5 |
| TRINITY_DN13759_c0_g1_i1 | 22 | 296 | 13,4545455 |
| TRINITY_DN15745_c0_g1_i4 | 22 | 296 | 13,4545455 |
| TRINITY_DN15430_c0_g4_i1 | 11 | 148 | 13,4545455 |
| TRINITY_DN15665_c0_g2_i2 | 16 | 215 | 13,4375 |
| TRINITY_DN16483_c0_g1_i1 | 30 | 403 | 13,4333333 |
| TRINITY_DN16511_c0_g1_i2 | 12 | 161 | 13,4166667 |
| TRINITY_DN15038_c0_g1_i1 | 15 | 201 | 13,4 |
| TRINITY_DN7445_c0_g1_i1 | 10 | 134 | 13,4 |
| TRINITY_DN16813_c0_g1_i5 | 13 | 174 | 13,3846154 |
| TRINITY_DN16060_c0_g3_i10 | 13 | 174 | 13,3846154 |
| TRINITY_DN17060_c1_g3_i1 | 81 | 1084 | 13,382716 |
| TRINITY_DN16508_c1_g1_i3 | 22 | 294 | 13,3636364 |
| TRINITY_DN15902_c1_g1_i2 | 58 | 775 | 13,362069 |
| TRINITY_DN17573_c1_g1_i1 | 65 | 868 | 13,3538462 |
| TRINITY_DN17374_c0_g1_i1 | 15 | 200 | 13,3333333 |
| TRINITY_DN17401_c0_g2_i1 | 22 | 293 | 13,3181818 |
| TRINITY_DN16226_c0_g2_i2 | 22 | 293 | 13,3181818 |
| TRINITY_DN14335_c0_g1_i1 | 20 | 266 | 13,3 |
| TRINITY_DN9427_c0_g2_i3 | 10 | 133 | 13,3 |
| TRINITY_DN11462_c0_g1_i2 | 11 | 146 | 13,2727273 |
| TRINITY_DN16616_c0_g6_i1 | 103 | 1365 | 13,2524272 |
| TRINITY_DN11795_c0_g1_i2 | 21 | 278 | 13,2380952 |
| TRINITY_DN17354_c0_g1_i2 | 22 | 291 | 13,2272727 |
| TRINITY_DN17299_c0_g3_i3 | 23 | 304 | 13,2173913 |
| TRINITY_DN17691_c1_g1_i1 | 19 | 251 | 13,2105263 |
| TRINITY_DN10145_c0_g1_i1 | 15 | 198 | 13,2 |
| TRINITY_DN17002_c2_g1_i1 | 94 | 1238 | 13,1702128 |
| TRINITY_DN15282_c1_g11_i1 | 12 | 158 | 13,1666667 |
| TRINITY_DN16868_c0_g2_i1 | 37 | 487 | 13,1621622 |
| TRINITY_DN17654_c0_g1_i1 | 43 | 564 | 13,1162791 |
| TRINITY_DN16362_c0_g4_i1 | 26 | 341 | 13,1153846 |
| TRINITY_DN17198_c0_g3_i2 | 20 | 262 | 13,1 |
| TRINITY_DN17675_c0_g3_i4 | 11 | 144 | 13,0909091 |
| TRINITY_DN8468_c0_g3_i1 | 12 | 157 | 13,0833333 |
| TRINITY_DN16424_c0_g1_i2 | 14 | 183 | 13,0714286 |
| TRINITY_DN14326_c0_g2_i4 | 15 | 196 | 13,0666667 |
| TRINITY_DN15186_c0_g1_i2 | 18 | 235 | 13,0555556 |
| TRINITY_DN17024_c0_g1_i3 | 26 | 339 | 13,0384615 |
| TRINITY_DN16047_c0_g1_i5 | 23 | 299 | 13 |
| TRINITY_DN15226_c0_g2_i4 | 17 | 221 | 13 |
| TRINITY_DN17185_c0_g4_i1 | 16 | 208 | 13 |
| TRINITY_DN8626_c0_g1_i3 | 11 | 143 | 13 |
| TRINITY_DN16702_c0_g1_i4 | 11 | 143 | 13 |
| TRINITY_DN9518_c0_g1_i2 | 11 | 143 | 13 |
| TRINITY_DN15078_c4_g1_i2 | 70 | 906 | 12,9428571 |
| TRINITY_DN11719_c0_g1_i2 | 17 | 220 | 12,9411765 |
| TRINITY_DN8509_c0_g1_i1 | 17 | 220 | 12,9411765 |
| TRINITY_DN6534_c0_g1_i1 | 16 | 207 | 12,9375 |
| TRINITY_DN17226_c1_g1_i4 | 12 | 155 | 12,9166667 |
| TRINITY_DN12654_c0_g1_i2 | 12 | 155 | 12,9166667 |
| TRINITY_DN16674_c0_g2_i6 | 12 | 155 | 12,9166667 |
| TRINITY_DN14437_c0_g1_i1 | 10 | 129 | 12,9 |
| TRINITY_DN13287_c0_g1_i1 | 18 | 232 | 12,8888889 |
| TRINITY_DN11912_c0_g1_i2 | 17 | 219 | 12,8823529 |
| TRINITY_DN16846_c0_g1_i1 | 25 | 322 | 12,88 |
| TRINITY_DN16282_c0_g2_i18 | 32 | 411 | 12,84375 |
| TRINITY_DN188_c0_g1_i1 | 32 | 411 | 12,84375 |
| TRINITY_DN10688_c1_g20_i1 | 28 | 359 | 12,8214286 |
| TRINITY_DN12261_c0_g3_i4 | 11 | 141 | 12,8181818 |
| TRINITY_DN13242_c0_g2_i1 | 15 | 192 | 12,8 |
| TRINITY_DN13894_c0_g1_i1 | 24 | 307 | 12,7916667 |
| TRINITY_DN14933_c0_g1_i1 | 86 | 1100 | 12,7906977 |
| TRINITY_DN10949_c0_g1_i5 | 14 | 179 | 12,7857143 |
| TRINITY_DN15667_c0_g1_i1 | 46 | 587 | 12,7608696 |
| TRINITY_DN14364_c0_g2_i1 | 12 | 153 | 12,75 |
| TRINITY_DN17256_c2_g3_i2 | 12 | 153 | 12,75 |
| TRINITY_DN17096_c1_g3_i3 | 19 | 242 | 12,7368421 |
| TRINITY_DN15376_c0_g1_i1 | 11 | 140 | 12,7272727 |
| TRINITY_DN13409_c0_g1_i1 | 201 | 2558 | 12,7263682 |
| TRINITY_DN9594_c0_g1_i1 | 14 | 178 | 12,7142857 |
| TRINITY_DN20369_c0_g1_i1 | 14 | 178 | 12,7142857 |
| TRINITY_DN12398_c0_g2_i2 | 397 | 5046 | 12,7103275 |
| TRINITY_DN6004_c0_g1_i1 | 10 | 127 | 12,7 |
| TRINITY_DN16022_c0_g1_i4 | 72 | 914 | 12,6944444 |
| TRINITY_DN10872_c0_g1_i1 | 13 | 165 | 12,6923077 |
| TRINITY_DN6469_c0_g1_i1 | 13 | 165 | 12,6923077 |
| TRINITY_DN17532_c1_g1_i7 | 25 | 317 | 12,68 |
| TRINITY_DN16484_c1_g1_i5 | 15 | 190 | 12,6666667 |
| TRINITY_DN13449_c0_g4_i6 | 15 | 190 | 12,6666667 |
| TRINITY_DN16912_c0_g4_i1 | 12 | 152 | 12,6666667 |
| TRINITY_DN16123_c0_g5_i6 | 11 | 139 | 12,6363636 |
| TRINITY_DN15480_c0_g1_i3 | 17 | 214 | 12,5882353 |
| TRINITY_DN11978_c0_g1_i1 | 17 | 214 | 12,5882353 |
| TRINITY_DN17079_c0_g1_i2 | 14 | 176 | 12,5714286 |
| TRINITY_DN12537_c0_g1_i1 | 16 | 201 | 12,5625 |
| TRINITY_DN13280_c0_g3_i3 | 16 | 201 | 12,5625 |
| TRINITY_DN17495_c0_g1_i1 | 35 | 439 | 12,5428571 |
| TRINITY_DN11707_c0_g2_i2 | 26 | 325 | 12,5 |
| TRINITY_DN12379_c0_g3_i4 | 12 | 150 | 12,5 |
| TRINITY_DN15110_c1_g1_i1 | 10 | 125 | 12,5 |
| TRINITY_DN10631_c0_g1_i1 | 10 | 125 | 12,5 |
| TRINITY_DN17526_c0_g3_i3 | 11 | 137 | 12,4545455 |
| TRINITY_DN8553_c0_g1_i1 | 11 | 137 | 12,4545455 |
| TRINITY_DN15588_c0_g3_i4 | 36 | 447 | 12,4166667 |
| TRINITY_DN15599_c0_g3_i2 | 12 | 149 | 12,4166667 |
| TRINITY_DN16383_c0_g3_i1 | 16 | 198 | 12,375 |
| TRINITY_DN15014_c0_g2_i2 | 145 | 1794 | 12,3724138 |
| TRINITY_DN16855_c0_g1_i2 | 70 | 866 | 12,3714286 |
| TRINITY_DN16676_c0_g2_i4 | 27 | 334 | 12,3703704 |
| TRINITY_DN17446_c0_g1_i1 | 11 | 136 | 12,3636364 |
| TRINITY_DN15216_c0_g1_i1 | 21 | 259 | 12,3333333 |
| TRINITY_DN3882_c0_g2_i1 | 28 | 345 | 12,3214286 |
| TRINITY_DN7343_c0_g1_i2 | 13 | 160 | 12,3076923 |
| TRINITY_DN5432_c0_g2_i1 | 27 | 332 | 12,2962963 |
| TRINITY_DN8415_c0_g1_i1 | 22 | 270 | 12,2727273 |
| TRINITY_DN13952_c0_g2_i2 | 24 | 294 | 12,25 |
| TRINITY_DN16649_c0_g2_i3 | 16 | 196 | 12,25 |
| TRINITY_DN13896_c0_g1_i2 | 13 | 159 | 12,2307692 |
| TRINITY_DN16760_c0_g8_i8 | 19 | 232 | 12,2105263 |
| TRINITY_DN6616_c0_g1_i1 | 10 | 122 | 12,2 |
| TRINITY_DN14135_c0_g1_i1 | 10 | 122 | 12,2 |
| TRINITY_DN13004_c0_g1_i1 | 21 | 256 | 12,1904762 |
| TRINITY_DN17494_c0_g1_i2 | 22 | 268 | 12,1818182 |
| TRINITY_DN14859_c0_g4_i1 | 20 | 243 | 12,15 |
| TRINITY_DN12020_c0_g10_i1 | 21 | 255 | 12,1428571 |
| TRINITY_DN17374_c0_g3_i3 | 14 | 170 | 12,1428571 |
| TRINITY_DN10136_c0_g1_i1 | 16 | 194 | 12,125 |
| TRINITY_DN16770_c0_g2_i1 | 51 | 618 | 12,1176471 |
| TRINITY_DN17419_c0_g1_i5 | 17 | 206 | 12,1176471 |
| TRINITY_DN12850_c0_g1_i3 | 19 | 230 | 12,1052632 |
| TRINITY_DN17043_c0_g2_i2 | 10 | 121 | 12,1 |
| TRINITY_DN13963_c0_g1_i7 | 24 | 290 | 12,0833333 |
| TRINITY_DN17154_c0_g1_i3 | 31 | 374 | 12,0645161 |
| TRINITY_DN66_c0_g2_i1 | 19 | 229 | 12,0526316 |
| TRINITY_DN15700_c0_g3_i3 | 47 | 565 | 12,0212766 |
| TRINITY_DN15879_c0_g1_i2 | 19 | 228 | 12 |
| TRINITY_DN24762_c0_g1_i1 | 18 | 216 | 12 |
| TRINITY_DN9749_c0_g1_i1 | 14 | 168 | 12 |
| TRINITY_DN17639_c1_g2_i9 | 13 | 156 | 12 |
| TRINITY_DN11178_c0_g2_i1 | 11 | 132 | 12 |
| TRINITY_DN15952_c0_g2_i5 | 22 | 263 | 11,9545455 |
| TRINITY_DN6273_c0_g1_i2 | 22 | 263 | 11,9545455 |
| TRINITY_DN7039_c0_g1_i1 | 20 | 239 | 11,95 |
| TRINITY_DN13608_c0_g1_i1 | 19 | 227 | 11,9473684 |
| TRINITY_DN12085_c0_g2_i9 | 14 | 167 | 11,9285714 |
| TRINITY_DN16676_c0_g2_i2 | 23 | 274 | 11,9130435 |
| TRINITY_DN10444_c0_g1_i3 | 22 | 262 | 11,9090909 |
| TRINITY_DN13521_c0_g1_i5 | 11 | 131 | 11,9090909 |
| TRINITY_DN15078_c0_g3_i1 | 20 | 238 | 11,9 |
| TRINITY_DN15852_c0_g3_i2 | 24 | 285 | 11,875 |
| TRINITY_DN16640_c0_g1_i3 | 23 | 273 | 11,8695652 |
| TRINITY_DN17137_c0_g1_i1 | 23 | 273 | 11,8695652 |
| TRINITY_DN12221_c0_g1_i4 | 15 | 178 | 11,8666667 |
| TRINITY_DN15627_c0_g1_i2 | 20 | 237 | 11,85 |
| TRINITY_DN14642_c0_g1_i2 | 11 | 130 | 11,8181818 |
| TRINITY_DN15423_c0_g4_i1 | 11 | 130 | 11,8181818 |
| TRINITY_DN16877_c0_g2_i1 | 35 | 413 | 11,8 |
| TRINITY_DN14400_c0_g6_i3 | 24 | 283 | 11,7916667 |
| TRINITY_DN16616_c0_g23_i1 | 24 | 282 | 11,75 |
| TRINITY_DN17338_c0_g4_i1 | 43 | 505 | 11,744186 |
| TRINITY_DN14157_c0_g2_i1 | 22 | 258 | 11,7272727 |
| TRINITY_DN11276_c0_g1_i1 | 11 | 129 | 11,7272727 |
| TRINITY_DN16096_c0_g4_i1 | 18 | 211 | 11,7222222 |
| TRINITY_DN11772_c0_g1_i2 | 21 | 246 | 11,7142857 |
| TRINITY_DN17567_c0_g3_i7 | 65 | 761 | 11,7076923 |
| TRINITY_DN6817_c0_g2_i1 | 17 | 199 | 11,7058824 |
| TRINITY_DN14545_c0_g1_i5 | 23 | 269 | 11,6956522 |
| TRINITY_DN17691_c2_g2_i4 | 74 | 865 | 11,6891892 |
| TRINITY_DN12448_c0_g2_i1 | 16 | 187 | 11,6875 |
| TRINITY_DN9199_c0_g1_i1 | 24 | 280 | 11,6666667 |
| TRINITY_DN16239_c0_g3_i1 | 18 | 210 | 11,6666667 |
| TRINITY_DN16344_c1_g4_i6 | 29 | 338 | 11,6551724 |
| TRINITY_DN16443_c0_g1_i1 | 22 | 256 | 11,6363636 |
| TRINITY_DN15241_c0_g6_i1 | 11 | 128 | 11,6363636 |
| TRINITY_DN13734_c0_g2_i3 | 76 | 883 | 11,6184211 |
| TRINITY_DN527_c0_g2_i1 | 18 | 209 | 11,6111111 |
| TRINITY_DN17710_c0_g1_i1 | 59 | 685 | 11,6101695 |
| TRINITY_DN17495_c0_g2_i1 | 45 | 522 | 11,6 |
| TRINITY_DN17249_c0_g3_i1 | 24 | 278 | 11,5833333 |
| TRINITY_DN8461_c0_g2_i1 | 310 | 3588 | 11,5741935 |
| TRINITY_DN14907_c0_g3_i1 | 16 | 185 | 11,5625 |
| TRINITY_DN17611_c1_g2_i1 | 34 | 393 | 11,5588235 |
| TRINITY_DN13915_c0_g2_i1 | 18 | 208 | 11,5555556 |
| TRINITY_DN13871_c0_g1_i2 | 11 | 127 | 11,5454545 |
| TRINITY_DN15927_c0_g1_i1 | 43 | 496 | 11,5348837 |
| TRINITY_DN15536_c0_g1_i1 | 51 | 587 | 11,5098039 |
| TRINITY_DN16243_c0_g1_i1 | 20 | 230 | 11,5 |
| TRINITY_DN5226_c0_g2_i1 | 12 | 138 | 11,5 |
| TRINITY_DN12538_c0_g9_i2 | 10 | 115 | 11,5 |
| TRINITY_DN13700_c0_g2_i2 | 29 | 333 | 11,4827586 |
| TRINITY_DN17723_c1_g1_i2 | 72 | 826 | 11,4722222 |
| TRINITY_DN14751_c0_g1_i4 | 17 | 195 | 11,4705882 |
| TRINITY_DN16653_c3_g10_i1 | 176 | 2018 | 11,4659091 |
| TRINITY_DN13249_c0_g1_i1 | 54 | 619 | 11,462963 |
| TRINITY_DN15606_c0_g1_i2 | 26 | 298 | 11,4615385 |
| TRINITY_DN12026_c0_g2_i3 | 13 | 149 | 11,4615385 |
| TRINITY_DN15156_c0_g1_i3 | 13 | 149 | 11,4615385 |
| TRINITY_DN13206_c0_g1_i2 | 24 | 275 | 11,4583333 |
| TRINITY_DN12798_c0_g1_i1 | 31 | 355 | 11,4516129 |
| TRINITY_DN15291_c0_g1_i2 | 18 | 206 | 11,4444444 |
| TRINITY_DN13838_c0_g1_i3 | 167 | 1908 | 11,4251497 |
| TRINITY_DN15946_c0_g1_i4 | 19 | 217 | 11,4210526 |
| TRINITY_DN17221_c0_g1_i10 | 12 | 137 | 11,4166667 |
| TRINITY_DN9210_c0_g1_i1 | 25 | 285 | 11,4 |
| TRINITY_DN11841_c0_g1_i3 | 10 | 114 | 11,4 |
| TRINITY_DN24711_c0_g1_i1 | 10 | 114 | 11,4 |
| TRINITY_DN16835_c0_g7_i2 | 13 | 148 | 11,3846154 |
| TRINITY_DN17559_c0_g1_i16 | 11 | 125 | 11,3636364 |
| TRINITY_DN16014_c2_g2_i2 | 11 | 125 | 11,3636364 |
| TRINITY_DN14993_c0_g2_i3 | 23 | 261 | 11,3478261 |
| TRINITY_DN15102_c0_g1_i7 | 12 | 136 | 11,3333333 |
| TRINITY_DN17446_c0_g1_i8 | 10 | 113 | 11,3 |
| TRINITY_DN14657_c0_g1_i3 | 10 | 113 | 11,3 |
| TRINITY_DN17608_c0_g1_i8 | 18 | 203 | 11,2777778 |
| TRINITY_DN17366_c1_g5_i1 | 29 | 327 | 11,2758621 |
| TRINITY_DN12538_c0_g9_i1 | 26 | 293 | 11,2692308 |
| TRINITY_DN17111_c0_g1_i2 | 41 | 462 | 11,2682927 |
| TRINITY_DN10568_c0_g1_i2 | 215 | 2416 | 11,2372093 |
| TRINITY_DN15076_c0_g2_i4 | 17 | 191 | 11,2352941 |
| TRINITY_DN13319_c0_g2_i8 | 42 | 471 | 11,2142857 |
| TRINITY_DN16188_c0_g1_i1 | 19 | 213 | 11,2105263 |
| TRINITY_DN13898_c0_g1_i2 | 25 | 280 | 11,2 |
| TRINITY_DN12427_c0_g1_i1 | 20 | 224 | 11,2 |
| TRINITY_DN17070_c0_g1_i1 | 15 | 168 | 11,2 |
| TRINITY_DN1624_c1_g1_i1 | 15 | 168 | 11,2 |
| TRINITY_DN9969_c0_g1_i1 | 10 | 112 | 11,2 |
| TRINITY_DN10313_c0_g1_i2 | 21 | 235 | 11,1904762 |
| TRINITY_DN8026_c0_g4_i1 | 11 | 123 | 11,1818182 |
| TRINITY_DN12680_c0_g2_i2 | 23 | 257 | 11,173913 |
| TRINITY_DN16626_c0_g2_i7 | 19 | 212 | 11,1578947 |
| TRINITY_DN16897_c0_g3_i2 | 17 | 189 | 11,1176471 |
| TRINITY_DN14803_c0_g2_i2 | 17 | 189 | 11,1176471 |
| TRINITY_DN13426_c0_g1_i5 | 23 | 255 | 11,0869565 |
| TRINITY_DN10622_c0_g5_i3 | 29 | 321 | 11,0689655 |
| TRINITY_DN8536_c0_g2_i5 | 18 | 199 | 11,0555556 |
| TRINITY_DN16739_c0_g3_i11 | 24 | 265 | 11,0416667 |
| TRINITY_DN11034_c0_g1_i1 | 31 | 342 | 11,0322581 |
| TRINITY_DN9838_c0_g3_i3 | 197 | 2168 | 11,0050761 |
| TRINITY_DN8802_c0_g1_i1 | 15 | 165 | 11 |
| TRINITY_DN14539_c0_g2_i2 | 13 | 143 | 11 |
| TRINITY_DN11223_c0_g1_i5 | 13 | 143 | 11 |
| TRINITY_DN16031_c0_g1_i1 | 13 | 143 | 11 |
| TRINITY_DN20556_c0_g1_i1 | 12 | 132 | 11 |
| TRINITY_DN16917_c0_g1_i2 | 11 | 121 | 11 |
| TRINITY_DN14017_c0_g2_i2 | 10 | 110 | 11 |
| TRINITY_DN10349_c0_g1_i1 | 10 | 110 | 11 |
| TRINITY_DN15362_c0_g2_i1 | 10 | 110 | 11 |
| TRINITY_DN13840_c0_g1_i3 | 10 | 110 | 11 |
| TRINITY_DN13474_c0_g9_i1 | 25 | 274 | 10,96 |
| TRINITY_DN7264_c0_g1_i1 | 17 | 186 | 10,9411765 |
| TRINITY_DN11605_c0_g1_i1 | 58 | 633 | 10,9137931 |
| TRINITY_DN13315_c0_g1_i3 | 11 | 120 | 10,9090909 |
| TRINITY_DN15805_c0_g2_i1 | 21 | 229 | 10,9047619 |
| TRINITY_DN12788_c1_g1_i3 | 31 | 338 | 10,9032258 |
| TRINITY_DN16188_c0_g1_i9 | 31 | 338 | 10,9032258 |
| TRINITY_DN17369_c0_g8_i1 | 18 | 196 | 10,8888889 |
| TRINITY_DN13238_c0_g1_i1 | 16 | 174 | 10,875 |
| TRINITY_DN12169_c0_g1_i3 | 135 | 1467 | 10,8666667 |
| TRINITY_DN13280_c0_g2_i1 | 15 | 163 | 10,8666667 |
| TRINITY_DN16351_c0_g1_i1 | 43 | 467 | 10,8604651 |
| TRINITY_DN16551_c0_g1_i4 | 21 | 228 | 10,8571429 |
| TRINITY_DN15873_c0_g1_i1 | 21 | 228 | 10,8571429 |
| TRINITY_DN10641_c0_g1_i4 | 14 | 152 | 10,8571429 |
| TRINITY_DN14212_c0_g3_i5 | 13 | 141 | 10,8461538 |
| TRINITY_DN16769_c0_g2_i4 | 30 | 325 | 10,8333333 |
| TRINITY_DN11075_c0_g1_i2 | 11 | 119 | 10,8181818 |
| TRINITY_DN15003_c0_g3_i1 | 27 | 292 | 10,8148148 |
| TRINITY_DN13898_c0_g2_i2 | 27 | 292 | 10,8148148 |
| TRINITY_DN14547_c0_g4_i2 | 43 | 465 | 10,8139535 |
| TRINITY_DN15241_c0_g6_i3 | 10 | 108 | 10,8 |
| TRINITY_DN12645_c0_g1_i1 | 10 | 108 | 10,8 |
| TRINITY_DN15225_c0_g3_i12 | 44 | 474 | 10,7727273 |
| TRINITY_DN17684_c0_g1_i1 | 22 | 237 | 10,7727273 |
| TRINITY_DN17278_c0_g1_i5 | 35 | 377 | 10,7714286 |
| TRINITY_DN16719_c0_g3_i3 | 34 | 366 | 10,7647059 |
| TRINITY_DN16532_c0_g1_i4 | 33 | 355 | 10,7575758 |
| TRINITY_DN1361_c0_g3_i1 | 24 | 258 | 10,75 |
| TRINITY_DN17063_c0_g2_i1 | 20 | 215 | 10,75 |
| TRINITY_DN14970_c0_g2_i1 | 66 | 709 | 10,7424242 |
| TRINITY_DN14569_c0_g1_i1 | 126 | 1353 | 10,7380952 |
| TRINITY_DN17337_c0_g1_i2 | 33 | 354 | 10,7272727 |
| TRINITY_DN17344_c1_g2_i1 | 11 | 118 | 10,7272727 |
| TRINITY_DN14362_c0_g1_i1 | 27 | 289 | 10,7037037 |
| TRINITY_DN15684_c0_g4_i3 | 20 | 214 | 10,7 |
| TRINITY_DN10432_c0_g8_i1 | 16 | 171 | 10,6875 |
| TRINITY_DN6065_c0_g1_i3 | 16 | 171 | 10,6875 |
| TRINITY_DN12855_c1_g4_i1 | 16 | 171 | 10,6875 |
| TRINITY_DN14491_c0_g1_i2 | 22 | 235 | 10,6818182 |
| TRINITY_DN14658_c0_g2_i1 | 12 | 128 | 10,6666667 |
| TRINITY_DN17031_c0_g5_i6 | 20 | 213 | 10,65 |
| TRINITY_DN16723_c0_g2_i3 | 53 | 564 | 10,6415094 |
| TRINITY_DN15283_c0_g3_i5 | 11 | 117 | 10,6363636 |
| TRINITY_DN12200_c0_g1_i2 | 16 | 170 | 10,625 |
| TRINITY_DN14555_c0_g2_i3 | 47 | 499 | 10,6170213 |
| TRINITY_DN16393_c0_g1_i2 | 49 | 520 | 10,6122449 |
| TRINITY_DN16573_c0_g1_i1 | 18 | 191 | 10,6111111 |
| TRINITY_DN10056_c0_g2_i1 | 24 | 254 | 10,5833333 |
| TRINITY_DN8717_c0_g3_i1 | 12 | 127 | 10,5833333 |
| TRINITY_DN14374_c0_g1_i1 | 12 | 127 | 10,5833333 |
| TRINITY_DN17175_c0_g1_i4 | 59 | 624 | 10,5762712 |
| TRINITY_DN16616_c0_g9_i1 | 42 | 444 | 10,5714286 |
| TRINITY_DN16188_c0_g1_i2 | 42 | 444 | 10,5714286 |
| TRINITY_DN13158_c0_g2_i4 | 28 | 296 | 10,5714286 |
| TRINITY_DN13812_c0_g2_i5 | 14 | 148 | 10,5714286 |
| TRINITY_DN8524_c0_g1_i1 | 14 | 148 | 10,5714286 |
| TRINITY_DN14300_c0_g3_i3 | 18 | 190 | 10,5555556 |
| TRINITY_DN8028_c0_g2_i2 | 13 | 137 | 10,5384615 |
| TRINITY_DN16819_c0_g4_i1 | 17 | 179 | 10,5294118 |
| TRINITY_DN14398_c0_g1_i1 | 25 | 263 | 10,52 |
| TRINITY_DN12624_c0_g2_i2 | 22 | 231 | 10,5 |
| TRINITY_DN17520_c2_g1_i4 | 14 | 147 | 10,5 |
| TRINITY_DN14856_c0_g1_i3 | 12 | 126 | 10,5 |
| TRINITY_DN15567_c0_g2_i1 | 10 | 105 | 10,5 |
| TRINITY_DN16696_c0_g1_i1 | 32 | 335 | 10,46875 |
| TRINITY_DN12725_c0_g3_i1 | 11 | 115 | 10,4545455 |
| TRINITY_DN5526_c0_g2_i1 | 11 | 115 | 10,4545455 |
| TRINITY_DN14970_c0_g1_i1 | 53 | 553 | 10,4339623 |
| TRINITY_DN17687_c0_g5_i1 | 14 | 146 | 10,4285714 |
| TRINITY_DN17722_c1_g3_i14 | 14 | 146 | 10,4285714 |
| TRINITY_DN15981_c0_g1_i1 | 45 | 469 | 10,4222222 |
| TRINITY_DN9166_c0_g1_i1 | 36 | 375 | 10,4166667 |
| TRINITY_DN15941_c0_g2_i2 | 12 | 125 | 10,4166667 |
| TRINITY_DN24620_c0_g1_i1 | 34 | 354 | 10,4117647 |
| TRINITY_DN12291_c0_g1_i1 | 17 | 177 | 10,4117647 |
| TRINITY_DN16647_c0_g1_i3 | 17 | 177 | 10,4117647 |
| TRINITY_DN17432_c0_g7_i1 | 20 | 208 | 10,4 |
| TRINITY_DN10598_c0_g1_i1 | 20 | 208 | 10,4 |
| TRINITY_DN10437_c0_g1_i4 | 20 | 208 | 10,4 |
| TRINITY_DN11726_c0_g1_i2 | 13 | 135 | 10,3846154 |
| TRINITY_DN11968_c0_g3_i1 | 85 | 882 | 10,3764706 |
| TRINITY_DN16961_c0_g2_i1 | 16 | 166 | 10,375 |
| TRINITY_DN16104_c0_g1_i2 | 11 | 114 | 10,3636364 |
| TRINITY_DN11331_c0_g1_i2 | 11 | 114 | 10,3636364 |
| TRINITY_DN12768_c0_g1_i1 | 14 | 145 | 10,3571429 |
| TRINITY_DN31711_c0_g1_i1 | 26 | 269 | 10,3461538 |
| TRINITY_DN17615_c0_g1_i1 | 63 | 651 | 10,3333333 |
| TRINITY_DN17493_c0_g5_i3 | 21 | 217 | 10,3333333 |
| TRINITY_DN14394_c0_g5_i2 | 15 | 155 | 10,3333333 |
| TRINITY_DN14166_c0_g7_i3 | 15 | 155 | 10,3333333 |
| TRINITY_DN17639_c1_g8_i3 | 12 | 124 | 10,3333333 |
| TRINITY_DN14627_c0_g5_i2 | 58 | 599 | 10,3275862 |
| TRINITY_DN16810_c0_g1_i6 | 43 | 444 | 10,3255814 |
| TRINITY_DN12450_c0_g1_i1 | 19 | 196 | 10,3157895 |
| TRINITY_DN17500_c0_g1_i6 | 26 | 268 | 10,3076923 |
| TRINITY_DN16038_c0_g1_i1 | 82 | 845 | 10,304878 |
| TRINITY_DN14484_c0_g1_i2 | 33 | 340 | 10,3030303 |
| TRINITY_DN17157_c0_g1_i7 | 52 | 535 | 10,2884615 |
| TRINITY_DN10160_c0_g1_i1 | 240 | 2466 | 10,275 |
| TRINITY_DN17625_c0_g1_i3 | 33 | 339 | 10,2727273 |
| TRINITY_DN10222_c0_g2_i1 | 11 | 113 | 10,2727273 |
| TRINITY_DN12216_c0_g2_i1 | 27 | 277 | 10,2592593 |
| TRINITY_DN17589_c0_g1_i5 | 28 | 287 | 10,25 |
| TRINITY_DN16721_c0_g1_i2 | 24 | 246 | 10,25 |
| TRINITY_DN13549_c0_g1_i1 | 20 | 205 | 10,25 |
| TRINITY_DN4483_c0_g1_i1 | 12 | 123 | 10,25 |
| TRINITY_DN8717_c0_g9_i1 | 25 | 256 | 10,24 |
| TRINITY_DN12697_c0_g2_i2 | 27 | 276 | 10,2222222 |
| TRINITY_DN14553_c0_g2_i6 | 18 | 184 | 10,2222222 |
| TRINITY_DN16634_c0_g1_i9 | 19 | 194 | 10,2105263 |
| TRINITY_DN17377_c0_g1_i5 | 72 | 735 | 10,2083333 |
| TRINITY_DN13634_c0_g2_i2 | 35 | 357 | 10,2 |
| TRINITY_DN12370_c2_g1_i1 | 10 | 102 | 10,2 |
| TRINITY_DN17658_c0_g2_i2 | 37 | 377 | 10,1891892 |
| TRINITY_DN2601_c0_g2_i1 | 16 | 163 | 10,1875 |
| TRINITY_DN17646_c0_g2_i1 | 11 | 112 | 10,1818182 |
| TRINITY_DN16129_c0_g5_i2 | 85 | 864 | 10,1647059 |
| TRINITY_DN17174_c0_g1_i5 | 38 | 386 | 10,1578947 |
| TRINITY_DN13683_c0_g1_i1 | 26 | 264 | 10,1538462 |
| TRINITY_DN16943_c0_g2_i1 | 28 | 284 | 10,1428571 |
| TRINITY_DN16356_c0_g3_i15 | 28 | 284 | 10,1428571 |
| TRINITY_DN14093_c0_g2_i2 | 14 | 142 | 10,1428571 |
| TRINITY_DN17684_c0_g1_i8 | 22 | 223 | 10,1363636 |
| TRINITY_DN14563_c0_g1_i1 | 15 | 152 | 10,1333333 |
| TRINITY_DN14043_c0_g2_i1 | 36 | 364 | 10,1111111 |
| TRINITY_DN17180_c0_g1_i5 | 30 | 303 | 10,1 |
| TRINITY_DN12854_c1_g1_i1 | 20 | 202 | 10,1 |
| TRINITY_DN17382_c0_g1_i6 | 10 | 101 | 10,1 |
| TRINITY_DN15977_c0_g1_i1 | 10 | 101 | 10,1 |
| TRINITY_DN17131_c0_g3_i1 | 22 | 222 | 10,0909091 |
| TRINITY_DN113_c0_g1_i2 | 11 | 111 | 10,0909091 |
| TRINITY_DN16700_c0_g1_i4 | 11 | 111 | 10,0909091 |
| TRINITY_DN7939_c0_g1_i1 | 12 | 121 | 10,0833333 |
| TRINITY_DN13062_c0_g2_i1 | 12 | 121 | 10,0833333 |
| TRINITY_DN16372_c0_g1_i1 | 13 | 131 | 10,0769231 |
| TRINITY_DN12434_c0_g1_i1 | 16 | 161 | 10,0625 |
| TRINITY_DN13881_c0_g1_i5 | 20 | 201 | 10,05 |
| TRINITY_DN16824_c0_g1_i1 | 28 | 281 | 10,0357143 |
| TRINITY_DN13926_c1_g1_i1 | 97 | 970 | 10 |
| TRINITY_DN8676_c0_g1_i1 | 38 | 380 | 10 |
| TRINITY_DN10834_c0_g2_i1 | 24 | 240 | 10 |
| TRINITY_DN12305_c0_g1_i1 | 21 | 210 | 10 |
| TRINITY_DN15863_c0_g9_i1 | 16 | 160 | 10 |
| TRINITY_DN16619_c0_g1_i3 | 12 | 120 | 10 |
| TRINITY_DN14184_c0_g2_i1 | 10 | 100 | 10 |
| TRINITY_DN24380_c0_g1_i1 | 10 | 100 | 10 |

**A.2. Comparison: 72 h embryo *vs* male**

**A.2.2. Transcripts over represented in males**

| **Transcript ID** | **Male** | **Embryo** | **FC(E/M)** |
| --- | --- | --- | --- |
| TRINITY_DN14141_c1_g8_i2 | 16319 | 11 | 0,00067406 |
| TRINITY_DN12470_c0_g2_i3 | 12990 | 17 | 0,0013087 |
| TRINITY_DN15181_c0_g2_i1 | 7890 | 21 | 0,0026616 |
| TRINITY_DN15181_c2_g8_i2 | 7462 | 34 | 0,00455642 |
| TRINITY_DN12498_c0_g4_i1 | 3475 | 16 | 0,00460432 |
| TRINITY_DN15733_c4_g31_i2 | 1989 | 10 | 0,00502765 |
| TRINITY_DN14062_c0_g1_i13 | 2057 | 11 | 0,00534759 |
| TRINITY_DN13560_c0_g1_i8 | 1681 | 10 | 0,00594884 |
| TRINITY_DN17502_c0_g2_i1 | 1517 | 10 | 0,00659196 |
| TRINITY_DN17714_c0_g4_i1 | 2424 | 17 | 0,0070132 |
| TRINITY_DN12498_c0_g3_i1 | 33903 | 240 | 0,00707902 |
| TRINITY_DN10350_c0_g1_i5 | 3247 | 23 | 0,00708346 |
| TRINITY_DN11027_c0_g1_i1 | 1372 | 10 | 0,00728863 |
| TRINITY_DN12629_c0_g1_i2 | 1976 | 15 | 0,00759109 |
| TRINITY_DN9325_c0_g1_i1 | 3015 | 23 | 0,00762852 |
| TRINITY_DN17414_c0_g5_i1 | 2185 | 17 | 0,00778032 |
| TRINITY_DN16930_c1_g1_i3 | 1821 | 16 | 0,00878638 |
| TRINITY_DN14497_c0_g6_i1 | 1458 | 13 | 0,00891632 |
| TRINITY_DN15181_c2_g4_i1 | 3125 | 31 | 0,00992 |
| TRINITY_DN9887_c0_g1_i2 | 4624 | 51 | 0,01102941 |
| TRINITY_DN10750_c0_g1_i2 | 2297 | 26 | 0,01131911 |
| TRINITY_DN17414_c0_g4_i2 | 959 | 11 | 0,01147028 |
| TRINITY_DN13095_c0_g2_i1 | 920 | 11 | 0,01195652 |
| TRINITY_DN14889_c1_g1_i1 | 1589 | 19 | 0,01195721 |
| TRINITY_DN14418_c0_g2_i1 | 1159 | 14 | 0,01207938 |
| TRINITY_DN13462_c0_g2_i4 | 1962 | 26 | 0,01325178 |
| TRINITY_DN12044_c0_g13_i1 | 3155 | 43 | 0,01362916 |
| TRINITY_DN16444_c0_g2_i1 | 1267 | 18 | 0,01420679 |
| TRINITY_DN13718_c0_g1_i2 | 1165 | 17 | 0,01459227 |
| TRINITY_DN17414_c0_g2_i1 | 1758 | 26 | 0,01478953 |
| TRINITY_DN17005_c0_g1_i1 | 1068 | 16 | 0,01498127 |
| TRINITY_DN17247_c0_g1_i2 | 858 | 13 | 0,01515152 |
| TRINITY_DN8975_c212_g7_i1 | 1086 | 17 | 0,01565378 |
| TRINITY_DN17456_c0_g1_i3 | 826 | 13 | 0,0157385 |
| TRINITY_DN14062_c0_g1_i9 | 1230 | 21 | 0,01707317 |
| TRINITY_DN16502_c0_g1_i2 | 1314 | 23 | 0,01750381 |
| TRINITY_DN11311_c0_g1_i2 | 21408 | 380 | 0,01775037 |
| TRINITY_DN16256_c0_g7_i1 | 10329 | 184 | 0,01781392 |
| TRINITY_DN13143_c0_g2_i3 | 2907 | 53 | 0,01823185 |
| TRINITY_DN16851_c0_g2_i1 | 857 | 16 | 0,01866978 |
| TRINITY_DN16287_c0_g1_i1 | 1636 | 31 | 0,01894866 |
| TRINITY_DN16256_c0_g13_i1 | 4991 | 95 | 0,01903426 |
| TRINITY_DN16851_c0_g3_i1 | 1554 | 30 | 0,01930502 |
| TRINITY_DN17410_c0_g10_i3 | 818 | 16 | 0,0195599 |
| TRINITY_DN14382_c0_g1_i2 | 2173 | 45 | 0,0207087 |
| TRINITY_DN17339_c0_g7_i2 | 524 | 11 | 0,02099237 |
| TRINITY_DN12498_c0_g2_i1 | 746 | 16 | 0,02144772 |
| TRINITY_DN17005_c0_g3_i1 | 1324 | 30 | 0,02265861 |
| TRINITY_DN12195_c0_g1_i1 | 1708 | 40 | 0,0234192 |
| TRINITY_DN16851_c0_g2_i2 | 1552 | 38 | 0,02448454 |
| TRINITY_DN17073_c0_g1_i2 | 919 | 23 | 0,0250272 |
| TRINITY_DN11246_c0_g1_i1 | 436 | 11 | 0,02522936 |
| TRINITY_DN14767_c0_g1_i2 | 907 | 23 | 0,02535832 |
| TRINITY_DN14438_c0_g1_i2 | 1216 | 31 | 0,02549342 |
| TRINITY_DN9904_c0_g3_i1 | 391 | 10 | 0,02557545 |
| TRINITY_DN16930_c1_g1_i4 | 547 | 14 | 0,02559415 |
| TRINITY_DN16256_c0_g11_i1 | 12811 | 333 | 0,02599329 |
| TRINITY_DN14004_c0_g1_i1 | 1057 | 28 | 0,02649007 |
| TRINITY_DN10942_c0_g2_i3 | 1281 | 34 | 0,02654176 |
| TRINITY_DN14385_c0_g3_i1 | 601 | 16 | 0,0266223 |
| TRINITY_DN8906_c0_g3_i1 | 3611 | 98 | 0,0271393 |
| TRINITY_DN10248_c0_g2_i1 | 3537 | 99 | 0,02798982 |
| TRINITY_DN14715_c0_g1_i3 | 498 | 14 | 0,02811245 |
| TRINITY_DN17728_c0_g3_i1 | 849 | 24 | 0,02826855 |
| TRINITY_DN12498_c0_g1_i1 | 15139 | 431 | 0,02846952 |
| TRINITY_DN9228_c0_g2_i2 | 3744 | 107 | 0,02857906 |
| TRINITY_DN16698_c0_g2_i2 | 1469 | 42 | 0,02859088 |
| TRINITY_DN14014_c0_g1_i1 | 515 | 15 | 0,02912621 |
| TRINITY_DN16797_c0_g1_i3 | 472 | 14 | 0,02966102 |
| TRINITY_DN13771_c0_g1_i6 | 404 | 12 | 0,02970297 |
| TRINITY_DN14178_c0_g1_i2 | 3604 | 108 | 0,0299667 |
| TRINITY_DN14445_c1_g23_i1 | 431 | 13 | 0,03016241 |
| TRINITY_DN10750_c0_g1_i1 | 357 | 11 | 0,03081232 |
| TRINITY_DN17361_c0_g6_i4 | 518 | 16 | 0,03088803 |
| TRINITY_DN16277_c0_g1_i2 | 448 | 14 | 0,03125 |
| TRINITY_DN17361_c0_g6_i6 | 543 | 17 | 0,03130755 |
| TRINITY_DN11957_c0_g2_i1 | 725 | 23 | 0,03172414 |
| TRINITY_DN5599_c0_g1_i1 | 899 | 29 | 0,03225806 |
| TRINITY_DN13095_c0_g1_i1 | 1813 | 59 | 0,03254275 |
| TRINITY_DN16256_c0_g2_i1 | 1587 | 52 | 0,03276623 |
| TRINITY_DN16864_c0_g2_i2 | 363 | 12 | 0,03305785 |
| TRINITY_DN17210_c1_g1_i3 | 1648 | 55 | 0,03337379 |
| TRINITY_DN8531_c0_g3_i1 | 650 | 22 | 0,03384615 |
| TRINITY_DN17704_c1_g1_i1 | 374 | 13 | 0,03475936 |
| TRINITY_DN10514_c0_g2_i1 | 39248 | 1373 | 0,03498267 |
| TRINITY_DN16256_c0_g5_i1 | 1171 | 41 | 0,03501281 |
| TRINITY_DN15570_c0_g1_i2 | 2795 | 98 | 0,03506261 |
| TRINITY_DN15278_c1_g3_i1 | 312 | 11 | 0,03525641 |
| TRINITY_DN15989_c0_g1_i9 | 312 | 11 | 0,03525641 |
| TRINITY_DN17637_c0_g10_i2 | 894 | 32 | 0,03579418 |
| TRINITY_DN9315_c0_g1_i1 | 387 | 14 | 0,03617571 |
| TRINITY_DN17224_c0_g3_i2 | 410 | 15 | 0,03658537 |
| TRINITY_DN15404_c0_g1_i2 | 273 | 10 | 0,03663004 |
| TRINITY_DN16027_c2_g1_i11 | 299 | 11 | 0,0367893 |
| TRINITY_DN15560_c0_g1_i1 | 298 | 11 | 0,03691275 |
| TRINITY_DN17410_c0_g5_i2 | 402 | 15 | 0,03731343 |
| TRINITY_DN17008_c1_g1_i2 | 348 | 13 | 0,03735632 |
| TRINITY_DN16050_c0_g3_i1 | 348 | 13 | 0,03735632 |
| TRINITY_DN17318_c0_g1_i1 | 1134 | 43 | 0,03791887 |
| TRINITY_DN16256_c0_g20_i1 | 12889 | 491 | 0,0380945 |
| TRINITY_DN14405_c0_g3_i1 | 312 | 12 | 0,03846154 |
| TRINITY_DN17414_c0_g13_i2 | 620 | 24 | 0,03870968 |
| TRINITY_DN17647_c1_g3_i1 | 335 | 13 | 0,03880597 |
| TRINITY_DN8791_c0_g1_i1 | 257 | 10 | 0,03891051 |
| TRINITY_DN14705_c0_g1_i2 | 280 | 11 | 0,03928571 |
| TRINITY_DN15181_c2_g20_i1 | 575 | 23 | 0,04 |
| TRINITY_DN16880_c0_g3_i5 | 348 | 14 | 0,04022989 |
| TRINITY_DN13343_c0_g2_i1 | 1134 | 46 | 0,04056437 |
| TRINITY_DN10482_c0_g1_i4 | 562 | 23 | 0,04092527 |
| TRINITY_DN16581_c0_g1_i6 | 268 | 11 | 0,04104478 |
| TRINITY_DN16952_c0_g7_i2 | 877 | 36 | 0,04104903 |
| TRINITY_DN15628_c0_g2_i2 | 243 | 10 | 0,04115226 |
| TRINITY_DN17637_c0_g8_i2 | 315 | 13 | 0,04126984 |
| TRINITY_DN5730_c0_g2_i2 | 334 | 14 | 0,04191617 |
| TRINITY_DN2247_c0_g1_i1 | 3615 | 153 | 0,04232365 |
| TRINITY_DN7413_c0_g1_i1 | 400 | 17 | 0,0425 |
| TRINITY_DN9611_c0_g1_i1 | 862 | 37 | 0,04292343 |
| TRINITY_DN13343_c0_g1_i1 | 1100 | 48 | 0,04363636 |
| TRINITY_DN15202_c0_g1_i2 | 366 | 16 | 0,04371585 |
| TRINITY_DN15854_c0_g1_i3 | 526 | 23 | 0,04372624 |
| TRINITY_DN17310_c0_g5_i4 | 274 | 12 | 0,04379562 |
| TRINITY_DN11233_c0_g1_i3 | 364 | 16 | 0,04395604 |
| TRINITY_DN10422_c0_g1_i1 | 113217 | 4991 | 0,04408349 |
| TRINITY_DN16880_c0_g3_i3 | 999 | 45 | 0,04504505 |
| TRINITY_DN14445_c1_g10_i1 | 332 | 15 | 0,04518072 |
| TRINITY_DN16273_c0_g1_i7 | 442 | 20 | 0,04524887 |
| TRINITY_DN9154_c0_g2_i3 | 309 | 14 | 0,04530744 |
| TRINITY_DN17315_c1_g8_i9 | 330 | 15 | 0,04545455 |
| TRINITY_DN10686_c0_g1_i1 | 219 | 10 | 0,0456621 |
| TRINITY_DN16568_c0_g1_i3 | 257 | 12 | 0,04669261 |
| TRINITY_DN16568_c0_g1_i8 | 212 | 10 | 0,04716981 |
| TRINITY_DN11264_c0_g1_i2 | 8976 | 427 | 0,0475713 |
| TRINITY_DN17727_c0_g2_i5 | 378 | 18 | 0,04761905 |
| TRINITY_DN6779_c0_g1_i1 | 272 | 13 | 0,04779412 |
| TRINITY_DN14455_c0_g1_i2 | 580 | 28 | 0,04827586 |
| TRINITY_DN16233_c0_g11_i2 | 207 | 10 | 0,04830918 |
| TRINITY_DN17679_c0_g4_i2 | 227 | 11 | 0,04845815 |
| TRINITY_DN17008_c1_g1_i3 | 426 | 21 | 0,04929577 |
| TRINITY_DN11817_c0_g1_i4 | 607 | 30 | 0,04942339 |
| TRINITY_DN14990_c0_g1_i1 | 202 | 10 | 0,04950495 |
| TRINITY_DN17323_c0_g1_i1 | 221 | 11 | 0,04977376 |
| TRINITY_DN14715_c0_g1_i6 | 219 | 11 | 0,05022831 |
| TRINITY_DN17315_c1_g8_i10 | 1349 | 68 | 0,05040771 |
| TRINITY_DN14637_c2_g3_i2 | 218 | 11 | 0,05045872 |
| TRINITY_DN17023_c0_g2_i1 | 198 | 10 | 0,05050505 |
| TRINITY_DN14178_c0_g1_i1 | 5167 | 261 | 0,05051287 |
| TRINITY_DN8975_c243_g1_i1 | 216 | 11 | 0,05092593 |
| TRINITY_DN12549_c1_g1_i1 | 2314 | 119 | 0,0514261 |
| TRINITY_DN14889_c1_g1_i3 | 544 | 28 | 0,05147059 |
| TRINITY_DN14767_c0_g1_i3 | 271 | 14 | 0,05166052 |
| TRINITY_DN7258_c0_g2_i1 | 329 | 17 | 0,05167173 |
| TRINITY_DN17457_c0_g4_i3 | 251 | 13 | 0,05179283 |
| TRINITY_DN16051_c0_g2_i1 | 211 | 11 | 0,0521327 |
| TRINITY_DN15489_c1_g1_i1 | 211 | 11 | 0,0521327 |
| TRINITY_DN16930_c0_g1_i3 | 611 | 32 | 0,05237316 |
| TRINITY_DN16753_c0_g1_i2 | 210 | 11 | 0,05238095 |
| TRINITY_DN15416_c0_g1_i6 | 303 | 16 | 0,05280528 |
| TRINITY_DN15349_c0_g2_i1 | 188 | 10 | 0,05319149 |
| TRINITY_DN8632_c0_g1_i1 | 356 | 19 | 0,05337079 |
| TRINITY_DN17310_c0_g3_i1 | 262 | 14 | 0,05343511 |
| TRINITY_DN17586_c0_g2_i2 | 409 | 22 | 0,05378973 |
| TRINITY_DN15705_c0_g1_i3 | 223 | 12 | 0,05381166 |
| TRINITY_DN14486_c0_g1_i2 | 571 | 31 | 0,05429072 |
| TRINITY_DN3396_c0_g1_i1 | 202 | 11 | 0,05445545 |
| TRINITY_DN11233_c0_g1_i4 | 220 | 12 | 0,05454545 |
| TRINITY_DN17617_c0_g6_i2 | 292 | 16 | 0,05479452 |
| TRINITY_DN14868_c0_g1_i4 | 1295 | 71 | 0,05482625 |
| TRINITY_DN14295_c0_g1_i5 | 273 | 15 | 0,05494505 |
| TRINITY_DN11055_c0_g1_i1 | 525 | 29 | 0,0552381 |
| TRINITY_DN13217_c1_g4_i1 | 199 | 11 | 0,05527638 |
| TRINITY_DN15425_c0_g1_i4 | 1843 | 103 | 0,05588714 |
| TRINITY_DN11556_c1_g3_i1 | 589 | 33 | 0,05602716 |
| TRINITY_DN14542_c0_g1_i2 | 460 | 26 | 0,05652174 |
| TRINITY_DN16136_c0_g1_i1 | 230 | 13 | 0,05652174 |
| TRINITY_DN12549_c0_g1_i2 | 1230 | 70 | 0,05691057 |
| TRINITY_DN11623_c0_g3_i1 | 615 | 35 | 0,05691057 |
| TRINITY_DN16779_c0_g2_i1 | 911 | 52 | 0,05708013 |
| TRINITY_DN14630_c0_g2_i6 | 175 | 10 | 0,05714286 |
| TRINITY_DN16880_c0_g1_i3 | 262 | 15 | 0,05725191 |
| TRINITY_DN11220_c0_g1_i2 | 242 | 14 | 0,05785124 |
| TRINITY_DN14074_c0_g1_i1 | 204 | 12 | 0,05882353 |
| TRINITY_DN15103_c0_g7_i1 | 204 | 12 | 0,05882353 |
| TRINITY_DN17315_c1_g8_i1 | 930 | 55 | 0,05913978 |
| TRINITY_DN15916_c0_g4_i3 | 490 | 29 | 0,05918367 |
| TRINITY_DN17339_c0_g7_i1 | 1528 | 91 | 0,05955497 |
| TRINITY_DN17007_c0_g2_i1 | 285 | 17 | 0,05964912 |
| TRINITY_DN11993_c0_g2_i4 | 217 | 13 | 0,05990783 |
| TRINITY_DN9654_c0_g1_i1 | 216 | 13 | 0,06018519 |
| TRINITY_DN14873_c0_g1_i1 | 232 | 14 | 0,06034483 |
| TRINITY_DN16779_c0_g2_i2 | 1225 | 74 | 0,06040816 |
| TRINITY_DN15745_c0_g1_i5 | 215 | 13 | 0,06046512 |
| TRINITY_DN15548_c0_g1_i3 | 328 | 20 | 0,06097561 |
| TRINITY_DN5168_c0_g1_i2 | 1686 | 103 | 0,06109134 |
| TRINITY_DN12588_c0_g2_i4 | 802 | 49 | 0,06109726 |
| TRINITY_DN17727_c0_g1_i1 | 491 | 30 | 0,0610998 |
| TRINITY_DN2413_c0_g1_i1 | 650 | 40 | 0,06153846 |
| TRINITY_DN10420_c0_g2_i1 | 260 | 16 | 0,06153846 |
| TRINITY_DN15190_c0_g1_i1 | 681 | 42 | 0,06167401 |
| TRINITY_DN16057_c0_g1_i3 | 227 | 14 | 0,06167401 |
| TRINITY_DN12566_c0_g1_i3 | 7970 | 500 | 0,06273526 |
| TRINITY_DN12895_c0_g1_i2 | 191 | 12 | 0,06282723 |
| TRINITY_DN12928_c0_g1_i3 | 302 | 19 | 0,06291391 |
| TRINITY_DN17389_c0_g8_i4 | 204 | 13 | 0,06372549 |
| TRINITY_DN17017_c0_g1_i1 | 666 | 43 | 0,06456456 |
| TRINITY_DN17586_c0_g2_i3 | 325 | 21 | 0,06461538 |
| TRINITY_DN13352_c0_g1_i1 | 354 | 23 | 0,06497175 |
| TRINITY_DN9305_c0_g1_i3 | 215 | 14 | 0,06511628 |
| TRINITY_DN16027_c2_g1_i7 | 261 | 17 | 0,0651341 |
| TRINITY_DN17576_c0_g1_i7 | 230 | 15 | 0,06521739 |
| TRINITY_DN15388_c0_g1_i14 | 521 | 34 | 0,06525912 |
| TRINITY_DN17734_c1_g7_i9 | 260 | 17 | 0,06538462 |
| TRINITY_DN17717_c0_g3_i3 | 535 | 35 | 0,06542056 |
| TRINITY_DN15550_c0_g1_i2 | 381 | 25 | 0,0656168 |
| TRINITY_DN16952_c0_g1_i2 | 668 | 44 | 0,06586826 |
| TRINITY_DN16880_c0_g1_i1 | 493 | 33 | 0,06693712 |
| TRINITY_DN14400_c0_g1_i1 | 164 | 11 | 0,06707317 |
| TRINITY_DN11556_c1_g1_i2 | 459 | 31 | 0,06753813 |
| TRINITY_DN16175_c1_g2_i2 | 428 | 29 | 0,06775701 |
| TRINITY_DN14160_c1_g4_i3 | 531 | 36 | 0,06779661 |
| TRINITY_DN15549_c0_g1_i2 | 177 | 12 | 0,06779661 |
| TRINITY_DN14438_c0_g1_i1 | 575 | 39 | 0,06782609 |
| TRINITY_DN15017_c0_g1_i1 | 501 | 34 | 0,06786427 |
| TRINITY_DN15285_c0_g1_i2 | 498 | 34 | 0,06827309 |
| TRINITY_DN16251_c1_g3_i1 | 249 | 17 | 0,06827309 |
| TRINITY_DN17273_c1_g2_i4 | 321 | 22 | 0,06853583 |
| TRINITY_DN15431_c0_g1_i4 | 175 | 12 | 0,06857143 |
| TRINITY_DN12132_c0_g1_i2 | 145 | 10 | 0,06896552 |
| TRINITY_DN10202_c0_g1_i3 | 939 | 65 | 0,06922258 |
| TRINITY_DN14062_c0_g1_i5 | 789 | 55 | 0,06970849 |
| TRINITY_DN12417_c0_g1_i1 | 645 | 45 | 0,06976744 |
| TRINITY_DN12818_c0_g6_i1 | 802 | 56 | 0,06982544 |
| TRINITY_DN11474_c0_g1_i3 | 143 | 10 | 0,06993007 |
| TRINITY_DN10750_c0_g1_i3 | 656 | 46 | 0,07012195 |
| TRINITY_DN15085_c0_g3_i2 | 898 | 63 | 0,0701559 |
| TRINITY_DN16168_c0_g6_i5 | 778 | 55 | 0,07069409 |
| TRINITY_DN17637_c0_g6_i1 | 409 | 29 | 0,07090465 |
| TRINITY_DN19137_c0_g1_i1 | 155 | 11 | 0,07096774 |
| TRINITY_DN11086_c0_g1_i2 | 324 | 23 | 0,07098765 |
| TRINITY_DN16642_c0_g1_i7 | 168 | 12 | 0,07142857 |
| TRINITY_DN10202_c0_g1_i2 | 167 | 12 | 0,07185629 |
| TRINITY_DN9463_c0_g3_i2 | 333 | 24 | 0,07207207 |
| TRINITY_DN12845_c0_g2_i4 | 138 | 10 | 0,07246377 |
| TRINITY_DN17314_c1_g1_i2 | 179 | 13 | 0,0726257 |
| TRINITY_DN17740_c0_g5_i1 | 563 | 41 | 0,07282416 |
| TRINITY_DN34439_c0_g1_i1 | 151 | 11 | 0,07284768 |
| TRINITY_DN11391_c0_g1_i2 | 1122 | 82 | 0,07308378 |
| TRINITY_DN15570_c0_g1_i1 | 7146 | 526 | 0,07360761 |
| TRINITY_DN17007_c0_g1_i3 | 190 | 14 | 0,07368421 |
| TRINITY_DN10765_c0_g3_i1 | 1488 | 110 | 0,07392473 |
| TRINITY_DN8570_c0_g2_i1 | 229 | 17 | 0,07423581 |
| TRINITY_DN12524_c0_g1_i3 | 578 | 43 | 0,07439446 |
| TRINITY_DN15268_c0_g3_i1 | 215 | 16 | 0,0744186 |
| TRINITY_DN17071_c0_g2_i1 | 174 | 13 | 0,07471264 |
| TRINITY_DN17544_c0_g1_i7 | 254 | 19 | 0,07480315 |
| TRINITY_DN9381_c0_g1_i2 | 531 | 40 | 0,07532957 |
| TRINITY_DN13010_c2_g1_i2 | 212 | 16 | 0,0754717 |
| TRINITY_DN14174_c0_g1_i4 | 264 | 20 | 0,07575758 |
| TRINITY_DN17665_c0_g3_i3 | 277 | 21 | 0,07581227 |
| TRINITY_DN13268_c0_g5_i1 | 540 | 41 | 0,07592593 |
| TRINITY_DN2037_c0_g1_i1 | 158 | 12 | 0,07594937 |
| TRINITY_DN13211_c0_g4_i3 | 394 | 30 | 0,07614213 |
| TRINITY_DN9709_c0_g2_i2 | 1046 | 80 | 0,07648184 |
| TRINITY_DN13211_c0_g4_i2 | 530 | 41 | 0,07735849 |
| TRINITY_DN16580_c0_g1_i3 | 155 | 12 | 0,07741935 |
| TRINITY_DN19393_c0_g1_i1 | 308 | 24 | 0,07792208 |
| TRINITY_DN16768_c0_g2_i2 | 217 | 17 | 0,07834101 |
| TRINITY_DN16475_c0_g2_i5 | 152 | 12 | 0,07894737 |
| TRINITY_DN17073_c0_g1_i4 | 328 | 26 | 0,07926829 |
| TRINITY_DN16712_c0_g1_i6 | 164 | 13 | 0,07926829 |
| TRINITY_DN14619_c0_g1_i1 | 151 | 12 | 0,0794702 |
| TRINITY_DN15451_c3_g6_i1 | 201 | 16 | 0,07960199 |
| TRINITY_DN15975_c0_g1_i2 | 614 | 49 | 0,07980456 |
| TRINITY_DN14445_c1_g16_i1 | 288 | 23 | 0,07986111 |
| TRINITY_DN3441_c0_g1_i1 | 150 | 12 | 0,08 |
| TRINITY_DN17619_c0_g3_i3 | 298 | 24 | 0,08053691 |
| TRINITY_DN15145_c0_g1_i4 | 434 | 35 | 0,08064516 |
| TRINITY_DN14389_c0_g1_i1 | 186 | 15 | 0,08064516 |
| TRINITY_DN9488_c0_g1_i1 | 186 | 15 | 0,08064516 |
| TRINITY_DN17740_c0_g1_i1 | 1099 | 89 | 0,08098271 |
| TRINITY_DN17371_c0_g1_i3 | 234 | 19 | 0,08119658 |
| TRINITY_DN7586_c0_g1_i1 | 28172 | 2292 | 0,08135738 |
| TRINITY_DN15132_c0_g1_i4 | 553 | 45 | 0,08137432 |
| TRINITY_DN17254_c0_g6_i3 | 172 | 14 | 0,08139535 |
| TRINITY_DN34658_c0_g1_i1 | 147 | 12 | 0,08163265 |
| TRINITY_DN11432_c1_g6_i2 | 2072 | 170 | 0,08204633 |
| TRINITY_DN5281_c0_g1_i3 | 195 | 16 | 0,08205128 |
| TRINITY_DN13245_c0_g1_i2 | 581 | 48 | 0,08261618 |
| TRINITY_DN17318_c0_g3_i3 | 121 | 10 | 0,08264463 |
| TRINITY_DN17318_c0_g2_i1 | 399 | 33 | 0,08270677 |
| TRINITY_DN11307_c0_g1_i1 | 374 | 31 | 0,0828877 |
| TRINITY_DN15935_c0_g1_i2 | 361 | 30 | 0,08310249 |
| TRINITY_DN17597_c2_g2_i1 | 288 | 24 | 0,08333333 |
| TRINITY_DN17318_c0_g2_i3 | 227 | 19 | 0,08370044 |
| TRINITY_DN7068_c0_g1_i1 | 143 | 12 | 0,08391608 |
| TRINITY_DN14498_c0_g2_i4 | 1830 | 155 | 0,08469945 |
| TRINITY_DN12971_c0_g1_i1 | 1414 | 120 | 0,08486563 |
| TRINITY_DN12704_c0_g1_i2 | 271 | 23 | 0,08487085 |
| TRINITY_DN12007_c0_g6_i1 | 635 | 54 | 0,08503937 |
| TRINITY_DN3108_c0_g1_i2 | 117 | 10 | 0,08547009 |
| TRINITY_DN9888_c0_g2_i1 | 152 | 13 | 0,08552632 |
| TRINITY_DN17394_c0_g9_i1 | 210 | 18 | 0,08571429 |
| TRINITY_DN16850_c0_g1_i7 | 244 | 21 | 0,08606557 |
| TRINITY_DN10858_c0_g2_i1 | 116 | 10 | 0,0862069 |
| TRINITY_DN14062_c0_g1_i1 | 695 | 60 | 0,08633094 |
| TRINITY_DN15168_c0_g3_i5 | 219 | 19 | 0,08675799 |
| TRINITY_DN17414_c0_g11_i5 | 391 | 34 | 0,08695652 |
| TRINITY_DN32004_c0_g1_i1 | 161 | 14 | 0,08695652 |
| TRINITY_DN11532_c0_g1_i5 | 160 | 14 | 0,0875 |
| TRINITY_DN14735_c0_g1_i4 | 914 | 80 | 0,08752735 |
| TRINITY_DN15059_c0_g1_i4 | 331 | 29 | 0,08761329 |
| TRINITY_DN15112_c0_g1_i3 | 114 | 10 | 0,0877193 |
| TRINITY_DN18109_c0_g1_i1 | 114 | 10 | 0,0877193 |
| TRINITY_DN603_c0_g1_i1 | 136 | 12 | 0,08823529 |
| TRINITY_DN17728_c0_g1_i3 | 113 | 10 | 0,08849558 |
| TRINITY_DN7976_c0_g1_i1 | 113 | 10 | 0,08849558 |
| TRINITY_DN16273_c0_g1_i2 | 327 | 29 | 0,08868502 |
| TRINITY_DN15864_c0_g2_i1 | 112 | 10 | 0,08928571 |
| TRINITY_DN10336_c0_g1_i2 | 156 | 14 | 0,08974359 |
| TRINITY_DN14622_c0_g1_i2 | 111 | 10 | 0,09009009 |
| TRINITY_DN13268_c0_g2_i1 | 122 | 11 | 0,09016393 |
| TRINITY_DN14174_c0_g1_i2 | 133 | 12 | 0,09022556 |
| TRINITY_DN14548_c0_g2_i1 | 764 | 69 | 0,09031414 |
| TRINITY_DN17727_c0_g1_i2 | 321 | 29 | 0,09034268 |
| TRINITY_DN10350_c0_g1_i3 | 332 | 30 | 0,09036145 |
| TRINITY_DN17511_c0_g4_i2 | 177 | 16 | 0,09039548 |
| TRINITY_DN15213_c0_g1_i4 | 121 | 11 | 0,09090909 |
| TRINITY_DN17037_c0_g7_i2 | 889 | 81 | 0,09111361 |
| TRINITY_DN17394_c0_g10_i1 | 372 | 34 | 0,09139785 |
| TRINITY_DN13384_c0_g2_i1 | 120 | 11 | 0,09166667 |
| TRINITY_DN33392_c0_g1_i1 | 120 | 11 | 0,09166667 |
| TRINITY_DN16325_c0_g7_i2 | 141 | 13 | 0,09219858 |
| TRINITY_DN15006_c0_g1_i3 | 401 | 37 | 0,09226933 |
| TRINITY_DN17078_c1_g2_i1 | 292 | 27 | 0,09246575 |
| TRINITY_DN16292_c0_g13_i4 | 324 | 30 | 0,09259259 |
| TRINITY_DN12985_c0_g1_i2 | 205 | 19 | 0,09268293 |
| TRINITY_DN15714_c0_g1_i1 | 861 | 80 | 0,09291521 |
| TRINITY_DN12130_c0_g2_i1 | 172 | 16 | 0,09302326 |
| TRINITY_DN16325_c0_g7_i3 | 129 | 12 | 0,09302326 |
| TRINITY_DN30172_c0_g1_i1 | 161 | 15 | 0,0931677 |
| TRINITY_DN17539_c0_g2_i3 | 118 | 11 | 0,09322034 |
| TRINITY_DN15552_c0_g2_i1 | 118 | 11 | 0,09322034 |
| TRINITY_DN15737_c0_g2_i3 | 193 | 18 | 0,09326425 |
| TRINITY_DN10422_c0_g4_i1 | 99781 | 9308 | 0,09328429 |
| TRINITY_DN16036_c0_g2_i1 | 139 | 13 | 0,09352518 |
| TRINITY_DN17141_c1_g3_i3 | 245 | 23 | 0,09387755 |
| TRINITY_DN17544_c0_g1_i3 | 339 | 32 | 0,09439528 |
| TRINITY_DN7824_c0_g1_i1 | 127 | 12 | 0,09448819 |
| TRINITY_DN9327_c1_g1_i3 | 169 | 16 | 0,09467456 |
| TRINITY_DN17727_c0_g2_i6 | 422 | 40 | 0,09478673 |
| TRINITY_DN14062_c0_g1_i2 | 327 | 31 | 0,09480122 |
| TRINITY_DN9305_c0_g1_i5 | 252 | 24 | 0,0952381 |
| TRINITY_DN16985_c0_g1_i2 | 210 | 20 | 0,0952381 |
| TRINITY_DN17577_c0_g4_i1 | 115 | 11 | 0,09565217 |
| TRINITY_DN2552_c0_g2_i1 | 146 | 14 | 0,09589041 |
| TRINITY_DN17404_c0_g4_i5 | 218 | 21 | 0,09633028 |
| TRINITY_DN15916_c0_g4_i5 | 114 | 11 | 0,09649123 |
| TRINITY_DN17372_c0_g2_i4 | 124 | 12 | 0,09677419 |
| TRINITY_DN16680_c0_g1_i2 | 568 | 55 | 0,09683099 |
| TRINITY_DN10079_c0_g1_i1 | 165 | 16 | 0,0969697 |
| TRINITY_DN13362_c0_g2_i1 | 175 | 17 | 0,09714286 |
| TRINITY_DN15868_c0_g3_i1 | 288 | 28 | 0,09722222 |
| TRINITY_DN12737_c0_g1_i2 | 113 | 11 | 0,09734513 |
| TRINITY_DN17394_c0_g12_i3 | 277 | 27 | 0,09747292 |
| TRINITY_DN14697_c0_g1_i4 | 123 | 12 | 0,09756098 |
| TRINITY_DN14340_c2_g11_i1 | 1531 | 150 | 0,09797518 |
| TRINITY_DN11640_c0_g1_i1 | 2905 | 285 | 0,09810671 |
| TRINITY_DN1397_c0_g1_i1 | 173 | 17 | 0,0982659 |
| TRINITY_DN10060_c0_g1_i2 | 122 | 12 | 0,09836066 |
| TRINITY_DN17136_c0_g4_i9 | 101 | 10 | 0,0990099 |
| TRINITY_DN10277_c0_g2_i1 | 242 | 24 | 0,09917355 |
| TRINITY_DN15796_c1_g1_i2 | 615 | 61 | 0,09918699 |
| TRINITY_DN14812_c0_g1_i4 | 2111 | 211 | 0,09995263 |
| TRINITY_DN12818_c0_g4_i1 | 600 | 60 | 0,1 |
| TRINITY_DN11170_c0_g3_i3 | 310 | 31 | 0,1 |
| TRINITY_DN10139_c0_g1_i1 | 310 | 31 | 0,1 |
| TRINITY_DN6275_c0_g1_i1 | 120 | 12 | 0,1 |

**A.3. Comparison: female *vs* male**

**A.3.1. Transcripts over represented in females**

| **Transcript ID** | **Female** | **Male** | **FC (Fold change)** |
| --- | --- | --- | --- |
| TRINITY_DN12575_c0_g1_i1 | 25035 | 10 | 2503,5 |
| TRINITY_DN14948_c0_g8_i1 | 31892 | 20 | 1594,6 |
| TRINITY_DN8975_c244_g1_i4 | 10647 | 13 | 819 |
| TRINITY_DN14948_c0_g16_i1 | 20264 | 25 | 810,56 |
| TRINITY_DN12527_c0_g1_i1 | 10832 | 14 | 773,714286 |
| TRINITY_DN14948_c0_g10_i2 | 11825 | 21 | 563,095238 |
| TRINITY_DN12527_c0_g1_i2 | 7149 | 15 | 476,6 |
| TRINITY_DN16897_c1_g8_i1 | 1168 | 10 | 116,8 |
| TRINITY_DN15237_c0_g4_i2 | 1378 | 12 | 114,833333 |
| TRINITY_DN12288_c0_g1_i2 | 880 | 10 | 88 |
| TRINITY_DN15712_c0_g1_i1 | 3929 | 63 | 62,3650794 |
| TRINITY_DN16566_c0_g2_i1 | 1057 | 18 | 58,7222222 |
| TRINITY_DN17105_c0_g7_i2 | 1144 | 20 | 57,2 |
| TRINITY_DN15847_c0_g1_i2 | 891 | 17 | 52,4117647 |
| TRINITY_DN12954_c0_g1_i1 | 2835 | 57 | 49,7368421 |
| TRINITY_DN15733_c4_g32_i1 | 2287 | 47 | 48,6595745 |
| TRINITY_DN15606_c0_g1_i1 | 768 | 16 | 48 |
| TRINITY_DN12882_c0_g6_i6 | 1237 | 30 | 41,2333333 |
| TRINITY_DN16390_c0_g1_i1 | 519 | 13 | 39,9230769 |
| TRINITY_DN10659_c0_g1_i2 | 1576 | 40 | 39,4 |
| TRINITY_DN12504_c0_g1_i1 | 766 | 20 | 38,3 |
| TRINITY_DN10186_c0_g1_i4 | 515 | 14 | 36,7857143 |
| TRINITY_DN17291_c0_g3_i3 | 541 | 15 | 36,0666667 |
| TRINITY_DN12006_c0_g1_i4 | 422 | 12 | 35,1666667 |
| TRINITY_DN13633_c0_g6_i1 | 475 | 14 | 33,9285714 |
| TRINITY_DN16374_c0_g16_i1 | 709 | 21 | 33,7619048 |
| TRINITY_DN17304_c0_g1_i1 | 1665 | 57 | 29,2105263 |
| TRINITY_DN16626_c0_g2_i7 | 542 | 19 | 28,5263158 |
| TRINITY_DN14538_c0_g2_i6 | 284 | 10 | 28,4 |
| TRINITY_DN15306_c0_g3_i1 | 864 | 31 | 27,8709677 |
| TRINITY_DN16314_c0_g1_i1 | 360 | 13 | 27,6923077 |
| TRINITY_DN17694_c1_g3_i1 | 264 | 10 | 26,4 |
| TRINITY_DN58_c0_g1_i1 | 290 | 11 | 26,3636364 |
| TRINITY_DN13225_c0_g1_i2 | 593 | 23 | 25,7826087 |
| TRINITY_DN16583_c0_g1_i1 | 279 | 11 | 25,3636364 |
| TRINITY_DN17744_c1_g1_i1 | 325 | 13 | 25 |
| TRINITY_DN8797_c0_g2_i1 | 3335 | 136 | 24,5220588 |
| TRINITY_DN16427_c1_g3_i4 | 292 | 12 | 24,3333333 |
| TRINITY_DN12780_c0_g2_i2 | 335 | 14 | 23,9285714 |
| TRINITY_DN17348_c0_g4_i3 | 534 | 23 | 23,2173913 |
| TRINITY_DN13940_c0_g1_i3 | 810 | 35 | 23,1428571 |
| TRINITY_DN13637_c0_g1_i2 | 693 | 30 | 23,1 |
| TRINITY_DN17606_c0_g5_i1 | 265 | 12 | 22,0833333 |
| TRINITY_DN17299_c0_g3_i1 | 440 | 20 | 22 |
| TRINITY_DN12047_c0_g1_i3 | 352 | 16 | 22 |
| TRINITY_DN13268_c0_g2_i1 | 2670 | 122 | 21,8852459 |
| TRINITY_DN13129_c0_g1_i1 | 493 | 23 | 21,4347826 |
| TRINITY_DN16616_c0_g20_i3 | 340 | 16 | 21,25 |
| TRINITY_DN15712_c0_g1_i2 | 297 | 14 | 21,2142857 |
| TRINITY_DN14392_c0_g2_i7 | 233 | 11 | 21,1818182 |
| TRINITY_DN13149_c0_g4_i5 | 360 | 17 | 21,1764706 |
| TRINITY_DN16639_c0_g5_i1 | 269 | 13 | 20,6923077 |
| TRINITY_DN17593_c0_g5_i1 | 434 | 21 | 20,6666667 |
| TRINITY_DN3260_c0_g11_i1 | 845 | 41 | 20,6097561 |
| TRINITY_DN17141_c1_g2_i1 | 1171 | 57 | 20,5438596 |
| TRINITY_DN13926_c1_g1_i5 | 546 | 27 | 20,2222222 |
| TRINITY_DN29004_c0_g1_i1 | 343 | 17 | 20,1764706 |
| TRINITY_DN14105_c0_g2_i3 | 328 | 17 | 19,2941176 |
| TRINITY_DN7905_c0_g1_i2 | 212 | 11 | 19,2727273 |
| TRINITY_DN12588_c0_g2_i3 | 308 | 16 | 19,25 |
| TRINITY_DN13566_c0_g1_i2 | 342 | 18 | 19 |
| TRINITY_DN10167_c1_g2_i1 | 190 | 10 | 19 |
| TRINITY_DN17369_c0_g14_i4 | 433 | 23 | 18,826087 |
| TRINITY_DN15599_c0_g3_i2 | 223 | 12 | 18,5833333 |
| TRINITY_DN16626_c0_g2_i3 | 221 | 12 | 18,4166667 |
| TRINITY_DN15178_c1_g7_i1 | 293 | 16 | 18,3125 |
| TRINITY_DN17175_c0_g1_i1 | 219 | 12 | 18,25 |
| TRINITY_DN6607_c0_g1_i1 | 182 | 10 | 18,2 |
| TRINITY_DN13508_c0_g5_i2 | 200 | 11 | 18,1818182 |
| TRINITY_DN17063_c0_g2_i1 | 363 | 20 | 18,15 |
| TRINITY_DN12957_c0_g2_i1 | 235 | 13 | 18,0769231 |
| TRINITY_DN16948_c0_g1_i1 | 233 | 13 | 17,9230769 |
| TRINITY_DN8717_c0_g3_i1 | 214 | 12 | 17,8333333 |
| TRINITY_DN14784_c0_g3_i1 | 406 | 23 | 17,6521739 |
| TRINITY_DN17640_c0_g1_i1 | 229 | 13 | 17,6153846 |
| TRINITY_DN17587_c1_g2_i1 | 474 | 27 | 17,5555556 |
| TRINITY_DN17085_c0_g1_i1 | 191 | 11 | 17,3636364 |
| TRINITY_DN15606_c0_g1_i2 | 445 | 26 | 17,1153846 |
| TRINITY_DN15493_c0_g4_i1 | 272 | 16 | 17 |
| TRINITY_DN17733_c1_g1_i2 | 1222 | 72 | 16,9722222 |
| TRINITY_DN16802_c0_g2_i2 | 203 | 12 | 16,9166667 |
| TRINITY_DN17566_c0_g3_i6 | 236 | 14 | 16,8571429 |
| TRINITY_DN14466_c0_g1_i1 | 318 | 19 | 16,7368421 |
| TRINITY_DN14198_c3_g11_i1 | 351 | 21 | 16,7142857 |
| TRINITY_DN15298_c0_g1_i1 | 212 | 13 | 16,3076923 |
| TRINITY_DN16374_c0_g14_i1 | 337 | 21 | 16,047619 |
| TRINITY_DN9211_c0_g3_i1 | 159 | 10 | 15,9 |
| TRINITY_DN11563_c0_g2_i2 | 1184 | 76 | 15,5789474 |
| TRINITY_DN6532_c0_g3_i3 | 1057 | 68 | 15,5441176 |
| TRINITY_DN17671_c4_g5_i1 | 2625 | 170 | 15,4411765 |
| TRINITY_DN15818_c0_g4_i1 | 154 | 10 | 15,4 |
| TRINITY_DN16810_c0_g1_i7 | 151 | 10 | 15,1 |
| TRINITY_DN15700_c0_g3_i4 | 286 | 19 | 15,0526316 |
| TRINITY_DN17206_c0_g2_i2 | 225 | 15 | 15 |
| TRINITY_DN17566_c0_g3_i1 | 254 | 17 | 14,9411765 |
| TRINITY_DN7596_c0_g1_i2 | 298 | 20 | 14,9 |
| TRINITY_DN15109_c0_g3_i5 | 208 | 14 | 14,8571429 |
| TRINITY_DN13086_c0_g2_i6 | 279 | 19 | 14,6842105 |
| TRINITY_DN12034_c0_g1_i2 | 205 | 14 | 14,6428571 |
| TRINITY_DN13273_c0_g1_i1 | 146 | 10 | 14,6 |
| TRINITY_DN16255_c0_g2_i2 | 408 | 28 | 14,5714286 |
| TRINITY_DN15644_c0_g2_i1 | 3244 | 224 | 14,4821429 |
| TRINITY_DN17614_c0_g11_i1 | 158 | 11 | 14,3636364 |
| TRINITY_DN16867_c0_g1_i2 | 2458 | 172 | 14,2906977 |
| TRINITY_DN15525_c0_g6_i1 | 1645 | 116 | 14,1810345 |
| TRINITY_DN11750_c0_g1_i1 | 366 | 26 | 14,0769231 |
| TRINITY_DN13325_c0_g1_i1 | 197 | 14 | 14,0714286 |
| TRINITY_DN12738_c0_g2_i1 | 1120 | 80 | 14 |
| TRINITY_DN16707_c0_g2_i3 | 152 | 11 | 13,8181818 |
| TRINITY_DN13521_c0_g1_i5 | 152 | 11 | 13,8181818 |
| TRINITY_DN32408_c0_g1_i1 | 204 | 15 | 13,6 |
| TRINITY_DN17671_c5_g14_i1 | 447 | 33 | 13,5454545 |
| TRINITY_DN15777_c0_g1_i5 | 241 | 18 | 13,3888889 |
| TRINITY_DN15472_c0_g2_i1 | 334 | 25 | 13,36 |
| TRINITY_DN2750_c0_g1_i1 | 173 | 13 | 13,3076923 |
| TRINITY_DN15695_c0_g1_i3 | 172 | 13 | 13,2307692 |
| TRINITY_DN4968_c0_g1_i1 | 264 | 20 | 13,2 |
| TRINITY_DN17299_c0_g3_i3 | 303 | 23 | 13,173913 |
| TRINITY_DN17263_c0_g1_i2 | 244 | 19 | 12,8421053 |
| TRINITY_DN11053_c0_g1_i6 | 141 | 11 | 12,8181818 |
| TRINITY_DN17175_c0_g1_i6 | 128 | 10 | 12,8 |
| TRINITY_DN17481_c0_g2_i1 | 766 | 60 | 12,7666667 |
| TRINITY_DN16123_c0_g5_i6 | 140 | 11 | 12,7272727 |
| TRINITY_DN17683_c0_g1_i3 | 164 | 13 | 12,6153846 |
| TRINITY_DN14581_c0_g2_i1 | 226 | 18 | 12,5555556 |
| TRINITY_DN16239_c0_g3_i2 | 125 | 10 | 12,5 |
| TRINITY_DN14642_c0_g1_i2 | 137 | 11 | 12,4545455 |
| TRINITY_DN15859_c0_g2_i4 | 124 | 10 | 12,4 |
| TRINITY_DN17367_c0_g4_i1 | 518 | 42 | 12,3333333 |
| TRINITY_DN6040_c0_g1_i1 | 123 | 10 | 12,3 |
| TRINITY_DN17401_c0_g7_i1 | 171 | 14 | 12,2142857 |
| TRINITY_DN14658_c0_g1_i2 | 122 | 10 | 12,2 |
| TRINITY_DN9789_c0_g1_i1 | 195 | 16 | 12,1875 |
| TRINITY_DN14537_c0_g1_i1 | 207 | 17 | 12,1764706 |
| TRINITY_DN14253_c0_g1_i2 | 157 | 13 | 12,0769231 |
| TRINITY_DN10558_c0_g1_i3 | 1123 | 93 | 12,0752688 |
| TRINITY_DN17608_c0_g1_i8 | 217 | 18 | 12,0555556 |
| TRINITY_DN16112_c0_g2_i2 | 289 | 24 | 12,0416667 |
| TRINITY_DN11899_c0_g1_i2 | 385 | 32 | 12,03125 |
| TRINITY_DN15912_c0_g1_i8 | 143 | 12 | 11,9166667 |
| TRINITY_DN16104_c0_g1_i2 | 131 | 11 | 11,9090909 |
| TRINITY_DN16398_c0_g3_i1 | 119 | 10 | 11,9 |
| TRINITY_DN11563_c0_g2_i1 | 2018 | 170 | 11,8705882 |
| TRINITY_DN17664_c4_g2_i5 | 261 | 22 | 11,8636364 |
| TRINITY_DN14539_c0_g2_i2 | 154 | 13 | 11,8461538 |
| TRINITY_DN13374_c0_g2_i1 | 142 | 12 | 11,8333333 |
| TRINITY_DN15264_c0_g2_i1 | 259 | 22 | 11,7727273 |
| TRINITY_DN12987_c0_g1_i2 | 1620 | 138 | 11,7391304 |
| TRINITY_DN16846_c0_g2_i2 | 164 | 14 | 11,7142857 |
| TRINITY_DN17730_c0_g1_i8 | 199 | 17 | 11,7058824 |
| TRINITY_DN8060_c0_g1_i1 | 187 | 16 | 11,6875 |
| TRINITY_DN17293_c0_g1_i7 | 163 | 14 | 11,6428571 |
| TRINITY_DN17386_c0_g3_i4 | 128 | 11 | 11,6363636 |
| TRINITY_DN10167_c1_g1_i1 | 300 | 26 | 11,5384615 |
| TRINITY_DN15632_c0_g1_i4 | 205 | 18 | 11,3888889 |
| TRINITY_DN17691_c2_g2_i3 | 431 | 38 | 11,3421053 |
| TRINITY_DN10952_c0_g1_i1 | 306 | 27 | 11,3333333 |
| TRINITY_DN15743_c0_g1_i3 | 136 | 12 | 11,3333333 |
| TRINITY_DN9763_c0_g1_i1 | 136 | 12 | 11,3333333 |
| TRINITY_DN13286_c0_g1_i3 | 147 | 13 | 11,3076923 |
| TRINITY_DN12607_c0_g4_i3 | 248 | 22 | 11,2727273 |
| TRINITY_DN17372_c0_g2_i9 | 124 | 11 | 11,2727273 |
| TRINITY_DN16511_c0_g1_i2 | 135 | 12 | 11,25 |
| TRINITY_DN8975_c220_g1_i3 | 5807 | 518 | 11,2104247 |
| TRINITY_DN16225_c0_g3_i3 | 112 | 10 | 11,2 |
| TRINITY_DN17446_c0_g1_i1 | 123 | 11 | 11,1818182 |
| TRINITY_DN12212_c0_g3_i1 | 267 | 24 | 11,125 |
| TRINITY_DN17670_c1_g1_i2 | 244 | 22 | 11,0909091 |
| TRINITY_DN16819_c0_g3_i1 | 133 | 12 | 11,0833333 |
| TRINITY_DN16048_c0_g3_i1 | 155 | 14 | 11,0714286 |
| TRINITY_DN15472_c0_g6_i1 | 541 | 49 | 11,0408163 |
| TRINITY_DN13809_c0_g3_i1 | 251 | 23 | 10,9130435 |
| TRINITY_DN15525_c0_g6_i3 | 716 | 66 | 10,8484848 |
| TRINITY_DN17655_c1_g1_i1 | 711 | 66 | 10,7727273 |
| TRINITY_DN17691_c1_g1_i1 | 204 | 19 | 10,7368421 |
| TRINITY_DN16508_c1_g1_i1 | 118 | 11 | 10,7272727 |
| TRINITY_DN15178_c1_g7_i6 | 107 | 10 | 10,7 |
| TRINITY_DN13963_c0_g1_i6 | 192 | 18 | 10,6666667 |
| TRINITY_DN14957_c0_g4_i1 | 106 | 10 | 10,6 |
| TRINITY_DN17054_c0_g5_i1 | 286 | 27 | 10,5925926 |
| TRINITY_DN17040_c0_g1_i1 | 349 | 33 | 10,5757576 |
| TRINITY_DN17618_c0_g4_i2 | 158 | 15 | 10,5333333 |
| TRINITY_DN15128_c0_g1_i6 | 126 | 12 | 10,5 |
| TRINITY_DN14394_c0_g5_i2 | 157 | 15 | 10,4666667 |
| TRINITY_DN17449_c0_g2_i1 | 292 | 28 | 10,4285714 |
| TRINITY_DN16897_c0_g3_i2 | 177 | 17 | 10,4117647 |
| TRINITY_DN17739_c1_g2_i1 | 363 | 35 | 10,3714286 |
| TRINITY_DN17054_c0_g1_i1 | 114 | 11 | 10,3636364 |
| TRINITY_DN10121_c2_g1_i3 | 949 | 92 | 10,3152174 |
| TRINITY_DN17204_c0_g3_i3 | 216 | 21 | 10,2857143 |
| TRINITY_DN13647_c0_g1_i5 | 665 | 65 | 10,2307692 |
| TRINITY_DN17329_c2_g1_i4 | 143 | 14 | 10,2142857 |
| TRINITY_DN13955_c0_g1_i1 | 132 | 13 | 10,1538462 |
| TRINITY_DN16928_c0_g1_i2 | 203 | 20 | 10,15 |
| TRINITY_DN16653_c3_g17_i1 | 859 | 85 | 10,1058824 |
| TRINITY_DN14335_c0_g1_i1 | 202 | 20 | 10,1 |
| TRINITY_DN17632_c0_g1_i6 | 101 | 10 | 10,1 |
| TRINITY_DN17653_c0_g2_i1 | 232 | 23 | 10,0869565 |
| TRINITY_DN17317_c0_g1_i3 | 221 | 22 | 10,0454545 |
| TRINITY_DN16660_c0_g2_i9 | 231 | 23 | 10,0434783 |
| TRINITY_DN15074_c0_g9_i1 | 411 | 41 | 10,0243902 |
| TRINITY_DN13055_c0_g1_i4 | 841 | 84 | 10,0119048 |
| TRINITY_DN17448_c0_g16_i2 | 190 | 19 | 10 |
| TRINITY_DN13879_c0_g1_i2 | 110 | 11 | 10 |

**A.3. Comparison: female *vs* male**

**A.3.2. Transcripts over represented in males**

| **Transcript ID** | **Female** | | **Male** | | **FC** | | |
| --- | --- | --- | --- | --- | --- | --- | --- |
| TRINITY_DN11354_c1_g4_i1 | 10 | | 7714 | | | | 0,001296 |
| TRINITY_DN15181_c0_g3_i1 | 28 | | 6242 | | | | 0,004486 |
| TRINITY_DN16930_c1_g1_i3 | 13 | | 1821 | | | | 0,007139 |
| TRINITY_DN15733_c4_g31_i1 | 10 | | 1227 | | | | 0,00815 |
| TRINITY_DN15050_c0_g1_i4 | 34 | | 3845 | | | | 0,008843 |
| TRINITY_DN10750_c0_g1_i2 | 25 | | 2297 | | | | 0,010884 |
| TRINITY_DN10546_c0_g1_i3 | 11 | | 992 | | | | 0,011089 |
| TRINITY_DN15050_c0_g1_i1 | 43 | | 3766 | | | | 0,011418 |
| TRINITY_DN17514_c0_g6_i2 | 12 | | 895 | | | | 0,013408 |
| TRINITY_DN6541_c0_g1_i1 | 20 | | 1333 | | | | 0,015004 |
| TRINITY_DN11354_c2_g5_i1 | 129 | | 8098 | | | | 0,01593 |
| TRINITY_DN7179_c0_g3_i1 | 26 | | 1587 | | | | 0,016383 |
| TRINITY_DN6541_c0_g2_i1 | 23 | | 1353 | | | | 0,016999 |
| TRINITY_DN16444_c0_g2_i1 | 22 | | 1267 | | | | 0,017364 |
| TRINITY_DN16168_c0_g7_i2 | 10 | | 535 | | | | 0,018692 |
| TRINITY_DN15425_c0_g1_i3 | 15 | | 790 | | | | 0,018987 |
| TRINITY_DN17361_c0_g6_i4 | 10 | | 518 | | | | 0,019305 |
| TRINITY_DN9842_c0_g1_i2 | 17 | | 826 | | | | 0,020581 |
| TRINITY_DN16361_c0_g6_i1 | 37 | | 1743 | | | | 0,021228 |
| TRINITY_DN13633_c0_g11_i1 | 14 | | 638 | | | | 0,021944 |
| TRINITY_DN17514_c0_g10_i2 | 12 | | 516 | | | | 0,023256 |
| TRINITY_DN17060_c1_g8_i1 | 17 | | 729 | | | | 0,02332 |
| TRINITY_DN12024_c0_g1_i3 | 74 | | 3079 | | | | 0,024034 |
| TRINITY_DN13532_c0_g1_i6 | 12 | | 493 | | | | 0,024341 |
| TRINITY_DN12145_c0_g1_i3 | 16 | | 619 | | | | 0,025848 |
| TRINITY_DN10456_c0_g2_i2 | 13 | | 494 | | | | 0,026316 |
| TRINITY_DN15490_c0_g2_i1 | 23 | | 869 | | | | 0,026467 |
| TRINITY_DN16999_c0_g2_i1 | 17 | | 617 | | | | 0,027553 |
| TRINITY_DN16489_c0_g3_i1 | 23 | | 805 | | | | 0,028571 |
| TRINITY_DN15093_c0_g2_i2 | 38 | | 1324 | | | | 0,028701 |
| TRINITY_DN17452_c0_g3_i1 | 22 | | 763 | | | | 0,028834 |
| TRINITY_DN12582_c0_g2_i1 | 12 | | 415 | | | | 0,028916 |
| TRINITY_DN13581_c0_g1_i2 | 29 | | 997 | | | | 0,029087 |
| TRINITY_DN15050_c0_g1_i2 | 58 | | 1920 | | | | 0,030208 |
| TRINITY_DN13095_c0_g5_i1 | 14 | | 457 | | | | 0,030635 |
| TRINITY_DN17727_c0_g2_i6 | 13 | | 422 | | | | 0,030806 |
| TRINITY_DN16679_c0_g3_i5 | 26 | | 836 | | | | 0,0311 |
| TRINITY_DN13532_c0_g1_i7 | 12 | | 381 | | | | 0,031496 |
| TRINITY_DN13360_c0_g1_i2 | 10 | | 317 | | | | 0,031546 |
| TRINITY_DN3670_c0_g1_i1 | 15 | | 472 | | | | 0,03178 |
| TRINITY_DN15085_c0_g3_i2 | 30 | | 898 | | | | 0,033408 |
| TRINITY_DN17727_c0_g2_i5 | 13 | | 378 | | | | 0,034392 |
| TRINITY_DN599_c0_g1_i1 | 14 | | 404 | | | | 0,034653 |
| TRINITY_DN17410_c0_g5_i2 | 14 | | 402 | | | | 0,034826 |
| TRINITY_DN17514_c0_g1_i4 | 13 | | 373 | | | | 0,034853 |
| TRINITY_DN16502_c0_g1_i4 | 40 | | 1120 | | | | 0,035714 |
| TRINITY_DN17307_c1_g1_i1 | | 16 | | 442 | |  |  |
| TRINITY_DN10842_c0_g1_i1 | 67 | | 1838 | | | | 0,036453 |
| TRINITY_DN17727_c0_g1_i2 | 12 | | 321 | | | | 0,037383 |
| TRINITY_DN14438_c0_g1_i1 | 22 | | 575 | | | | 0,038261 |
| TRINITY_DN16489_c0_g4_i3 | 11 | | 286 | | | | 0,038462 |
| TRINITY_DN11120_c0_g4_i1 | 31 | | 777 | | | | 0,039897 |
| TRINITY_DN14767_c0_g1_i2 | 37 | | 907 | | | | 0,040794 |
| TRINITY_DN14174_c0_g1_i4 | 11 | | 264 | | | | 0,041667 |
| TRINITY_DN15006_c0_g1_i1 | 14 | | 333 | | | | 0,042042 |
| TRINITY_DN11845_c0_g1_i1 | 11 | | 258 | | | | 0,042636 |
| TRINITY_DN12818_c0_g3_i1 | 22 | | 503 | | | | 0,043738 |
| TRINITY_DN17452_c0_g3_i3 | 30 | | 685 | | | | 0,043796 |
| TRINITY_DN17410_c0_g4_i4 | 39 | | 878 | | | | 0,044419 |
| TRINITY_DN14294_c0_g1_i1 | 25 | | 562 | | | | 0,044484 |
| TRINITY_DN17617_c0_g6_i2 | 13 | | 292 | | | | 0,044521 |
| TRINITY_DN13525_c0_g1_i1 | 17 | | 374 | | | | 0,045455 |
| TRINITY_DN15969_c0_g1_i4 | 11 | | 240 | | | | 0,045833 |
| TRINITY_DN17140_c1_g8_i3 | 34 | | 740 | | | | 0,045946 |
| TRINITY_DN14777_c0_g2_i1 | 22 | | 472 | | | | 0,04661 |
| TRINITY_DN15413_c0_g1_i1 | 10 | | 208 | | | | 0,048077 |
| TRINITY_DN17410_c0_g4_i8 | 15 | | 311 | | | | 0,048232 |
| TRINITY_DN17114_c0_g2_i2 | 25 | | 515 | | | | 0,048544 |
| TRINITY_DN8975_c105_g1_i1 | 32 | | 657 | | | | 0,048706 |
| TRINITY_DN15714_c0_g1_i1 | 42 | | 861 | | | | 0,04878 |
| TRINITY_DN10842_c0_g3_i4 | 31 | | 635 | | | | 0,048819 |
| TRINITY_DN15179_c2_g4_i3 | 280 | | 5626 | | | | 0,049769 |
| TRINITY_DN14624_c0_g2_i1 | 25 | | 502 | | | | 0,049801 |
| TRINITY_DN10750_c0_g1_i3 | 33 | | 656 | | | | 0,050305 |
| TRINITY_DN17665_c0_g1_i2 | 18 | | 357 | | | | 0,05042 |
| TRINITY_DN16523_c0_g3_i1 | 38 | | 753 | | | | 0,050465 |
| TRINITY_DN15928_c1_g1_i1 | 241 | | 4726 | | | | 0,050994 |
| TRINITY_DN11817_c0_g1_i4 | 31 | | 607 | | | | 0,051071 |
| TRINITY_DN15179_c2_g3_i1 | 978 | | 19106 | | | | 0,051188 |
| TRINITY_DN11627_c0_g2_i1 | 13 | | 252 | | | | 0,051587 |
| TRINITY_DN15179_c2_g8_i1 | 364 | | 6983 | | | | 0,052127 |
| TRINITY_DN11233_c0_g1_i3 | 19 | | 364 | | | | 0,052198 |
| TRINITY_DN12943_c0_g2_i1 | 18 | | 344 | | | | 0,052326 |
| TRINITY_DN15499_c0_g5_i1 | 21 | | 401 | | | | 0,052369 |
| TRINITY_DN15181_c2_g4_i1 | 165 | | 3125 | | | | 0,0528 |
| TRINITY_DN14293_c0_g7_i1 | 10 | | 189 | | | | 0,05291 |
| TRINITY_DN13136_c0_g1_i5 | 76 | | 1435 | | | | 0,052962 |
| TRINITY_DN13991_c0_g4_i3 | 15 | | 279 | | | | 0,053763 |
| TRINITY_DN17637_c0_g12_i2 | 17 | | 312 | | | | 0,054487 |
| TRINITY_DN16050_c0_g3_i1 | 19 | | 348 | | | | 0,054598 |
| TRINITY_DN3274_c0_g1_i1 | 23 | | 419 | | | | 0,054893 |
| TRINITY_DN16992_c0_g3_i3 | 31 | | 555 | | | | 0,055856 |
| TRINITY_DN8975_c211_g6_i1 | 66 | | 1177 | | | | 0,056075 |
| TRINITY_DN14365_c0_g5_i2 | 20 | | 354 | | | | 0,056497 |
| TRINITY_DN17727_c0_g1_i1 | 28 | | 491 | | | | 0,057026 |
| TRINITY_DN11685_c1_g3_i2 | 16 | | 280 | | | | 0,057143 |
| TRINITY_DN17665_c0_g3_i3 | 16 | | 277 | | | | 0,057762 |
| TRINITY_DN15425_c0_g1_i4 | 108 | | 1843 | | | | 0,0586 |
| TRINITY_DN10750_c0_g1_i1 | 21 | | 357 | | | | 0,058824 |
| TRINITY_DN17373_c0_g3_i4 | 10 | | 169 | | | | 0,059172 |
| TRINITY_DN9958_c0_g1_i1 | 20 | | 337 | | | | 0,059347 |
| TRINITY_DN12498_c0_g3_i1 | 2052 | | 33903 | | | | 0,060526 |
| TRINITY_DN14438_c0_g1_i2 | 74 | | 1216 | | | | 0,060855 |
| TRINITY_DN15916_c0_g4_i3 | 30 | | 490 | | | | 0,061224 |
| TRINITY_DN17361_c0_g6_i3 | 19 | | 309 | | | | 0,061489 |
| TRINITY_DN17373_c0_g6_i2 | 12 | | 195 | | | | 0,061538 |
| TRINITY_DN4842_c0_g1_i3 | 10 | | 161 | | | | 0,062112 |
| TRINITY_DN17665_c0_g9_i6 | 15 | | 241 | | | | 0,062241 |
| TRINITY_DN16767_c1_g1_i3 | 10 | | 160 | | | | 0,0625 |
| TRINITY_DN11606_c0_g1_i1 | 11 | | 175 | | | | 0,062857 |
| TRINITY_DN17017_c0_g1_i1 | 42 | | 666 | | | | 0,063063 |
| TRINITY_DN9463_c0_g3_i2 | 21 | | 333 | | | | 0,063063 |
| TRINITY_DN17440_c0_g3_i2 | 37 | | 583 | | | | 0,063465 |
| TRINITY_DN9412_c0_g1_i1 | 12 | | 188 | | | | 0,06383 |
| TRINITY_DN17647_c0_g1_i2 | 18 | | 280 | | | | 0,064286 |
| TRINITY_DN19137_c0_g1_i1 | 10 | | 155 | | | | 0,064516 |
| TRINITY_DN11817_c0_g1_i3 | 60 | | 914 | | | | 0,065646 |
| TRINITY_DN17361_c0_g6_i6 | 36 | | 543 | | | | 0,066298 |
| TRINITY_DN4909_c0_g2_i1 | 79 | | 1186 | | | | 0,06661 |
| TRINITY_DN13492_c0_g1_i2 | 16 | | 240 | | | | 0,066667 |
| TRINITY_DN23398_c0_g2_i1 | 23 | | 344 | | | | 0,06686 |
| TRINITY_DN12819_c0_g1_i1 | 77 | | 1149 | | | | 0,067015 |
| TRINITY_DN16183_c0_g2_i3 | 19 | | 283 | | | | 0,067138 |
| TRINITY_DN17331_c0_g1_i5 | 17 | | 253 | | | | 0,067194 |
| TRINITY_DN17704_c1_g1_i4 | 15 | | 218 | | | | 0,068807 |
| TRINITY_DN16168_c0_g6_i14 | 25 | | 362 | | | | 0,069061 |
| TRINITY_DN14763_c0_g2_i7 | 11 | | 159 | | | | 0,069182 |
| TRINITY_DN8944_c0_g2_i2 | 17 | | 245 | | | | 0,069388 |
| TRINITY_DN16523_c0_g1_i2 | 59 | | 847 | | | | 0,069658 |
| TRINITY_DN16166_c0_g6_i4 | 35 | | 502 | | | | 0,069721 |
| TRINITY_DN15984_c0_g9_i1 | 12 | | 171 | | | | 0,070175 |
| TRINITY_DN17586_c0_g2_i2 | 29 | | 409 | | | | 0,070905 |
| TRINITY_DN17701_c1_g2_i7 | 14 | | 197 | | | | 0,071066 |
| TRINITY_DN17232_c0_g1_i1 | 35 | | 491 | | | | 0,071283 |
| TRINITY_DN16555_c0_g2_i1 | 27 | | 377 | | | | 0,071618 |
| TRINITY_DN17479_c0_g10_i1 | 16 | | 223 | | | | 0,071749 |
| TRINITY_DN17365_c0_g2_i8 | 20 | | 278 | | | | 0,071942 |
| TRINITY_DN16911_c0_g1_i4 | 23 | | 319 | | | | 0,0721 |
| TRINITY_DN28173_c0_g1_i1 | 20 | | 277 | | | | 0,072202 |
| TRINITY_DN14497_c0_g3_i1 | 23 | | 318 | | | | 0,072327 |
| TRINITY_DN11233_c0_g1_i4 | 16 | | 220 | | | | 0,072727 |
| TRINITY_DN15152_c0_g1_i1 | 11 | | 151 | | | | 0,072848 |
| TRINITY_DN14584_c0_g1_i2 | 99 | | 1346 | | | | 0,073551 |
| TRINITY_DN16361_c0_g17_i1 | 11 | | 149 | | | | 0,073826 |
| TRINITY_DN16078_c0_g3_i3 | 17 | | 230 | | | | 0,073913 |
| TRINITY_DN8791_c0_g1_i1 | 19 | | 257 | | | | 0,07393 |
| TRINITY_DN31626_c0_g1_i1 | 12 | | 162 | | | | 0,074074 |
| TRINITY_DN17525_c0_g1_i3 | 18 | | 242 | | | | 0,07438 |
| TRINITY_DN11439_c0_g2_i1 | 16 | | 215 | | | | 0,074419 |
| TRINITY_DN17394_c0_g12_i2 | 12 | | 161 | | | | 0,074534 |
| TRINITY_DN8975_c231_g1_i1 | 269 | | 3596 | | | | 0,074805 |
| TRINITY_DN16502_c0_g1_i6 | 20 | | 267 | | | | 0,074906 |
| TRINITY_DN15452_c0_g1_i1 | 28 | | 371 | | | | 0,075472 |
| TRINITY_DN14735_c0_g1_i4 | 69 | | 914 | | | | 0,075492 |
| TRINITY_DN15616_c2_g1_i1 | 18 | | 238 | | | | 0,07563 |
| TRINITY_DN12869_c0_g3_i1 | 14 | | 184 | | | | 0,076087 |
| TRINITY_DN17719_c0_g1_i5 | 17 | | 223 | | | | 0,076233 |
| TRINITY_DN13561_c0_g1_i2 | 19 | | 249 | | | | 0,076305 |
| TRINITY_DN13584_c0_g1_i3 | 10 | | 131 | | | | 0,076336 |
| TRINITY_DN12498_c0_g2_i1 | 57 | | 746 | | | | 0,076408 |
| TRINITY_DN12517_c0_g6_i2 | 17 | | 222 | | | | 0,076577 |
| TRINITY_DN13532_c0_g1_i1 | 21 | | 271 | | | | 0,077491 |
| TRINITY_DN15579_c0_g5_i1 | 67 | | 862 | | | | 0,077726 |
| TRINITY_DN9603_c0_g4_i1 | 13 | | 166 | | | | 0,078313 |
| TRINITY_DN8759_c0_g3_i1 | 35 | | 442 | | | | 0,079186 |
| TRINITY_DN17140_c1_g5_i1 | 34 | | 425 | | | | 0,08 |
| TRINITY_DN15139_c0_g3_i1 | 14 | | 175 | | | | 0,08 |
| TRINITY_DN8975_c212_g6_i1 | 33 | | 411 | | | | 0,080292 |
| TRINITY_DN16886_c1_g5_i1 | 12 | | 149 | | | | 0,080537 |
| TRINITY_DN16744_c0_g4_i1 | 73 | | 906 | | | | 0,080574 |
| TRINITY_DN14733_c0_g2_i2 | 17 | | 210 | | | | 0,080952 |
| TRINITY_DN15181_c2_g15_i1 | 27 | | 333 | | | | 0,081081 |
| TRINITY_DN16502_c0_g1_i2 | 108 | | 1314 | | | | 0,082192 |
| TRINITY_DN15416_c0_g1_i6 | 25 | | 303 | | | | 0,082508 |
| TRINITY_DN1735_c1_g1_i1 | 10 | | 121 | | | | 0,082645 |
| TRINITY_DN16899_c0_g1_i3 | 86 | | 1034 | | | | 0,083172 |
| TRINITY_DN33392_c0_g1_i1 | 10 | | 120 | | | | 0,083333 |
| TRINITY_DN7826_c0_g1_i2 | 23 | | 275 | | | | 0,083636 |
| TRINITY_DN8504_c0_g1_i1 | 20 | | 239 | | | | 0,083682 |
| TRINITY_DN12498_c0_g4_i1 | 291 | | 3475 | | | | 0,083741 |
| TRINITY_DN10961_c0_g1_i1 | 11 | | 131 | | | | 0,083969 |
| TRINITY_DN13561_c0_g2_i4 | 18 | | 213 | | | | 0,084507 |
| TRINITY_DN17339_c0_g11_i1 | 23 | | 272 | | | | 0,084559 |
| TRINITY_DN16902_c0_g2_i8 | 11 | | 130 | | | | 0,084615 |
| TRINITY_DN17281_c0_g8_i1 | 26 | | 307 | | | | 0,084691 |
| TRINITY_DN16851_c0_g3_i2 | 82 | | 966 | | | | 0,084886 |
| TRINITY_DN16183_c0_g2_i4 | 25 | | 294 | | | | 0,085034 |
| TRINITY_DN17394_c0_g6_i5 | 16 | | 188 | | | | 0,085106 |
| TRINITY_DN16325_c0_g7_i2 | 12 | | 141 | | | | 0,085106 |
| TRINITY_DN17585_c1_g3_i4 | 32 | | 372 | | | | 0,086022 |
| TRINITY_DN17125_c1_g1_i3 | 18 | | 208 | | | | 0,086538 |
| TRINITY_DN13194_c0_g1_i7 | 29 | | 334 | | | | 0,086826 |
| TRINITY_DN12720_c0_g6_i1 | 11 | | 126 | | | | 0,087302 |
| TRINITY_DN15478_c0_g2_i1 | 29 | | 331 | | | | 0,087613 |
| TRINITY_DN16945_c0_g1_i1 | 17 | | 194 | | | | 0,087629 |
| TRINITY_DN16134_c0_g2_i15 | 14 | | 159 | | | | 0,08805 |
| TRINITY_DN5542_c1_g1_i1 | 15 | | 170 | | | | 0,088235 |
| TRINITY_DN25792_c0_g1_i1 | 13 | | 147 | | | | 0,088435 |
| TRINITY_DN15969_c0_g1_i2 | 22 | | 248 | | | | 0,08871 |
| TRINITY_DN15550_c0_g1_i4 | 97 | | 1087 | | | | 0,089236 |
| TRINITY_DN17619_c0_g3_i2 | 15 | | 168 | | | | 0,089286 |
| TRINITY_DN15397_c2_g7_i1 | 15 | | 167 | | | | 0,08982 |
| TRINITY_DN16738_c0_g1_i4 | 15 | | 167 | | | | 0,08982 |
| TRINITY_DN15733_c4_g35_i1 | 18 | | 200 | | | | 0,09 |
| TRINITY_DN16487_c0_g1_i7 | 10 | | 111 | | | | 0,09009 |
| TRINITY_DN17444_c1_g5_i3 | 122 | | 1354 | | | | 0,090103 |
| TRINITY_DN16570_c0_g2_i13 | 16 | | 177 | | | | 0,090395 |
| TRINITY_DN13085_c0_g1_i1 | 65 | | 717 | | | | 0,090656 |
| TRINITY_DN14155_c0_g3_i3 | 17 | | 187 | | | | 0,090909 |
| TRINITY_DN3953_c0_g1_i1 | 10 | | 110 | | | | 0,090909 |
| TRINITY_DN15397_c2_g3_i1 | 28 | | 306 | | | | 0,091503 |
| TRINITY_DN16117_c0_g2_i5 | 12 | | 131 | | | | 0,091603 |
| TRINITY_DN10499_c0_g1_i1 | 12 | | 131 | | | | 0,091603 |
| TRINITY_DN9888_c0_g2_i1 | 14 | | 152 | | | | 0,092105 |
| TRINITY_DN3152_c0_g2_i1 | 13 | | 141 | | | | 0,092199 |
| TRINITY_DN8650_c0_g1_i2 | 51 | | 553 | | | | 0,092224 |
| TRINITY_DN17519_c0_g1_i1 | 20 | | 214 | | | | 0,093458 |
| TRINITY_DN15913_c0_g5_i1 | 18 | | 192 | | | | 0,09375 |
| TRINITY_DN17060_c1_g2_i1 | 14 | | 149 | | | | 0,09396 |
| TRINITY_DN12206_c0_g1_i7 | 14 | | 149 | | | | 0,09396 |
| TRINITY_DN17701_c0_g3_i1 | 10 | | 106 | | | | 0,09434 |
| TRINITY_DN14486_c0_g1_i2 | 54 | | 571 | | | | 0,094571 |
| TRINITY_DN17223_c0_g10_i4 | 18 | | 190 | | | | 0,094737 |
| TRINITY_DN13095_c0_g3_i1 | 28 | | 295 | | | | 0,094915 |
| TRINITY_DN11349_c0_g1_i3 | 23 | | 242 | | | | 0,095041 |
| TRINITY_DN16497_c1_g2_i1 | 30 | | 315 | | | | 0,095238 |
| TRINITY_DN17456_c0_g1_i6 | 13 | | 136 | | | | 0,095588 |
| TRINITY_DN14158_c0_g1_i2 | 11 | | 115 | | | | 0,095652 |
| TRINITY_DN17297_c0_g5_i5 | 23 | | 240 | | | | 0,095833 |
| TRINITY_DN12928_c0_g1_i3 | 29 | | 302 | | | | 0,096026 |
| TRINITY_DN22067_c0_g1_i1 | 10 | | 104 | | | | 0,096154 |
| TRINITY_DN13555_c0_g1_i1 | 15 | | 155 | | | | 0,096774 |
| TRINITY_DN10406_c1_g1_i2 | 22 | | 227 | | | | 0,096916 |
| TRINITY_DN13548_c0_g1_i4 | 11 | | 113 | | | | 0,097345 |
| TRINITY_DN17147_c0_g1_i1 | 69 | | 708 | | | | 0,097458 |
| TRINITY_DN17091_c0_g1_i1 | 70 | | 717 | | | | 0,097629 |
| TRINITY_DN7114_c1_g1_i1 | 17 | | 174 | | | | 0,097701 |
| TRINITY_DN16902_c0_g1_i1 | 35 | | 358 | | | | 0,097765 |
| TRINITY_DN10974_c0_g1_i1 | 18 | | 184 | | | | 0,097826 |
| TRINITY_DN16232_c0_g1_i3 | 37 | | 378 | | | | 0,097884 |
| TRINITY_DN4171_c0_g1_i1 | 10 | | 102 | | | | 0,098039 |
| TRINITY_DN12005_c0_g5_i1 | 10 | | 102 | | | | 0,098039 |
| TRINITY_DN12734_c0_g2_i1 | 32 | | 326 | | | | 0,09816 |
| TRINITY_DN12323_c1_g2_i1 | 11 | | 112 | | | | 0,098214 |
| TRINITY_DN17704_c2_g1_i2 | 11 | | 112 | | | | 0,098214 |
| TRINITY_DN15737_c0_g2_i3 | 19 | | 193 | | | | 0,098446 |
| TRINITY_DN13143_c0_g2_i4 | 242 | | 2458 | | | | 0,098454 |
| TRINITY_DN16903_c0_g1_i8 | 15 | | 152 | | | | 0,098684 |
| TRINITY_DN4867_c0_g3_i1 | 62 | | 628 | | | | 0,098726 |
| TRINITY_DN16423_c0_g10_i2 | 28 | | 283 | | | | 0,09894 |
| TRINITY_DN14748_c0_g1_i3 | 10 | | 101 | | | | 0,09901 |
| TRINITY_DN14173_c0_g1_i3 | 21 | | 212 | | | | 0,099057 |
| TRINITY_DN16901_c0_g1_i3 | 14 | | 141 | | | | 0,099291 |
| TRINITY_DN17114_c0_g7_i1 | 23 | | 231 | | | | 0,099567 |
| TRINITY_DN12704_c0_g1_i3 | 49 | | 492 | | | | 0,099593 |
| TRINITY_DN17714_c0_g5_i3 | 84 | | 843 | | | | 0,099644 |
| TRINITY_DN15653_c0_g1_i1 | 162 | | 1622 | | | | 0,099877 |
| TRINITY_DN14700_c0_g1_i3 | 25 | | 250 | | | | 0,1 |
| TRINITY_DN16933_c0_g2_i1 | 12 | | 120 | | | | 0,1 |
| TRINITY_DN3839_c0_g1_i1 | 11 | | 110 | | | | 0,1 |

**B. Functional annotation of differentially represented transcripts.**

|  | **counts per million** | | |  |  |  |  |  |
| --- | --- | --- | --- | --- | --- | --- | --- | --- |
| **Sequence** | **Females** | **Males** | **Eggs** | **FC** | **GO** | **BlastHit** | **E-value** | **Annotation** |
| TRINITY_DN17587_c1_g2_i1 | 474 | 27 | - | 17,556 | - - | PREDICTED: ATP-sensitive inward rectifier potassium channel 12-like isoform X3 [Ceratitis capitata] | 0 | - |
| TRINITY_DN12588_c0_g2_i3 | 308 | 16 | - | 19,25 | GO:0005747 nadh dehydrogenase | PREDICTED: NADH dehydrogenase [ubiquinone] iron-sulfur protein 5 [Bactrocera cucurbitae] | 2E-44 | - |
| TRINITY_DN16948_c0_g1_i1 | 233 | 13 | - | 17,923 | GO:0051028 cytoplasmic dynein 1 light intermediate chain 1 | PREDICTED: cytoplasmic dynein 1 light intermediate chain 1 [Bactrocera dorsalis] | 0 | - |
| TRINITY_DN8717_c0_g3_i1 | 214 | 12 | - | 17,833 | GO:0000790 cg17950 | PREDICTED: high mobility group protein D [Bactrocera dorsalis] | 1E-23 | - |
| TRINITY_DN15306_c0_g3_i1 | 864 | 31 | - | 27,871 | GO:0005615 ecdysteroid-regulated 16 kda protein | PREDICTED: ecdysteroid-regulated 16 kDa protein [Bactrocera cucurbitae] | 3E-68 | - |
| TRINITY_DN13086_c0_g2_i6 | 279 | 19 | - | 14,684 | GO:0000398 probable small nuclear ribonucleoprotein e | PREDICTED: probable small nuclear ribonucleoprotein E-like [Ceratitis capitata]<>PREDICTED: probable small nuclear ribonucleoprotein E [Bactrocera cucurbitae]<>PREDICTED: probable small nuclear ribonucleoprotein E [Bactrocera dorsalis] | 3E-46 | - |
| TRINITY_DN17733_c1_g1_i2 | 1222 | 72 | - | 16,972 | GO:0008236 venom dipeptidyl peptidase 4 isoform x1 | PREDICTED: venom dipeptidyl peptidase 4-like isoform X2 [Ceratitis capitata] | 0 | - |
| TRINITY_DN13225_c0_g1_i2 | 593 | 23 | - | 25,783 | GO:0019013 heterogeneous nuclear ribonucleoprotein 27c | PREDICTED: heterogeneous nuclear ribonucleoprotein 27C [Bactrocera cucurbitae]<>PREDICTED: heterogeneous nuclear ribonucleoprotein 27C [Bactrocera dorsalis] | 2E-176 | - |
| TRINITY_DN15712_c0_g1_i1 | 3929 | 63 | - | 62,365 | - | PREDICTED: uncharacterized protein LOC105228225 isoform X2 [Bactrocera dorsalis] | 0.0 | - |
| TRINITY_DN10186_c0_g1_i4 | 515 | 14 | - | 36,786 | GO:000587 heat shock protein 27 | PREDICTED: heat shock protein 27 [Bactrocera cucurbitae] | 9E-84 | - |
| TRINITY_DN13129_c0_g1_i1 | 493 | 23 | - | 21,435 | - - | PREDICTED: gametocyte-specific factor 1-like [Bactrocera cucurbitae] | 1E-46 | - |
| TRINITY_DN16626_c0_g2_i7 | 542 | 19 | - | 28,526 | - | PREDICTED: inositol-trisphosphate 3-kinase A [Bactrocera dorsalis] | 0.0 | - |
| TRINITY_DN12954_c0_g1_i1 | 2835 | 57 | - | 49,737 | GO:0007049 g2 mitotic-specific cyclin-b | PREDICTED: G2/mitotic-specific cyclin-B [Bactrocera dorsalis] | 0.0 | - |
| TRINITY_DN17566_c0_g3_i6 | 236 | 14 | - | 16,857 | - - | PREDICTED: protein Smaug isoform X2 [Bactrocera cucurbitae] | 0 | - |
| TRINITY_DN7596_c0_g1_i2 | 298 | 20 | - | 14,9 | GO:0000022 60s ribosomal protein l9 | PREDICTED: 60S ribosomal protein L9 [Bactrocera dorsalis] | 7E-97 | - |
| TRINITY_DN15644_c0_g2_i1 | 3244 | 224 | - | 14,482 | GO:0003676 maternal protein exuperantia | PREDICTED: maternal protein exuperantia [Bactrocera cucurbitae] | 0 | - |
| TRINITY_DN13149_c0_g4_i5 | 360 | 17 | - | 21,176 | GO:0003333 amino acid transmembrane transporter | PREDICTED: proton-coupled amino acid transporter 4 [Bactrocera dorsalis] | 0.0 | - |
| TRINITY_DN12034_c0_g1_i2 | 205 | 14 | - | 14,643 | GO:0008360 syntaxin interacting protein 1 | PREDICTED: probable protein BRICK1-B-like isoform X1 [Ceratitis capitata] | 1E-34 | - |
| TRINITY_DN17141_c1_g2_i1 | 1171 | 57 | - | 20,544 | GO:0016791 myotubularin-related protein 14 | PREDICTED: myotubularin-related protein 14 [Bactrocera cucurbitae] | 0.0 | gi\|498933124\|ref\|XP_004519564.1\| |
| TRINITY_DN17206_c0_g2_i2 | 225 | 15 | - | 15 | GO:0045167 serine threonine-protein kinase aurora-2 | PREDICTED: serine/threonine-protein kinase Aurora-2 [Bactrocera dorsalis] | 0 | gi\|751448568\|ref\|XP_011178471.1\| |
| TRINITY_DN16626_c0_g2_i3 | 221 | 12 | - | 18,417 | - - | PREDICTED: inositol-trisphosphate 3-kinase A [Bactrocera dorsalis] | 0.0 | gi\|751782425\|ref\|XP_011200416.1\| |
| TRINITY_DN17175_c0_g1_i1 | 219 | 12 | - | 18,25 | GO:0008298 a-kinase anchor protein mitochondrial | PREDICTED: A-kinase anchor protein 1, mitochondrial [Bactrocera dorsalis]<>PREDICTED: A-kinase anchor protein 1, mitochondrial [Bactrocera dorsalis]<>PREDICTED: A-kinase anchor protein 1, mitochondrial [Bactrocera dorsalis]<>PREDICTED: A-kinase anchor protein 1, mitochondrial [Bactrocera dorsalis] | 0.0 | gi\|751781714\|ref\|XP_011200035.1\| |
| TRINITY_DN15109_c0_g3_i5 | 208 | 14 | - | 14,857 | GO:0051301 cell division control protein 45 homolog | PREDICTED: cell division control protein 45 homolog [Bactrocera dorsalis] | 0 | gi\|751468010\|ref\|XP_011189115.1\| |
| TRINITY_DN15298_c0_g1_i1 | 212 | 13 | - | 16,308 | - - | PREDICTED: uncharacterized protein LOC101454622 [Ceratitis capitata] | 1E-121 | gi\|498945218\|ref\|XP_004522174.1\| |
| TRINITY_DN14538_c0_g2_i6 | 284 | 10 | - | 28,4 | - | PREDICTED: uncharacterized protein LOC105212477 [Bactrocera cucurbitae] | 8E-72 | gi\|498946430\|ref\|XP_004522370.1\| |
| TRINITY_DN14642_c0_g1_i2 | 137 | 11 | - | 12,455 | - - | PREDICTED: DNA-directed RNA polymerase I subunit RPA43 [Bactrocera dorsalis] | 1E-117 | gi\|751470876\|ref\|XP_011190689.1\| |
| TRINITY_DN15859_c0_g2_i4 | 124 | 10 | - | 12,4 | GO:0050660 alkyldihydroxyacetonephosphate synthase | PREDICTED: alkyldihydroxyacetonephosphate synthase isoform X1 [Bactrocera cucurbitae]<>PREDICTED: alkyldihydroxyacetonephosphate synthase isoform X2 [Bactrocera cucurbitae] | 0 | gi\|751793150\|ref\|XP_011206248.1\| |
| TRINITY_DN12504_c0_g1_i1 | 766 | 20 | - | 38,3 | GO:0048812 fas apoptotic inhibitory molecule 1 | PREDICTED: fas apoptotic inhibitory molecule 1 [Bactrocera dorsalis] | 2E-117 | gi\|751442117\|ref\|XP_011195684.1\| |
| TRINITY_DN16427_c1_g3_i4 | 292 | 12 | - | 24,333 | GO:0002027 protein nanos | PREDICTED: protein nanos [Bactrocera cucurbitae] | 2E-85 | gi\|751472768\|ref\|XP_011191713.1\| |
| TRINITY_DN13566_c0_g1_i2 | 342 | 18 | - | 19 | GO:0003779 tropomyosin isoform b | PREDICTED: tropomyosin-2 [Bactrocera dorsalis] | 1E-105 | gi\|751780564\|ref\|XP_011199409.1\| |
| TRINITY_DN11053_c0_g1_i6 | 141 | 11 | - | 12,818 | - - | PREDICTED: pre-rRNA-processing protein TSR2 homolog [Ceratitis capitata] | 2E-69 | gi\|751797114\|ref\|XP_011208407.1\| |
| TRINITY_DN12780_c0_g2_i2 | 335 | 14 | - | 23,929 | GO:0032133 borealin-like isoform x1 | PREDICTED: borealin-like [Ceratitis capitata] | 5E-111 | gi\|751458235\|ref\|XP_011183776.1\| |
| TRINITY_DN15695_c0_g1_i3 | 172 | 13 | - | 13,231 | GO:0016491 dehydrogenase reductase sdr family member 11-like | PREDICTED: dehydrogenase/reductase SDR family member 11-like [Bactrocera dorsalis] | 7E-106 | gi\|751769598\|ref\|XP_011197958.1\| |
| TRINITY_DN16802_c0_g2_i2 | 203 | 12 | - | 16,917 | - - | PREDICTED: POC1 centriolar protein homolog [Ceratitis capitata] | 0 | gi\|751443308\|ref\|XP_011196342.1\| |
| TRINITY_DN12575_c0_g1_i1 | 25035 | 10 | - | 2503,5 | - | PREDICTED: microfibril-associated glycoprotein 4-like [Bactrocera cucurbitae] | 2E-103 | gi\|751778242\|ref\|XP_011198157.1\| |
| TRINITY_DN13637_c0_g1_i2 | 693 | 30 | - | 23,1 | - - | PREDICTED: cell death abnormality protein 1 isoform X1 [Bactrocera cucurbitae] | 8E-101 | gi\|499010487\|ref\|XP_004536815.1\| |
| TRINITY_DN16583_c0_g1_i1 | 279 | 11 | - | 25,364 | GO:0048468 otefin | PREDICTED: otefin [Bactrocera cucurbitae] | 1E-121 | gi\|751471283\|ref\|XP_011190910.1\| |
| TRINITY_DN10659_c0_g1_i2 | 1576 | 40 | - | 39,4 | GO:0031987 isoform a | PREDICTED: calreticulin [Bactrocera cucurbitae] | 2E-138 | gi\|751780461\|ref\|XP_011199354.1\| |
| TRINITY_DN12527_c0_g1_i1 | 10832 | 14 | - | 773,71 | - | PREDICTED: maltase 2-like [Bactrocera cucurbitae] | 0.0 | gi\|751780564\|ref\|XP_011199409.1\| |
| TRINITY_DN13940_c0_g1_i3 | 810 | 35 | - | 23,143 | - | PREDICTED: nucleolar protein 58 [Bactrocera cucurbitae] | 0.0 | gi\|751771188\|ref\|XP_011203797.1\| |
| TRINITY_DN16390_c0_g1_i1 | 519 | 13 | - | 39,923 | GO:0003676 protein argonaute-3 | PREDICTED: piwi-like protein 1-like [Ceratitis capitata] | 0.0 | gi\|751803381\|ref\|XP_011211828.1\| |
| TRINITY_DN15777_c0_g1_i5 | 241 | 18 | - | 13,389 | GO:0006468 cell division control protein 2 cognate | PREDICTED: cell division control protein 2 cognate [Bactrocera cucurbitae] | 1E-154 | gi\|498959369\|ref\|XP_004524465.1\| |
| TRINITY_DN17291_c0_g3_i3 | 541 | 15 | - | 36,067 | GO:0007067 g2 mitotic-specific cyclin-b3 | PREDICTED: G2/mitotic-specific cyclin-B3 [Bactrocera cucurbitae] | 3E-180 | gi\|751456418\|ref\|XP_011182776.1\| |
| TRINITY_DN17606_c0_g5_i1 | 265 | 12 | - | 22,083 | GO:0044763 uncharacterized protein loc105231070 | PREDICTED: uncharacterized protein LOC105218818 [Bactrocera cucurbitae] | 0.0 | gi\|751774761\|ref\|XP_011214014.1\| |
| TRINITY_DN12957_c0_g2_i1 | 235 | 13 | - | 18,077 | GO:0003746 probable elongation factor 1-delta isoform x1 | PREDICTED: probable elongation factor 1-delta isoform X1 [Bactrocera dorsalis] | 6E-122 | gi\|751465831\|ref\|XP_011187920.1\| |
| TRINITY_DN13879_c0_g1_i2 | 110 | 11 | - | 10 |  | PREDICTED: uncharacterized protein LOC105227399 isoform X2 [Bactrocera dorsalis] | 0.0 | gi\|751793718\|ref\|XP_011206548.1\| |
| TRINITY_DN14198_c3_g11_i1 | 351 | 21 | - | 16,714 | - - | PREDICTED: heat shock protein 23-like [Bactrocera cucurbitae] | 4E-69 | gi\|751462154\|ref\|XP_011185915.1\| |
| TRINITY_DN12288_c0_g1_i2 | 880 | 10 | - | 88 | GO:0051298 tubulin alpha-4 chain | PREDICTED: tubulin alpha-4 chain [Bactrocera dorsalis] | 0.0 | gi\|751788691\|ref\|XP_011203818.1\| |
| TRINITY_DN13521_c0_g1_i5 | 152 | 11 | - | 13,818 | - - | PREDICTED: U3 small nucleolar RNA-associated protein 18 homolog [Bactrocera cucurbitae] | 0 | gi\|498933309\|ref\|XP_004519602.1\| |
| TRINITY_DN17085_c0_g1_i1 | 191 | 11 | - | 17,364 | GO:0003678 dna replication licensing factor mcm6 | PREDICTED: DNA replication licensing factor Mcm6 [Bactrocera dorsalis] | 0 | gi\|498982341\|ref\|XP_004529836.1\| |
| TRINITY_DN17299_c0_g3_i1 | 440 | 20 | - | 22 | - - | PREDICTED: uncharacterized protein LOC101460126 [Ceratitis capitata] | 1E-170 | gi\|751792187\|ref\|XP_011205722.1\| |
| TRINITY_DN16314_c0_g1_i1 | 360 | 13 | - | 27,692 | GO:0051298 protein regulator of cytokinesis 1-like | PREDICTED: protein regulator of cytokinesis 1-like [Bactrocera cucurbitae] | 0.0 | gi\|498945351\|ref\|XP_004522196.1\| |
| TRINITY_DN11563_c0_g2_i2 | 1184 | 76 | - | 15,579 | GO:0090328 atp-dependent rna helicase me31b | PREDICTED: putative ATP-dependent RNA helicase me31b-like [Ceratitis capitata] | 0 | gi\|751468753\|ref\|XP_011189523.1\| |
| TRINITY_DN15493_c0_g4_i1 | 272 | 16 | - | 17 | GO:0006772 thiamin pyrophosphokinase 1-like isoform x1 | PREDICTED: thiamin pyrophosphokinase 1-like isoform X1 [Ceratitis capitata] | 2E-95 | gi\|751462398\|ref\|XP_011186047.1\| |
| TRINITY_DN17304_c0_g1_i1 | 1665 | 57 | - | 29,211 | GO:0042246 ribonucleoside-diphosphate reductase large subunit-like | PREDICTED: ribonucleoside-diphosphate reductase large subunit [Bactrocera cucurbitae] | 0.0 | gi\|751456747\|ref\|XP_011182959.1\| |
| TRINITY_DN13273_c0_g1_i1 | 146 | 10 | - | 14,6 | GO:0005875 t-complex polypeptide 20 | PREDICTED: T-complex protein 1 subunit zeta-like [Ceratitis capitata] | 0 | gi\|751466348\|ref\|XP_011188204.1\| |
| TRINITY_DN15599_c0_g3_i2 | 223 | 12 | - | 18,583 | GO:0005737 proteasome subunit alpha type-6-like | PREDICTED: proteasome subunit alpha type-6 [Bactrocera dorsalis] | 5E-124 | gi\|751448853\|ref\|XP_011178629.1\| |
| TRINITY_DN15818_c0_g4_i1 | 154 | 10 | - | 15,4 | - - | PREDICTED: uncharacterized protein LOC105211869 [Bactrocera cucurbitae] | 1E-77 | gi\|751470880\|ref\|XP_011190691.1\| |
| TRINITY_DN14392_c0_g2_i7 | 233 | 11 | - | 21,182 | GO:0006270 dna replication licensing factor mcm4 | PREDICTED: DNA replication licensing factor MCM4 [Bactrocera dorsalis] | 0.0 | gi\|499009467\|ref\|XP_004536573.1\| |
| TRINITY_DN14784_c0_g3_i1 | 406 | 23 | - | 17,652 | GO:0032508 dna replication licensing factor mcm5-like | PREDICTED: DNA replication licensing factor Mcm5-like [Ceratitis capitata] | 0 | gi\|751467213\|ref\|XP_011188678.1\| |
| TRINITY_DN17348_c0_g4_i3 | 534 | 23 | - | 23,217 | GO:0035392 rap1 interacting factor 1 homolog | PREDICTED: uncharacterized protein LOC101453155 [Ceratitis capitata] | 0.0 | gi\|751474932\|ref\|XP_011192884.1\| |
| TRINITY_DN15178_c1_g7_i1 | 293 | 16 | - | 18,313 | - - | PREDICTED: lipopolysaccharide-induced tumor necrosis factor-alpha factor-like [Ceratitis capitata] | 6E-19 | gi\|751475052\|ref\|XP_011192950.1\| |
| TRINITY_DN12527_c0_g1_i2 | 7149 | 15 | - | 476,6 | - | PREDICTED: maltase 2-like [Bactrocera cucurbitae] | 0.0 | gi\|751774727\|ref\|XP_011213995.1\| |
| TRINITY_DN16639_c0_g5_i1 | 269 | 13 | - | 20,692 | GO:0007144 cell division cycle protein 20 homolog | PREDICTED: cell division cycle protein 20 homolog [Ceratitis capitata] | 0.0 | gi\|751790903\|ref\|XP_011205025.1\| |
| TRINITY_DN17744_c1_g1_i1 | 325 | 13 | - | 25 | GO:0005829 eukaryotic translation initiation factor 2-alpha kinase 4 | PREDICTED: eukaryotic translation initiation factor 2-alpha kinase 4 [Bactrocera cucurbitae] | 0.0 | gi\|751442109\|ref\|XP_011195679.1\| |
| TRINITY_DN16239_c0_g3_i2 | 125 | 10 | - | 12,5 | - - | PREDICTED: nuclear pore complex protein Nup155 [Bactrocera cucurbitae] | 0 | gi\|751804925\|ref\|XP_011212670.1\| |
| TRINITY_DN13926_c1_g1_i5 | 546 | 27 | - | 20,222 | GO:0009982 h aca ribonucleoprotein complex subunit 4 | PREDICTED: H/ACA ribonucleoprotein complex subunit 4 isoform X2 [Bactrocera cucurbitae] | 0.0 | gi\|751469783\|ref\|XP_011190093.1\| |
| TRINITY_DN14948_c0_g16_i1 | 20264 | 25 | - | 810,56 | GO:0005576 yolk partial | yolk polypeptide, partial [Anastrepha suspensa] | 2E-105 | gi\|751780339\|ref\|XP_011199288.1\| |
| TRINITY_DN16810_c0_g1_i7 | 151 | 10 | - | 15,1 | - - | PREDICTED: uncharacterized protein LOC105221078 [Bactrocera cucurbitae] | 0 | gi\|807027295\|ref\|XP_012156878.1\| |
| TRINITY_DN16566_c0_g2_i1 | 1057 | 18 | - | 58,722 | GO:0009987 isoform a | PREDICTED: insulin receptor [Bactrocera cucurbitae] | 0.0 | gi\|751464664\|ref\|XP_011187285.1\| |
| TRINITY_DN13268_c0_g2_i1 | 2670 | 122 | - | 21,885 | - - | PREDICTED: sarcocystatin-A-like [Bactrocera dorsalis] | 9E-23 | gi\|498957530\|ref\|XP_004524166.1\| |
| TRINITY_DN17694_c1_g3_i1 | 264 | 10 | - | 26,4 | GO:0006355 myc protein | PREDICTED: myc protein [Bactrocera cucurbitae]<>PREDICTED: myc protein [Bactrocera cucurbitae] | 4E-45 | gi\|498956619\|ref\|XP_004524023.1\| |
| TRINITY_DN15700_c0_g3_i4 | 286 | 19 | - | 15,053 | GO:0004170 deoxyuridine 5 -triphosphate nucleotidohydrolase isoform x1 | PREDICTED: deoxyuridine 5'-triphosphate nucleotidohydrolase isoform X1 [Bactrocera dorsalis] | 3E-81 | gi\|751463426\|ref\|XP_011186612.1\| |
| TRINITY_DN11233_c0_g1_i3 | 19 | 364 | - | 0,0522 | GO:0015991 v-type proton atpase subunit e-like | PREDICTED: V-type proton ATPase subunit e [Bactrocera dorsalis] | 1E-43 | gi\|751453841\|ref\|XP_011181364.1\| |
| TRINITY_DN9842_c0_g1_i2 | 17 | 826 | - | 0,0206 | GO:0006413 GO:0003743 | PREDICTED: protein translation factor SUI1 homolog isoform X1 [Ceratitis capitata]<>PREDICTED: protein translation factor SUI1 homolog [Bactrocera cucurbitae]<>PREDICTED: protein translation factor SUI1 homolog [Bactrocera dorsalis] | 8E-51 | gi\|780635304\|ref\|XP_011686504.1\| |
| TRINITY_DN14777_c0_g2_i1 | 22 | 472 | - | 0,0466 | - - | PREDICTED: thioredoxin domain-containing protein 17 isoform X2 [Bactrocera cucurbitae] | 4E-68 | gi\|498933317\|ref\|XP_004519606.1\| |
| TRINITY_DN9463_c0_g3_i2 | 21 | 333 | - | 0,0631 | - - | PREDICTED: uncharacterized protein LOC105212662 [Bactrocera cucurbitae] | 5E-31 | gi\|751782806\|ref\|XP_011200623.1\| |
| TRINITY_DN16767_c1_g1_i3 | 10 | 160 | - | 0,0625 | GO:0020037 cytochrome b5-like | PREDICTED: cytochrome b5 [Bactrocera cucurbitae] | 2E-39 | gi\|751789628\|ref\|XP_011204332.1\| |
| TRINITY_DN11845_c0_g1_i1 | 11 | 258 | - | 0,0426 | - - | PREDICTED: sapecin-like [Bactrocera cucurbitae] | 1E-36 | gi\|751473093\|ref\|XP_011191886.1\| |
| TRINITY_DN12145_c0_g1_i3 | 16 | 619 | - | 0,0258 | - - | unnamed protein product [Schistosoma mansoni] | 2E-17 | gi\|751457518\|ref\|XP_011183381.1\| |
| TRINITY_DN10546_c0_g1_i3 | 11 | 992 | - | 0,0111 | - | PREDICTED: uncharacterized protein LOC105212965 [Bactrocera cucurbitae] | 9E-26 | gi\|751441872\|ref\|XP_011195550.1\| |
| TRINITY_DN17307_c1_g1_i1 | 16 | 442 | - | 0,0362 | GO:0046961 atp synthase subunit mitochondrial-like isoform x2 | PREDICTED: ATP synthase subunit epsilon, mitochondrial-like isoform X2 [Ceratitis capitata] | 4E-21 | gi\|751797867\|ref\|XP_011208821.1\| |
| TRINITY_DN16930_c1_g1_i3 | 13 | 1821 | - | 0,0071 | GO:0008299 hydroxymethylglutaryl- synthase 1 | PREDICTED: hydroxymethylglutaryl-CoA synthase 1 [Bactrocera cucurbitae] | 0.0 | gi\|751788225\|ref\|XP_011203563.1\| |
| TRINITY_DN15916_c0_g4_i3 | 30 | 490 | - | 0,0612 | GO:0012505 androgen-induced gene 1 isoform x1 | PREDICTED: androgen-induced gene 1 protein isoform X2 [Bactrocera cucurbitae] | 7E-112 | gi\|499010187\|ref\|XP_004536740.1\| |
| TRINITY_DN15050_c0_g1_i1 | 43 | 3766 | - | 0,0114 | GO:0006694 cytochrome p450 4g15 | PREDICTED: cytochrome P450 4g15 [Bactrocera dorsalis] | 0.0 | gi\|751770899\|ref\|XP_011202111.1\| |
| TRINITY_DN15050_c0_g1_i4 | 34 | 3845 | - | 0,0088 | GO:0006694 cytochrome p450 4g15 | PREDICTED: cytochrome P450 4g15 [Bactrocera dorsalis] | 0.0 | gi\|751469916\|ref\|XP_011190165.1\| |
| TRINITY_DN9958_c0_g1_i1 | 20 | 337 | - | 0,0593 | GO:0055092 protein npc2 homolog | PREDICTED: protein NPC2 homolog [Ceratitis capitata] | 2E-76 | gi\|751458627\|ref\|XP_011183989.1\| |
| TRINITY_DN17727_c0_g2_i5 | 13 | 378 | - | 0,0344 | GO:0007280 3-hydroxy-3-methylglutaryl-coenzyme a reductase-like isoform x1 | PREDICTED: LOW QUALITY PROTEIN: 3-hydroxy-3-methylglutaryl-coenzyme A reductase [Bactrocera dorsalis] | 0 | gi\|498967743\|ref\|XP_004526141.1\| |
| TRINITY_DN17727_c0_g2_i6 | 13 | 422 | - | 0,0308 | GO:0007280 3-hydroxy-3-methylglutaryl-coenzyme a reductase-like isoform x1 | PREDICTED: 3-hydroxy-3-methylglutaryl-coenzyme A reductase [Bactrocera cucurbitae] | 0 | gi\|498939419\|ref\|XP_004521183.1\| |
| TRINITY_DN13360_c0_g1_i2 | 10 | 317 | - | 0,0315 | GO:0016021 peroxisomal membrane protein 2 | PREDICTED: peroxisomal membrane protein 2 [Bactrocera cucurbitae] | 3E-91 | gi\|195029339\|ref\|XP_001987531.1\| |
| TRINITY_DN12024_c0_g1_i3 | 74 | 3079 | - | 0,024 | - - | PREDICTED: alpha-tocopherol transfer protein-like [Ceratitis capitata] | 6,00E-144 | gi\|751455216\|ref\|XP_011182119.1\| |
| TRINITY_DN15425_c0_g1_i4 | 108 | 1843 | - | 0,0586 | GO:0035220 farnesyl pyrophosphate synthase | GJ15042 [Drosophila virilis]<>GJ15042 [Drosophila virilis] | 1E-145 | gi\|751796554\|ref\|XP_011208097.1\| |
| TRINITY_DN16444_c0_g2_i1 | 22 | 1267 | - | 0,0174 | GO:0008152 GO:0016787 | PREDICTED: abhydrolase domain-containing protein 3 [Bactrocera cucurbitae] | 0.0 | gi\|498934530\|ref\|XP_004519899.1\| |
| TRINITY_DN14438_c0_g1_i2 | 74 | 1216 | - | 0,0609 | GO:0008106 alcohol dehydrogenase | PREDICTED: alcohol dehydrogenase [NADP(+)]-like [Bactrocera cucurbitae] | 4E-151 | gi\|751473695\|ref\|XP_011192212.1\| |
| TRINITY_DN15413_c0_g1_i1 | 10 | 208 | - | 0,0481 | - - - | PREDICTED: uncharacterized protein LOC101459897 [Ceratitis capitata] | 5E-53 | gi\|751456985\|ref\|XP_011183088.1\| |
| TRINITY_DN13136_c0_g1_i5 | 76 | 1435 | - | 0,053 | - - | PREDICTED: elongation of very long chain fatty acids protein AAEL008004-like [Ceratitis capitata] | 5E-102 | gi\|751457754\|ref\|XP_011183512.1\| |
| TRINITY_DN15969_c0_g1_i4 | 11 | 240 | - | 0,0458 | - - | PREDICTED: serine-rich adhesin for platelets-like [Ceratitis capitata] | 2E-76 | gi\|751455414\|ref\|XP_011182230.1\| |
| TRINITY_DN15093_c0_g2_i2 | 38 | 1324 | - | 0,0287 | - - | PREDICTED: carbonic anhydrase 2-like [Bactrocera dorsalis] | 4E-109 | gi\|350646623\|emb\|CCD58743.1\| |
| TRINITY_DN15928_c1_g1_i1 | 241 | 4726 | - | 0,051 | - - | PREDICTED: vitellogenin-1-like [Ceratitis capitata] | 1E-157 | gi\|751439342\|ref\|XP_011183689.1\| |
| TRINITY_DN17361_c0_g6_i4 | 10 | 518 | - | 0,0193 | GO:0020037 GO:0016020 GO:0016705 GO:0004497 GO:0005506 GO:0009055 GO:0055114 GO:0043231 | PREDICTED: probable cytochrome P450 4s3-like [Ceratitis capitata] | 0.0 | gi\|498938775\|ref\|XP_004521016.1\| |
| TRINITY_DN14624_c0_g2_i1 | 25 | 502 | - | 0,0498 | - - - | PREDICTED: LYR motif-containing protein 5-like [Ceratitis capitata] | 1E-16 | gi\|751454133\|ref\|XP_011181523.1\| |
| TRINITY_DN14438_c0_g1_i1 | 22 | 575 | - | 0,0383 | GO:0008106 alcohol dehydrogenase | PREDICTED: alcohol dehydrogenase [NADP(+)]-like [Bactrocera cucurbitae] | 6E-151 | gi\|751464954\|ref\|XP_011187445.1\| |
| TRINITY_DN10750_c0_g1_i3 | 33 | 656 | - | 0,0503 | GO:0022008 uncharacterized protein loc105231623 isoform x2 | PREDICTED: uncharacterized protein LOC105221515 [Bactrocera cucurbitae] | 5E-176 | gi\|751782901\|ref\|XP_011200674.1\| |
| TRINITY_DN17452_c0_g3_i3 | 30 | 685 | - | 0,0438 | - - | PREDICTED: long-chain-fatty-acid--CoA ligase bubblegum-like [Bactrocera cucurbitae] | 0 | gi\|751782901\|ref\|XP_011200674.1\| |
| TRINITY_DN10750_c0_g1_i2 | 25 | 2297 | - | 0,0109 | GO:0022008 uncharacterized protein loc105231623 isoform x2 | PREDICTED: uncharacterized protein LOC105221515 [Bactrocera cucurbitae] | 5E-176 | gi\|499008638\|ref\|XP_004536369.1\| |
| TRINITY_DN11817_c0_g1_i4 | 31 | 607 | - | 0,0511 | GO:0016787 isopentenyl-diphosphate delta-isomerase 1 | PREDICTED: isopentenyl-diphosphate Delta-isomerase 1 [Bactrocera dorsalis]<>PREDICTED: isopentenyl-diphosphate Delta-isomerase 1 [Bactrocera dorsalis] | 9E-85 | gi\|751789156\|ref\|XP_011204072.1\| |
| TRINITY_DN17452_c0_g3_i1 | 22 | 763 | - | 0,0288 | - - | PREDICTED: long-chain-fatty-acid--CoA ligase bubblegum-like [Bactrocera cucurbitae] | 0 | gi\|751441025\|ref\|XP_011192789.1\| |
| TRINITY_DN13532_c0_g1_i7 | 12 | 381 | - | 0,0315 | -- - | PREDICTED: coagulation factor IX-like [Bactrocera cucurbitae] | 6E-50 | gi\|751456420\|ref\|XP_011182777.1\| |
| TRINITY_DN10750_c0_g1_i1 | 21 | 357 | - | 0,0588 | GO:0022008 uncharacterized protein loc105231623 isoform x2 | PREDICTED: uncharacterized protein LOC105221515 [Bactrocera cucurbitae] | 2E-172 | gi\|498926013\|ref\|XP_004517809.1\| |
| TRINITY_DN16050_c0_g3_i1 | 19 | 348 | - | 0,0546 | GO:0005875 acyl- synthetase family member mitochondrial-like | PREDICTED: acyl-CoA synthetase family member 2, mitochondrial-like [Bactrocera cucurbitae] | 0 | gi\|195402409\|ref\|XP_002059798.1\| |
| TRINITY_DN17665_c0_g9_i6 | 15 | 241 | - | 0,0622 | - - | PREDICTED: esterase B1-like isoform X2 [Ceratitis capitata] | 0 | gi\|751464192\|ref\|XP_011187031.1\| |
| TRINITY_DN15050_c0_g1_i2 | 58 | 1920 | - | 0,0302 | GO:0006694 cytochrome p450 4g15 | PREDICTED: cytochrome P450 4g15 [Bactrocera dorsalis] | 2E-162 | gi\|751467978\|ref\|XP_011189098.1\| |
| TRINITY_DN13532_c0_g1_i6 | 12 | 493 | - | 0,0243 | - - | PREDICTED: coagulation factor IX-like [Bactrocera cucurbitae] | 4E-50 | gi\|498962543\|ref\|XP_004525027.1\| |
| TRINITY_DN13991_c0_g4_i3 | 15 | 279 | - | 0,0538 | - - | PREDICTED: uncharacterized protein LOC105232379 [Bactrocera dorsalis] | 9E-51 | gi\|498996727\|ref\|XP_004533427.1\| |
| TRINITY_DN17361_c0_g6_i3 | 19 | 309 | - | 0,0615 | GO:0020037 probable cytochrome p450 4s3-like | PREDICTED: probable cytochrome P450 4s3-like [Ceratitis capitata] | 0 | gi\|498964269\|ref\|XP_004525363.1\| |
| TRINITY_DN16502_c0_g1_i4 | 40 | 1120 | - | 0,0357 | GO:0008146 sulfotransferase 1 family member d1 isoform x2 | PREDICTED: sulfotransferase 1 family member D1 isoform X2 [Bactrocera cucurbitae] | 4E-110 | gi\|751788225\|ref\|XP_011203563.1\| |
| TRINITY_DN15085_c0_g3_i2 | 30 | 898 | - | 0,0334 | GO:0005549 odorant binding protein 1 | odorant binding protein 1 [Bactrocera dorsalis] | 2E-30 | gi\|499004553\|ref\|XP_004535360.1\| |
| TRINITY_DN15179_c2_g8_i1 | 364 | 6983 | - | 0,0521 | - - | PREDICTED: general odorant-binding protein 19d-like [Bactrocera cucurbitae] | 2E-15 | gi\|498962294\|ref\|XP_004524976.1\| |
| TRINITY_DN16992_c0_g3_i3 | 31 | 555 | - | 0,0559 | - - | PREDICTED: uncharacterized protein LOC101460602 [Ceratitis capitata] | 2E-92 | gi\|499014477\|ref\|XP_004537792.1\| |
| TRINITY_DN13095_c0_g5_i1 | 14 | 457 | - | 0,0306 | - | PREDICTED: uncharacterized protein LOC101455903 [Ceratitis capitata] | 1E-30 | gi\|751467978\|ref\|XP_011189098.1\| |
| TRINITY_DN15490_c0_g2_i1 | 23 | 869 | - | 0,0265 | GO:0008152 microsomal glutathione s-transferase 1 | PREDICTED: microsomal glutathione S-transferase 1 [Bactrocera dorsalis] | 1E-36 | gi\|751444234\|ref\|XP_011196851.1\| |
| TRINITY_DN17373_c0_g3_i4 | 10 | 169 | - | 0,0592 | - - | PREDICTED: leukocyte elastase inhibitor-like isoform X1 [Bactrocera cucurbitae] | 2E-96 | gi\|751453649\|ref\|XP_011181257.1\| |
| TRINITY_DN14365_c0_g5_i2 | 20 | 354 | - | 0,0565 | - - | PREDICTED: protein rolling stone [Bactrocera cucurbitae] | 1E-18 | gi\|751444234\|ref\|XP_011196851.1\| |
| TRINITY_DN12498_c0_g3_i1 | 2052 | 33903 | - | 0,0605 | GO:0005549 odorant-binding protein 99b | PREDICTED: general odorant-binding protein 99b-like [Ceratitis capitata] | 1E-29 | gi\|751779905\|ref\|XP_011199056.1\| |
| TRINITY_DN15179_c2_g3_i1 | 978 | 19106 | - | 0,0512 | - - | PREDICTED: general odorant-binding protein 19d-like [Bactrocera dorsalis] | 2E-15 | gi\|751453649\|ref\|XP_011181257.1\| |
| TRINITY_DN17587_c1_g2_i1 | - | 27 | 807 | 29,889 | - | PREDICTED: ATP-sensitive inward rectifier potassium channel 12-like isoform X3 [Ceratitis capitata] | 0.0 | gi\|751464593\|ref\|XP_011187246.1\| |
| TRINITY_DN17733_c1_g1_i2 | - | 72 | 2696 | 37,444 | GO:0008236 GO:0016020 GO:0006508 | PREDICTED: venom dipeptidyl peptidase 4-like isoform X2 [Ceratitis capitata] | 0.0 | gi\|498955842\|ref\|XP_004523896.1\| |
| TRINITY_DN13225_c0_g1_i2 | - | 23 | 1482 | 64,435 | GO:0019013 GO:0003676 GO:0030529 GO:0000166 | PREDICTED: heterogeneous nuclear ribonucleoprotein 27C [Bactrocera cucurbitae]<>PREDICTED: heterogeneous nuclear ribonucleoprotein 27C [Bactrocera cucurbitae]<>PREDICTED: heterogeneous nuclear ribonucleoprotein 27C [Bactrocera dorsalis]<>PREDICTED: heterogeneous nuclear ribonucleoprotein 27C [Bactrocera dorsalis] | 2E-176 | gi\|498942252\|ref\|XP_004521685.1\| |
| TRINITY_DN10186_c0_g1_i4 | - | 14 | 7463 | 533,07 | GO:0005875 GO:0008340 GO:0042026 GO:0042595 GO:0042742 GO:0007476 GO:0009408 GO:0005515 GO:0005737 GO:0050832 GO:0005634 heat shock protein 27 | PREDICTED: heat shock protein 27 [Bactrocera cucurbitae] | 9E-84 | gi\|751782981\|ref\|XP_011200720.1\| |
| TRINITY_DN17264_c0_g1_i4 | - | 63 | 2329 | 36,968 | GO:0005638 GO:0005637 GO:0005198 | PREDICTED: lamin Dm0-like isoform X1 [Ceratitis capitata]<>PREDICTED: lamin Dm0-like isoform X2 [Ceratitis capitata]<>PREDICTED: lamin Dm0-like isoform X3 [Ceratitis capitata]<>PREDICTED: lamin Dm0-like isoform X4 [Ceratitis capitata] | 0.0 | gi\|751456849\|ref\|XP_011183014.1\| |
| TRINITY_DN13129_c0_g1_i1 | - | 23 | 1445 | 62,826 | - | PREDICTED: gametocyte-specific factor 1-like [Bactrocera cucurbitae] | 1E-46 | gi\|751468390\|ref\|XP_011189324.1\| |
| TRINITY_DN12954_c0_g1_i1 | - | 57 | 5118 | 89,789 | GO:0007049g2 mitotic-specific cyclin-b | PREDICTED: G2/mitotic-specific cyclin-B [Bactrocera dorsalis] | 0.0 | gi\|498939419\|ref\|XP_004521183.1\| |
| TRINITY_DN14806_c0_g1_i3 | - | 21 | 593 | 28,238 | - | PREDICTED: modifier of mdg4 isoform X19 [Ceratitis capitata] | 4E-159 | gi\|751772526\|ref\|XP_011211346.1\| |
| TRINITY_DN17141_c1_g2_i1 | - | 57 | 3091 | 54,228 | GO:0016791 GO:0006470 GO:0048477 | PREDICTED: myotubularin-related protein 14 [Bactrocera cucurbitae] | 0.0 | - |
| TRINITY_DN10357_c0_g1_i1 | - | 31 | 2643 | 85,258 | GO:0000786 GO:0051298 GO:0003677 GO:0006334 GO:0046982 GO:0005811 GO:0005634 | PREDICTED: histone H2A-like [Linepithema humile] | 1E-52 | - |
| TRINITY_DN17040_c0_g1_i1 | - | 33 | 1188 | 36 | - | PREDICTED: regulator of chromosome condensation [Bactrocera cucurbitae]<>PREDICTED: regulator of chromosome condensation [Bactrocera cucurbitae]<>PREDICTED: regulator of chromosome condensation [Bactrocera cucurbitae] | 0.0 | - |
| TRINITY_DN12527_c0_g1_i1 | - | 14 | 18302 | 1307,3 | - | PREDICTED: maltase 2-like [Bactrocera cucurbitae] | 0.0 | - |
| TRINITY_DN17506_c0_g4_i1 | - | 41 | 1229 | 29,976 | GO:0046331 GO:0005515 GO:0016538 GO:0035186 GO:0045841 GO:0045170 GO:0007096 GO:0045169 GO:0007422 GO:0035561 GO:0009794 GO:0005634 GO:0055059 | PREDICTED: G2/mitotic-specific cyclin-A [Bactrocera cucurbitae]<>PREDICTED: G2/mitotic-specific cyclin-A [Bactrocera cucurbitae] | 0.0 | - |
| TRINITY_DN14538_c0_g2_i6 | - | 10 | 667 | 66,7 | - | PREDICTED: uncharacterized protein LOC105212477 [Bactrocera cucurbitae] | 8E-72 | - |
| TRINITY_DN12504_c0_g1_i1 | - | 20 | 1351 | 67,55 | GO:0048812 GO:0043066 | PREDICTED: fas apoptotic inhibitory molecule 1 [Bactrocera dorsalis] | 2E-117 | - |
| TRINITY_DN16427_c1_g3_i4 | - | 12 | 432 | 36 | GO:0002027 GO:0042078 GO:0016321 GO:0048813 GO:0005506 GO:0005777 GO:0007444 GO:0007314 GO:0001709 GO:0006809 GO:0007416 GO:0007263 GO:0050661 GO:0008156 GO:0045892 GO:0006952 GO:0005829 GO:0020037 GO:0050660 GO:0007283 GO:0017148 GO:0010181 GO:0008270 GO:0055114 GO:0003958 GO:0003723 GO:0008285 GO:0005516 GO:0046620 GO:0004517 GO:0045495 GO:0031284 GO:0048134 GO:0006378 GO:0043066 GO:0007280 GO:0030718 | PREDICTED: protein nanos [Bactrocera cucurbitae] | 2E-85 | - |
| TRINITY_DN10186_c0_g1_i5 | - | 19 | 1492 | 78,526 | GO:0005875 GO:0008340 GO:0042026 GO:0042595 GO:0042742 GO:0007476 GO:0009408 GO:0005515 GO:0005737 GO:0050832 GO:0005634 | PREDICTED: heat shock protein 27 [Bactrocera cucurbitae] | 2E-43 | - |
| TRINITY_DN12780_c0_g2_i2 | - | 14 | 826 | 59 | GO:0032133 GO:1990385 GO:0090307 GO:0016572 GO:0003677 GO:0005938 GO:0000776 GO:0000785 GO:0005515 GO:0000281 GO:0045448 GO:0008104 | PREDICTED: borealin-like [Ceratitis capitata] | 5E-111 | - |
| TRINITY_DN14537_c0_g1_i1 | - | 17 | 480 | 28,235 | GO:0046579 | PREDICTED: uncharacterized protein LOC105219525 isoform X1 [Bactrocera cucurbitae] | 1E-80 | - |
| TRINITY_DN16802_c0_g2_i2 | - | 12 | 391 | 32,583 | - | PREDICTED: POC1 centriolar protein homolog [Ceratitis capitata] | 0.0 | - |
| TRINITY_DN12575_c0_g1_i1 | - | 10 | 23838 | 2383,8 | - | PREDICTED: microfibril-associated glycoprotein 4-like [Bactrocera cucurbitae] | 2E-103 | - |
| TRINITY_DN14806_c0_g1_i1 | - | 30 | 835 | 27,833 | - | PREDICTED: modifier of mdg4 isoform X3 [Bactrocera dorsalis] | 0.0 | - |
| TRINITY_DN13637_c0_g1_i2 | - | 30 | 1456 | 48,533 | - | PREDICTED: cell death abnormality protein 1 isoform X1 [Bactrocera cucurbitae] | 8E-101 | - |
| TRINITY_DN16583_c0_g1_i1 | - | 11 | 568 | 51,636 | GO:0048468 GO:0044428 GO:0050794 GO:0012505 GO:0032501 | PREDICTED: otefin [Bactrocera cucurbitae] | 1E-121 | - |
| TRINITY_DN17721_c1_g2_i2 | - | 18 | 1058 | 58,778 | - | PREDICTED: mucin-4 [Bactrocera cucurbitae] | 0.0 | - |
| TRINITY_DN13630_c0_g2_i3 | - | 13 | 371 | 28,538 | GO:0008152 GO:0003777 GO:0005871 | PREDICTED: kinesin light chain-like isoform X3 [Ceratitis capitata] | 0.0 | - |
| TRINITY_DN16390_c0_g1_i1 | - | 13 | 480 | 36,923 | GO:0003676 | PREDICTED: piwi-like protein 1-like [Ceratitis capitata] | 0.0 | - |
| TRINITY_DN13940_c0_g1_i3 | - | 35 | 1098 | 31,371 | - | PREDICTED: nucleolar protein 58 [Bactrocera cucurbitae] | 0.0 | - |
| TRINITY_DN13678_c0_g1_i1 | - | 114 | 14405 | 126,36 | GO:0005524 heat shock protein 70 | heat shock protein 70 [Bactrocera correcta] | 0.0 | - |
| TRINITY_DN15264_c0_g2_i1 | - | 22 | 702 | 31,909 | GO:0032508 GO:0006310 GO:0006281 GO:0005739 GO:0000723 GO:0000002 GO:0003677 GO:0005524 GO:0043141 GO:0005634 | PREDICTED: ATP-dependent DNA helicase PIF1 [Bactrocera cucurbitae] | 0.0 | - |
| TRINITY_DN17291_c0_g3_i3 | - | 15 | 1432 | 95,467 | GO:0007067 GO:0010389 GO:0000079 GO:0035561 GO:0007052 GO:0000281 GO:0019901 GO:0035186 GO:0016538 GO:0005634 g2 mitotic-specific cyclin-b3 | PREDICTED: G2/mitotic-specific cyclin-B3 [Bactrocera cucurbitae] | 3E-180 | - |
| TRINITY_DN17593_c0_g5_i1 | - | 21 | 1359 | 64,714 | GO:0009987 | PREDICTED: lateral signaling target protein 2 homolog [Bactrocera dorsalis] | 5E-176 | - |
| TRINITY_DN17606_c0_g5_i1 | - | 12 | 624 | 52 | GO:0044763 GO:0044424 GO:0050794 | PREDICTED: uncharacterized protein LOC105218818 [Bactrocera cucurbitae] | 0.0 | - |
| TRINITY_DN14198_c3_g11_i1 | - | 21 | 5225 | 248,81 | - | PREDICTED: heat shock protein 23-like [Bactrocera cucurbitae] | 4E-69 | - |
| TRINITY_DN12288_c0_g1_i2 | - | 10 | 1596 | 159,6 | GO:0051298 GO:0007417 GO:0035046 GO:0045298 GO:0007052 GO:0040016 GO:0005525 GO:0006184 GO:0005737 GO:0005874 GO:0051258 GO:0009792 GO:0007422 GO:0035038 GO:0022008 GO:0003924 GO:0005886 GO:0000280 GO:0005634 GO:0005200 GO:0035044 tubulin alpha-4 chain | PREDICTED: tubulin alpha-4 chain [Bactrocera dorsalis] | 0.0 | - |
| TRINITY_DN17085_c0_g1_i1 | - | 11 | 388 | 35,273 | GO:0003678 GO:0032508 GO:0007067 GO:0046872 GO:0042555 GO:0051301 GO:0003677 GO:0005524 GO:0006270 GO:0005634 | PREDICTED: DNA replication licensing factor Mcm6 [Bactrocera dorsalis] | 0.0 | - |
| TRINITY_DN16314_c0_g1_i1 | - | 13 | 567 | 43,615 | GO:0051298 GO:0051533 GO:0043148 GO:0005737 GO:0000916 GO:0051233 GO:0005813 GO:0000281 GO:0022008 GO:0030727 GO:0060429 | PREDICTED: protein regulator of cytokinesis 1-like [Bactrocera cucurbitae] | 0.0 | - |
| TRINITY_DN17642_c0_g1_i1 | - | 17 | 539 | 31,706 | GO:0007052 GO:0005813 GO:0044450 | PREDICTED: uncharacterized protein LOC105233683 [Bactrocera dorsalis] | 0.0 | - |
| TRINITY_DN15472_c0_g6_i1 | - | 49 | 2004 | 40,898 | - | PREDICTED: programmed cell death protein 4 [Bactrocera dorsalis] | 0.0 | - |
| TRINITY_DN17299_c0_g3_i6 | - | 21 | 686 | 32,667 | - | PREDICTED: uncharacterized protein LOC101460126 [Ceratitis capitata] | 1E-170 | - |
| TRINITY_DN17304_c0_g1_i1 | - | 57 | 2942 | 51,614 | GO:0042246 GO:0005971 GO:0006919 GO:0004748 GO:0055114 GO:0009263 GO:0005524 GO:0006260 | PREDICTED: ribonucleoside-diphosphate reductase large subunit [Bactrocera cucurbitae] | 0.0 | - |
| TRINITY_DN16414_c0_g1_i1 | - | 15 | 3065 | 204,33 | GO:0005524 heat shock protein 70 | heat shock protein 70 [Rhagoletis pomonella] | 0.0 | - |
| TRINITY_DN16414_c0_g1_i2 | - | 13 | 3806 | 292,77 | GO:0005524 heat shock protein 70 | heat shock protein 70 [Rhagoletis pomonella] | 0.0 | - |
| TRINITY_DN14392_c0_g2_i7 | - | 11 | 419 | 38,091 | GO:0006270 GO:0003677 GO:0032508 GO:0003678 GO:0005524 GO:0042555 | PREDICTED: DNA replication licensing factor MCM4 [Bactrocera dorsalis] | 0.0 | - |
| TRINITY_DN14784_c0_g3_i1 | - | 23 | 814 | 35,391 | GO:0032508 GO:0030261 GO:0000712 GO:0042555 GO:0043138 GO:0003677 GO:0005524 GO:0005656 GO:0006270 GO:0006267 GO:0042023 GO:0003682 | PREDICTED: DNA replication licensing factor Mcm5-like [Ceratitis capitata] | 0.0 | - |
| TRINITY_DN17348_c0_g4_i3 | - | 23 | 1223 | 53,174 | GO:0035392 GO:0005720 GO:0006974 | PREDICTED: uncharacterized protein LOC101453155 [Ceratitis capitata] | 0.0 | - |
| TRINITY_DN12527_c0_g1_i2 | - | 15 | 7161 | 477,4 | - | PREDICTED: maltase 2-like [Bactrocera cucurbitae] | 0.0 | - |
| TRINITY_DN16639_c0_g5_i1 | - | 13 | 461 | 35,462 | GO:0007144 GO:0008054 GO:0006909 GO:0007147 GO:0090302 GO:0030162 GO:0097150 GO:0005680 GO:0005737 GO:0007096 GO:0000776 GO:0005819 GO:0051488 GO:0005813GO:0030718 GO:0031145 GO:0022008 GO:0060547 | PREDICTED: cell division cycle protein 20 homolog [Ceratitis capitata] | 0.0 | - |
| TRINITY_DN17683_c0_g1_i3 | - | 13 | 411 | 31,615 | - | PREDICTED: flocculation protein FLO11-like isoform X1 [Bactrocera dorsalis]<>PREDICTED: flocculation protein FLO11-like isoform X1 [Bactrocera dorsalis] | 0.0 | - |
| TRINITY_DN17744_c1_g1_i1 | - | 13 | 674 | 51,846 | GO:0005829 GO:0006468 GO:0006417 GO:0071011 GO:0000398 GO:0045793 GO:0004686 GO:0000166 GO:0071013 | PREDICTED: eukaryotic translation initiation factor 2-alpha kinase 4 [Bactrocera cucurbitae]<>PREDICTED: eukaryotic translation initiation factor 2-alpha kinase 4 [Bactrocera cucurbitae] | 0.0 | - |
| TRINITY_DN15496_c0_g1_i4 | - | 12 | 392 | 32,667 | GO:0005852 GO:0016282 GO:0033290 GO:0006446 GO:0001731 GO:0003743 | PREDICTED: eukaryotic translation initiation factor 3 subunit D-1-like [Ceratitis capitata] | 0.0 | - |
| TRINITY_DN5005_c0_g2_i1 | - | 91 | 3328 | 36,571 | GO:0000022 GO:0051298 GO:0003735 GO:0022625 GO:0006412 | PREDICTED: 60S ribosomal protein L18a [Bactrocera dorsalis] | 8E-75 | - |
| TRINITY_DN16810_c0_g1_i7 | - | 10 | 336 | 33,6 | - | PREDICTED: uncharacterized protein LOC105221078 [Bactrocera cucurbitae]<>PREDICTED: uncharacterized protein LOC105221078 [Bactrocera cucurbitae]<>PREDICTED: uncharacterized protein LOC105221078 [Bactrocera cucurbitae]<>PREDICTED: uncharacterized protein LOC105221078 [Bactrocera cucurbitae] | 0.0 | - |
| TRINITY_DN16566_c0_g2_i1 | - | 18 | 1586 | 88,111 | GO:0009987 GO:0016740 | PREDICTED: insulin receptor [Bactrocera cucurbitae] | 0.0 | - |
| TRINITY_DN14198_c1_g1_i3 | - | 20 | 1194 | 59,7 | - | PREDICTED: heat shock protein 23-like [Bactrocera dorsalis] | 6E-62 | - |
| TRINITY_DN17235_c0_g9_i2 | - | 11 | 381 | 34,636 | GO:0008017 GO:0007018 GO:0005524 GO:0008152 GO:0005871 GO:0003777 GO:0005874 | PREDICTED: kinesin-like protein KIF23 [Bactrocera cucurbitae] | 0.0 | - |
| TRINITY_DN17694_c1_g3_i1 | - | 10 | 1015 | 101,5 | GO:0006355 GO:0003700 GO:0003677 GO:0046983 GO:0005634 myc protein | PREDICTED: myc protein [Bactrocera cucurbitae]<>PREDICTED: myc protein [Bactrocera cucurbitae] | 4E-45 | - |
| TRINITY_DN15700_c0_g3_i4 | - | 19 | 718 | 37,789 | GO:0004170 GO:0005515 GO:0046080 | PREDICTED: deoxyuridine 5'-triphosphate nucleotidohydrolase isoform X1 [Bactrocera dorsalis] | 3E-81 | - |
| TRINITY_DN16256_c0_g11_i1 | - | 12811 | 333 | 0,026 | GO:0000146 GO:0005859 GO:0005509 GO:0030016 GO:0003012 GO:0030239 GO:0060361 | PREDICTED: myosin regulatory light chain 2 [Bactrocera dorsalis] | 1E-43 | - |
| TRINITY_DN9228_c0_g2_i2 | - | 3744 | 107 | 0,0286 | GO:1902600 GO:0005739 GO:0055114 GO:0004129 | PREDICTED: cytochrome c oxidase subunit 6B2-like isoform X2 [Ceratitis capitata] | 7E-43 | - |
| TRINITY_DN10942_c0_g2_i3 | - | 1281 | 34 | 0,0265 | - | PREDICTED: male accessory gland serine protease inhibitor-like [Bactrocera dorsalis] | 2E-31 | - |
| TRINITY_DN14385_c0_g3_i1 | - | 601 | 16 | 0,0266 | GO:0034059 | PREDICTED: uncharacterized protein LOC105226290 isoform X2 [Bactrocera dorsalis] | 6E-36 | - |
| TRINITY_DN17210_c1_g1_i3 | - | 1648 | 55 | 0,0334 | - | PREDICTED: adhesive plaque matrix protein isoform X2 [Ceratitis capitata] | 1E-56 | - |
| TRINITY_DN11246_c0_g1_i1 | - | 436 | 11 | 0,0252 | GO:0005615 | PREDICTED: uncharacterized protein LOC105665460 [Ceratitis capitata] | 9E-58 | - |
| TRINITY_DN13143_c0_g2_i3 | - | 2907 | 53 | 0,0182 | GO:0006633 GO:0016717 GO:0016021 GO:0055114 | PREDICTED: acyl-CoA Delta(11) desaturase-like isoform X1 [Ceratitis capitata]<>PREDICTED: acyl-CoA Delta(11) desaturase-like isoform X2 [Ceratitis capitata]<>PREDICTED: acyl-CoA Delta(11) desaturase-like isoform X3 [Ceratitis capitata] | 0.0 | - |
| TRINITY_DN14178_c0_g1_i2 | - | 3604 | 108 | 0,03 | - | PREDICTED: troponin T, skeletal muscle isoform X9 [Bactrocera cucurbitae] | 4E-123 | - |
| TRINITY_DN12195_c0_g1_i1 | - | 1708 | 40 | 0,0234 | GO:0008152 GO:0016740 | PREDICTED: glutathione S-transferase 1-like [Bactrocera cucurbitae] | 5E-98 | - |
| TRINITY_DN16930_c1_g1_i3 | - | 1821 | 16 | 0,0088 | GO:0008299 GO:0004421 | PREDICTED: hydroxymethylglutaryl-CoA synthase 1 [Bactrocera cucurbitae]<>PREDICTED: hydroxymethylglutaryl-CoA synthase 1 [Bactrocera cucurbitae]<>PREDICTED: hydroxymethylglutaryl-CoA synthase 1 [Bactrocera cucurbitae]<>PREDICTED: hydroxymethylglutaryl-CoA synthase 1 [Bactrocera cucurbitae]<>PREDICTED: hydroxymethylglutaryl-CoA synthase 1 [Bactrocera cucurbitae]<>PREDICTED: hydroxymethylglutaryl-CoA synthase 1 [Bactrocera cucurbitae] | 0.0 | - |
| TRINITY_DN11957_c0_g2_i1 | - | 725 | 23 | 0,0317 | GO:0016021 GO:0006810 GO:0005215 | PREDICTED: aquaporin AQPAn.G-like isoform X3 [Bactrocera cucurbitae] | 3E-129 | - |
| TRINITY_DN17008_c1_g1_i2 | - | 348 | 13 | 0,0374 | GO:0005886 GO:0055085 GO:0015771 GO:0015574 GO:0016021 | PREDICTED: facilitated trehalose transporter Tret1-like isoform X3 [Ceratitis capitata] | 0.0 | - |
| TRINITY_DN16502_c0_g1_i2 | - | 1314 | 23 | 0,0175 | GO:0008146 GO:0008152 | PREDICTED: sulfotransferase 1 family member D1 isoform X2 [Bactrocera cucurbitae] | 4E-177 | - |
| TRINITY_DN12629_c0_g1_i2 | - | 1976 | 15 | 0,0076 | - | PREDICTED: uncharacterized protein LOC101453679 isoform X1 [Ceratitis capitata]<>PREDICTED: uncharacterized protein LOC101453679 isoform X2 [Ceratitis capitata] | 4E-63 | - |
| TRINITY_DN11027_c0_g1_i1 | - | 1372 | 10 | 0,0073 | - | PREDICTED: uncharacterized protein LOC101461967 [Ceratitis capitata] | 2E-89 | - |
| TRINITY_DN14889_c1_g1_i1 | - | 1589 | 19 | 0,012 | - | PREDICTED: uncharacterized protein LOC105234111 isoform X8 [Bactrocera dorsalis] | 3E-88 | - |
| TRINITY_DN16930_c1_g1_i4 | - | 547 | 14 | 0,0256 | GO:0008299 GO:0004421 | PREDICTED: hydroxymethylglutaryl-CoA synthase 1 [Bactrocera cucurbitae]<>PREDICTED: hydroxymethylglutaryl-CoA synthase 1 [Bactrocera cucurbitae]<>PREDICTED: hydroxymethylglutaryl-CoA synthase 1 [Bactrocera cucurbitae]<>PREDICTED: hydroxymethylglutaryl-CoA synthase 1 [Bactrocera cucurbitae]<>PREDICTED: hydroxymethylglutaryl-CoA synthase 1 [Bactrocera cucurbitae]<>PREDICTED: hydroxymethylglutaryl-CoA synthase 1 [Bactrocera cucurbitae] | 0.0 | - |
| TRINITY_DN9325_c0_g1_i1 | - | 3015 | 23 | 0,0076 | GO:0016021 GO:0055085 GO:0022857 synaptic vesicle glycoprotein 2b | PREDICTED: synaptic vesicle glycoprotein 2B [Bactrocera dorsalis]<>PREDICTED: synaptic vesicle glycoprotein 2B [Bactrocera dorsalis]<>PREDICTED: synaptic vesicle glycoprotein 2B [Bactrocera dorsalis]<>PREDICTED: synaptic vesicle glycoprotein 2B [Bactrocera dorsalis] | 0.0 | - |
| TRINITY_DN14004_c0_g1_i1 | - | 1057 | 28 | 0,0265 | GO:0006144 GO:0019628 GO:0005777 GO:0019428 GO:0004846 GO:0055114 | PREDICTED: uricase-like [Bactrocera cucurbitae] | 7E-171 | - |
| TRINITY_DN16287_c0_g1_i1 | - | 1636 | 31 | 0,0189 | GO:0004022 GO:0055114 | PREDICTED: alcohol dehydrogenase 2-like [Ceratitis capitata]<>alcohol dehydrogenase 2 [Ceratitis capitata] | 3E-131 | - |
| TRINITY_DN13462_c0_g2_i4 | - | 1962 | 26 | 0,0133 | GO:0020037 GO:0016705 GO:0004497 GO:0005506 GO:0055114 | PREDICTED: probable cytochrome P450 305a1 [Bactrocera dorsalis] | 0.0 | - |
| TRINITY_DN16444_c0_g2_i1 | - | 1267 | 18 | 0,0142 | GO:0008152 GO:0016787 | PREDICTED: abhydrolase domain-containing protein 3 [Bactrocera cucurbitae]<>PREDICTED: abhydrolase domain-containing protein 3 [Bactrocera cucurbitae] | 0.0 | - |
| TRINITY_DN16027_c2_g1_i11 | - | 299 | 11 | 0,0368 | GO:0016491 GO:0055114 | PREDICTED: dehydrogenase/reductase SDR family member 11-like [Bactrocera cucurbitae] | 4E-106 | - |
| TRINITY_DN14438_c0_g1_i2 | - | 1216 | 31 | 0,0255 | GO:0008106 GO:0035220 GO:0022416 GO:0055114 | PREDICTED: alcohol dehydrogenase [NADP(+)]-like [Bactrocera cucurbitae] | 4E-151 | - |
| TRINITY_DN14418_c0_g2_i1 | - | 1159 | 14 | 0,0121 | - | PREDICTED: uncharacterized protein LOC105215710 [Bactrocera cucurbitae] | 1E-31 | - |
| TRINITY_DN14382_c0_g1_i2 | - | 2173 | 45 | 0,0207 | GO:0006072 GO:0052591 GO:0005509 GO:0009331 GO:0055114 | PREDICTED: glycerol-3-phosphate dehydrogenase, mitochondrial isoform X1 [Bactrocera cucurbitae] | 0.0 | - |
| TRINITY_DN15404_c0_g1_i2 | - | 273 | 10 | 0,0366 | - | PREDICTED: trypsin-like [Ceratitis capitata] | 4E-76 | - |
| TRINITY_DN15560_c0_g1_i1 | - | 298 | 11 | 0,0369 | GO:0004568 GO:0007444 GO:0005975 GO:0006032 GO:0008084 GO:0005576 | PREDICTED: chitinase-like protein Idgf1-like [Ceratitis capitata] | 0.0 | - |
| TRINITY_DN14014_c0_g1_i1 | - | 515 | 15 | 0,0291 | GO:0006633 GO:0016717 GO:0005506 GO:0020037 GO:0055114 | PREDICTED: cytochrome b5-related protein [Bactrocera cucurbitae] | 0.0 | - |
| TRINITY_DN17361_c0_g6_i4 | - | 518 | 16 | 0,0309 | GO:0020037 GO:0016020 GO:0016705 GO:0004497 GO:0005506 GO:0009055 GO:0055114 GO:0043231 | PREDICTED: probable cytochrome P450 4s3-like [Ceratitis capitata] | 0.0 | - |
| TRINITY_DN17073_c0_g1_i2 | - | 919 | 23 | 0,025 | GO:0003779 GO:0003779 | PREDICTED: gelsolin-like [Ceratitis capitata] | 0.0 | - |
| TRINITY_DN16698_c0_g2_i2 | - | 1469 | 42 | 0,0286 | GO:0005737 GO:0004557 GO:0046477 GO:0009311 GO:0016139 | PREDICTED: alpha-N-acetylgalactosaminidase [Bactrocera cucurbitae]<>PREDICTED: alpha-N-acetylgalactosaminidase [Bactrocera cucurbitae] | 0.0 | - |
| TRINITY_DN10750_c0_g1_i2 | - | 2297 | 26 | 0,0113 | GO:0022008 | PREDICTED: uncharacterized protein LOC105221515 [Bactrocera cucurbitae] | 5E-176 | - |
| TRINITY_DN13718_c0_g1_i2 | - | 1165 | 17 | 0,0146 | GO:0005975 GO:0043169 GO:0003824 | PREDICTED: maltase A3 [Bactrocera dorsalis] | 0.0 | - |
| TRINITY_DN16797_c0_g1_i3 | - | 472 | 14 | 0,0297 | GO:0000166 GO:0051124 GO:0003730 GO:0007283 GO:0071011 GO:0008934 GO:0071013 GO:0043025 GO:0030424 GO:0048680 GO:0016311 GO:0016322 GO:0000398 GO:0046854 | PREDICTED: insulin-like growth factor 2 mRNA-binding protein 1 isoform X1 [Bactrocera cucurbitae] | 0.0 | - |
| TRINITY_DN16277_c0_g1_i2 | - | 448 | 14 | 0,0313 | - | PREDICTED: chorion peroxidase-like isoform X2 [Ceratitis capitata] | 0.0 | - |
| TRINITY_DN17361_c0_g6_i6 | - | 543 | 17 | 0,0313 | GO:0020037 GO:0016020 GO:0016705 GO:0004497 GO:0005506 GO:0009055 GO:0055114 GO:0043231 | PREDICTED: probable cytochrome P450 4s3-like [Ceratitis capitata] | 0.0 | - |
| TRINITY_DN10350_c0_g1_i5 | - | 3247 | 23 | 0,0071 | GO:0005388 GO:0016021 GO:0070588 GO:0046872 GO:0005524 GO:0008152 calcium-transporting atpase sarcoplasmic endoplasmic reticulum type isoform x1 | PREDICTED: calcium-transporting ATPase sarcoplasmic/endoplasmic reticulum type isoform X2 [Bactrocera dorsalis] | 0.0 | - |
| TRINITY_DN10750_c0_g1_i1 | - | 357 | 11 | 0,0308 | GO:0022008 | PREDICTED: uncharacterized protein LOC105221515 [Bactrocera cucurbitae] | 2E-172 | - |
| TRINITY_DN14062_c0_g1_i13 | - | 2057 | 11 | 0,0053 | GO:0046933 GO:0015991 GO:0045261 GO:0015986 GO:0005524 atp synthase subunit mitochondrial-like | PREDICTED: ATP synthase subunit beta, mitochondrial-like [Ceratitis capitata] | 0.0 | - |
| TRINITY_DN17224_c0_g3_i2 | - | 410 | 15 | 0,0366 | - | PREDICTED: endoplasmic reticulum metallopeptidase 1-like isoform X1 [Ceratitis capitata]<>PREDICTED: endoplasmic reticulum metallopeptidase 1-like isoform X2 [Ceratitis capitata]<>PREDICTED: endoplasmic reticulum metallopeptidase 1-like isoform X3 [Ceratitis capitata]<>PREDICTED: endoplasmic reticulum metallopeptidase 1-like isoform X4 [Ceratitis capitata] | 0.0 | - |
| TRINITY_DN17502_c0_g2_i1 | - | 1517 | 10 | 0,0066 | GO:0004177 | PREDICTED: membrane alanyl aminopeptidase [Bactrocera cucurbitae] | 0.0 | - |
| TRINITY_DN16050_c0_g3_i1 | - | 348 | 13 | 0,0374 | GO:0005875 | PREDICTED: acyl-CoA synthetase family member 2, mitochondrial-like [Bactrocera cucurbitae] | 0.0 | - |
| TRINITY_DN14062_c0_g1_i9 | - | 1230 | 21 | 0,0171 | GO:0046933 GO:0015991 GO:0045261 GO:0015986 GO:0005524 | PREDICTED: ATP synthase subunit beta, mitochondrial-like [Ceratitis capitata] | 0.0 | - |
| TRINITY_DN13560_c0_g1_i8 | - | 1681 | 10 | 0,0059 | - | PREDICTED: uncharacterized protein LOC105213980 isoform X3 [Bactrocera cucurbitae] | 3E-43 | - |
| TRINITY_DN16864_c0_g2_i2 | - | 363 | 12 | 0,0331 | GO:0035234 GO:0008028 GO:0007281 | PREDICTED: uncharacterized protein LOC105218141 [Bactrocera cucurbitae]<>PREDICTED: uncharacterized protein LOC105218141 [Bactrocera cucurbitae]<>PREDICTED: uncharacterized protein LOC105218141 [Bactrocera cucurbitae]<>PREDICTED: uncharacterized protein LOC105218141 [Bactrocera cucurbitae] | 0.0 | - |
| TRINITY_DN15989_c0_g1_i9 | - | 312 | 11 | 0,0353 | GO:0004725 GO:0035335 | PREDICTED: tyrosine-protein phosphatase non-receptor type 9-like isoform X2 [Ceratitis capitata] | 0.0 | - |
| TRINITY_DN17456_c0_g1_i3 | - | 826 | 13 | 0,0157 | GO:0044763 GO:0042623 GO:0031427 GO:0022857 GO:0044765 GO:0000166 GO:0008152 GO:0016020 | PREDICTED: LOW QUALITY PROTEIN: multidrug resistance protein homolog 49 [Bactrocera dorsalis] | 0.0 | - |
| TRINITY_DN17728_c0_g3_i1 | - | 849 | 24 | 0,0283 | - | PREDICTED: sterile alpha and TIR motif-containing protein 1 isoform X9 [Bactrocera cucurbitae] | 0.0 | - |
| TRINITY_DN9887_c0_g1_i2 | - | 4624 | 51 | 0,011 | GO:0016021 GO:0008137 GO:0005743 GO:0006120 GO:0070469 nadh dehydrogenase subunit 2 | PREDICTED: LOW QUALITY PROTEIN: NADH-ubiquinone oxidoreductase chain 2-like [Ceratitis capitata] | 2E-116 | - |
| TRINITY_DN17339_c0_g7_i2 | - | 524 | 11 | 0,021 | GO:0005975 GO:0051287 GO:0004367 GO:0046168 GO:0009331 GO:0042803 GO:0055114 | PREDICTED: glycerol-3-phosphate dehydrogenase [NAD(+)], cytoplasmic-like isoform X3 [Ceratitis capitata] | 5E-71 | - |
| TRINITY_DN10514_c0_g2_i1 | - | 39248 | 1373 | 0,035 | GO:0046872 GO:0016491 GO:0070469 GO:0009055 GO:0016021 GO:0022904 GO:0005739 | cytochrome b [Bactrocera carambolae]<>cytochrome b [Bactrocera carambolae] | 2E-144 | - |
| TRINITY_DN16256_c0_g5_i1 | - | 1171 | 41 | 0,035 | GO:0000146 GO:0005859 GO:0005509 GO:0030016 GO:0003012 GO:0030239 GO:0060361 | PREDICTED: myosin regulatory light chain 2 [Bactrocera dorsalis] | 3E-63 | - |
| TRINITY_DN16851_c0_g3_i1 | - | 1554 | 30 | 0,0193 | - | PREDICTED: putative mediator of RNA polymerase II transcription subunit 26 isoform X2 [Bactrocera cucurbitae] | 3E-85 | - |
| TRINITY_DN12498_c0_g1_i1 | - | 15139 | 431 | 0,0285 | - | PREDICTED: general odorant-binding protein 99b-like [Ceratitis capitata] | 8E-40 | - |
| TRINITY_DN13095_c0_g2_i1 | - | 920 | 11 | 0,012 | - | PREDICTED: uncharacterized protein LOC101455903 [Ceratitis capitata] | 2E-45 | - |
| TRINITY_DN16851_c0_g2_i2 | - | 1552 | 38 | 0,0245 | - | PREDICTED: putative mediator of RNA polymerase II transcription subunit 26 isoform X1 [Bactrocera cucurbitae] | 3E-125 | - |
| TRINITY_DN16851_c0_g2_i1 | - | 857 | 16 | 0,0187 | - | PREDICTED: putative mediator of RNA polymerase II transcription subunit 26 isoform X2 [Bactrocera cucurbitae] | 1E-120 | - |
| TRINITY_DN13095_c0_g1_i1 | - | 1813 | 59 | 0,0325 | - | PREDICTED: uncharacterized protein LOC101455903 [Ceratitis capitata] | 3E-26 | - |
| TRINITY_DN8975_c212_g7_i1 | - | 1086 | 17 | 0,0157 | GO:0042221 GO:0035071 | PREDICTED: general odorant-binding protein 99b-like [Ceratitis capitata] | 1E-24 | - |
| TRINITY_DN12498_c0_g3_i1 | - | 33903 | 240 | 0,0071 | GO:0005549 GO:0007606 GO:0035071 GO:0019236 GO:0005576 GO:0042048 odorant-binding protein 99b | PREDICTED: general odorant-binding protein 99b-like [Ceratitis capitata] | 1E-29 | - |
| TRINITY_DN17005_c0_g1_i1 | - | 1068 | 16 | 0,015 | GO:0016740 GO:0005992 | PREDICTED: alpha,alpha-trehalose-phosphate synthase [UDP-forming] A-like [Ceratitis capitata] | 0.0 | - |
| TRINITY_DN17247_c0_g1_i2 | - | 858 | 13 | 0,0152 | GO:0007527 GO:0004672 GO:0045214 GO:0031430 GO:0006468 GO:0005089 GO:0032321 GO:0005524 | PREDICTED: muscle M-line assembly protein unc-89-like isoform X2 [Ceratitis capitata] | 0.0 | - |
| TRINITY_DN10350_c0_g1_i5 | 11 | - | 394 | 35,82 | GO:0005388 | PREDICTED: calcium-transporting ATPase sarcoplasmic/endoplasmic reticulum type isoform X2 [Bactrocera dorsalis] | 0.0 | - |
| TRINITY_DN17414_c0_g5_i1 | 12 | - | 338 | 28,17 | - | PREDICTED: cytochrome P450 9c1-like [Bactrocera cucurbitae] | 4E-22 | - |
| TRINITY_DN16256_c0_g11_i1 | 17 | - | 293 | 17,24 | GO:0000146 myosin light chain 2 | PREDICTED: myosin regulatory light chain 2 [Bactrocera dorsalis] | 1E-43 | - |
| TRINITY_DN13552_c0_g2_i6 | 30 | - | 636 | 21,20 | - - - | PREDICTED: uncharacterized protein LOC101451629 isoform X12 [Ceratitis capitata] | 7E-70 | - |
| TRINITY_DN12704_c0_g1_i2 | 515 | - | 7463 | 14,49 | GO:0045169 reticulon- isoform a | PREDICTED: reticulon-1-B-like isoform X5 [Ceratitis capitata] | 4E-103 | - |
| TRINITY_DN11233_c0_g1_i1 | 11 | - | 151 | 13,73 | GO:0015991 v-type proton atpase subunit e-like | PREDICTED: V-type proton ATPase subunit e [Bactrocera dorsalis] | 2E-43 | - |
| TRINITY_DN16820_c0_g2_i4 | 10 | - | 168 | 16,80 | GO:0005369 excitatory amino acid transporter 2-like isoform x1 | PREDICTED: excitatory amino acid transporter isoform X1 [Bactrocera cucurbitae] | 0 | - |
| TRINITY_DN9228_c0_g2_i2 | 300 | - | 4648 | 15,49 | GO:1902600 cytochrome c oxidase subunit 6b2-like isoform x2 | PREDICTED: cytochrome c oxidase subunit 6B2-like isoform X2 [Ceratitis capitata] | 7E-43 | - |
| TRINITY_DN17210_c1_g1_i3 | 15 | - | 227 | 15,13 | - - | PREDICTED: adhesive plaque matrix protein isoform X2 [Ceratitis capitata] | 1E-56 | - |
| TRINITY_DN13143_c0_g2_i3 | 20 | - | 344 | 17,20 | GO:0006633 acyl- delta desaturase-like isoform x2 | PREDICTED: acyl-CoA Delta(11) desaturase-like isoform X1 [Ceratitis capitata]<>PREDICTED: acyl-CoA Delta(11) desaturase-like isoform X2 [Ceratitis capitata]<>PREDICTED: acyl-CoA Delta(11) desaturase-like isoform X3 [Ceratitis capitata] | 0 | - |
| TRINITY_DN12195_c0_g1_i1 | 13 | - | 297 | 22,85 | GO:0008152 glutathione s-transferase 1-like | PREDICTED: glutathione S-transferase 1-like [Bactrocera cucurbitae] | 5E-98 | - |
| TRINITY_DN11957_c0_g2_i1 | 14 | - | 264 | 18,86 | GO:0016021 aquaporin -like isoform x2 | PREDICTED: aquaporin AQPAn.G-like isoform X3 [Bactrocera cucurbitae] | 3E-129 | - |
| TRINITY_DN12566_c0_g1_i3 | 16 | - | 241 | 15,06 | GO:0019646 cytochrome oxidase subunit iii | cytochrome c oxidase subunit III (mitochondrion) [Bactrocera cucurbitae]<>cytochrome oxidase subunit III [Bactrocera cucurbitae]<>cytochrome oxidase subunit III [Bactrocera tau]<>cytochrome oxidase subunit III [Bactrocera tau]<>cytochrome c oxidase subunit III [Bactrocera cucurbitae] | 2E-113 | - |
| TRINITY_DN9709_c0_g1_i2 | 10 | - | 205 | 20,50 | - - | PREDICTED: uncharacterized protein LOC105231721 [Bactrocera dorsalis] | 1E-26 | - |
| TRINITY_DN13268_c0_g5_i1 | 140 | - | 2466 | 17,61 | - - - | PREDICTED: sarcocystatin-A-like [Bactrocera dorsalis] | 9E-39 | - |
| TRINITY_DN13343_c0_g1_i1 | 18 | - | 367 | 20,39 | - - - | PREDICTED: muscle-specific protein 20 [Bactrocera dorsalis] | 4E-29 | - |
| TRINITY_DN12629_c0_g1_i2 | 14 | - | 333 | 23,79 | - - - | PREDICTED: uncharacterized protein LOC101453679 isoform X1 [Ceratitis capitata]<>PREDICTED: uncharacterized protein LOC101453679 isoform X2 [Ceratitis capitata] | 4E-63 | - |
| TRINITY_DN11027_c0_g1_i1 | 25 | - | 2211 | 88,44 | - | PREDICTED: uncharacterized protein LOC101461967 [Ceratitis capitata] | 2E-89 | - |
| TRINITY_DN14889_c1_g1_i1 | 233 | - | 5781 | 24,81 | - - - | PREDICTED: uncharacterized protein LOC105234111 isoform X8 [Bactrocera dorsalis] | 3E-88 | gi\|751792922\|ref\|XP_011206125.1\| |
| TRINITY_DN9325_c0_g1_i1 | 21 | - | 554 | 26,38 | GO:0016021 synaptic vesicle glycoprotein 2b | PREDICTED: synaptic vesicle glycoprotein 2B [Bactrocera dorsalis]<>PREDICTED: synaptic vesicle glycoprotein 2B [Bactrocera dorsalis]<>PREDICTED: synaptic vesicle glycoprotein 2B [Bactrocera dorsalis]<>PREDICTED: synaptic vesicle glycoprotein 2B [Bactrocera dorsalis] | 0 | gi\|751477879\|ref\|XP_011194478.1\| |
| TRINITY_DN16287_c0_g1_i1 | 190 | - | 2750 | 14,47 | GO:0004022 alcohol dehydrogenase 2 | PREDICTED: alcohol dehydrogenase 2-like [Ceratitis capitata]<>alcohol dehydrogenase 2 [Ceratitis capitata] | 3E-131 | gi\|751802877\|ref\|XP_011211550.1\| |
| TRINITY_DN14418_c0_g2_i1 | 35 | - | 897 | 25,63 | - - - | PREDICTED: uncharacterized protein LOC105215710 [Bactrocera cucurbitae] | 1E-31 | gi\|807028078\|ref\|XP_012157117.1\| |
| TRINITY_DN14382_c0_g1_i2 | 18 | - | 356 | 19,78 | GO:0006072 glycerol-3-phosphate mitochondrial isoform x2 | PREDICTED: glycerol-3-phosphate dehydrogenase, mitochondrial isoform X1 [Bactrocera cucurbitae] | 0 | gi\|499014541\|ref\|XP_004537808.1\| |
| TRINITY_DN14889_c1_g1_i3 | 25 | - | 366 | 14,64 | - - | PREDICTED: uncharacterized protein LOC105234111 isoform X6 [Bactrocera dorsalis] | 4E-158 | gi\|751796554\|ref\|XP_011208097.1\| |
| TRINITY_DN14419_c0_g1_i1 | 11 | - | 158 | 14,36 | GO:0005762 39s ribosomal protein mitochondrial-like | PREDICTED: 39S ribosomal protein L14, mitochondrial-like [Ceratitis capitata] | 1E-69 | gi\|751463717\|ref\|XP_011186772.1\| |
| TRINITY_DN17073_c0_g1_i2 | 11 | - | 238 | 21,64 | GO:0003779 low quality protein: gelsolin-like | PREDICTED: gelsolin-like [Ceratitis capitata] | 0 | gi\|807033353\|ref\|XP_012158865.1\| |
| TRINITY_DN16698_c0_g2_i2 | 16 | - | 360 | 22,50 | GO:0005737 isoform a | PREDICTED: alpha-N-acetylgalactosaminidase [Bactrocera cucurbitae]<>PREDICTED: alpha-N-acetylgalactosaminidase [Bactrocera cucurbitae] | 0 | gi\|807035293\|ref\|XP_012159452.1\| |
| TRINITY_DN13718_c0_g1_i2 | 10 | - | 209 | 20,90 | GO:0005975 maltase a3 | PREDICTED: maltase A3 [Bactrocera dorsalis] | 0 | gi\|499000121\|ref\|XP_004534268.1\| |
| TRINITY_DN10350_c0_g1_i3 | 47 | - | 742 | 15,79 | GO:0005388 calcium-transporting atpase sarcoplasmic endoplasmic reticulum type isoform x1 | PREDICTED: calcium-transporting ATPase sarcoplasmic/endoplasmic reticulum type isoform X2 [Bactrocera dorsalis] | 0 | gi\|751455378\|ref\|XP_011182210.1\| |
| TRINITY_DN14062_c0_g1_i13 | 14 | - | 513 | 36,64 | GO:0046933 | PREDICTED: ATP synthase subunit beta, mitochondrial-like [Ceratitis capitata] | 0.0 | gi\|751470713\|ref\|XP_011190598.1\| |
| TRINITY_DN16568_c0_g1_i8 | 10 | - | 159 | 15,90 | GO:0005991 trehalase isoform x1 | PREDICTED: trehalase-like isoform X1 [Ceratitis capitata]<>PREDICTED: trehalase-like isoform X2 [Ceratitis capitata]<>PREDICTED: trehalase-like isoform X3 [Ceratitis capitata]<>PREDICTED: trehalase-like isoform X4 [Ceratitis capitata]<>PREDICTED: trehalase-like isoform X5 [Ceratitis capitata] | 0 | gi\|351000076\|ref\|YP_004891170.1\| |
| TRINITY_DN17502_c0_g2_i1 | 10 | - | 293 | 29,30 | GO:0004177 membrane alanyl aminopeptidase-like | PREDICTED: membrane alanyl aminopeptidase [Bactrocera cucurbitae] | 0.0 | gi\|751802702\|ref\|XP_011211454.1\| |
| TRINITY_DN13343_c0_g2_i1 | 23 | - | 484 | 21,04 | - - - | PREDICTED: muscle-specific protein 20 [Bactrocera cucurbitae] | 4E-82 | gi\|751786655\|ref\|XP_011202717.1\| |
| TRINITY_DN14062_c0_g1_i9 | 11 | - | 253 | 23,00 | GO:0046933 atp synthase subunit mitochondrial-like | PREDICTED: ATP synthase subunit beta, mitochondrial-like [Ceratitis capitata] | 0 | gi\|751780522\|ref\|XP_011199387.1\| |
| TRINITY_DN13560_c0_g1_i8 | 59 | - | 2081 | 35,27 | - | PREDICTED: uncharacterized protein LOC105213980 isoform X3 [Bactrocera cucurbitae] | 3E-43 | gi\|498942145\|ref\|XP_004521668.1\| |
| TRINITY_DN17728_c0_g3_i1 | 32 | - | 506 | 15,81 | - - | PREDICTED: sterile alpha and TIR motif-containing protein 1 isoform X9 [Bactrocera cucurbitae] | 0 | gi\|498950236\|ref\|XP_004522983.1\| |
| TRINITY_DN10514_c0_g2_i1 | 14 | - | 273 | 19,50 | GO:0046872 cytochrome b | cytochrome b [Bactrocera carambolae]<>cytochrome b [Bactrocera carambolae] | 2E-144 | gi\|751776013\|ref\|XP_011214683.1\| |
| TRINITY_DN16256_c0_g8_i1 | 12 | - | 174 | 14,50 | GO:0000146 myosin light chain 2 | PREDICTED: myosin regulatory light chain 2 [Bactrocera dorsalis] | 3E-63 | gi\|751787646\|ref\|XP_011203247.1\| |
| TRINITY_DN15528_c0_g1_i1 | 11 | - | 288 | 26,18 | GO:0004601 isoform b | PREDICTED: peroxidase [Bactrocera dorsalis] | 0 | gi\|499007470\|ref\|XP_004536080.1\| |
| TRINITY_DN14990_c1_g1_i17 | 14 | - | 318 | 22,71 | GO:0070865 tropomyosin isoform l | PREDICTED: calponin homology domain-containing protein DDB_G0272472-like isoform X19 [Ceratitis capitata] | 1E-112 | gi\|751466092\|ref\|XP_011188063.1\| |
| TRINITY_DN17007_c0_g1_i3 | 44 | - | 604 | 13,73 | - - | PREDICTED: alpha-tocopherol transfer protein-like [Ceratitis capitata] | 5E-91 | gi\|751475518\|ref\|XP_011193202.1\| |
| TRINITY_DN14948_c0_g16_i1 | 118 | - | 14405 | 122,08 | GO:0005576 | yolk polypeptide, partial [Anastrepha suspensa] | 2E-105 | gi\|751776009\|ref\|XP_011214681.1\| |
| TRINITY_DN16851_c0_g3_i1 | 14 | - | 321 | 22,93 | - -- | PREDICTED: putative mediator of RNA polymerase II transcription subunit 26 isoform X2 [Bactrocera cucurbitae] | 3E-85 | gi\|498962696\|ref\|XP_004525055.1\| |
| TRINITY_DN13268_c0_g2_i1 | 22 | - | 1314 | 59,73 | - | PREDICTED: sarcocystatin-A-like [Bactrocera dorsalis] | 9E-23 | gi\|498997392\|ref\|XP_004533591.1\| |
| TRINITY_DN13095_c0_g2_i1 | 17 | - | 375 | 22,06 | - -- | PREDICTED: uncharacterized protein LOC101455903 [Ceratitis capitata] | 2E-45 | gi\|751454129\|ref\|XP_011181521.1\| |
| TRINITY_DN16851_c0_g2_i2 | 351 | - | 5225 | 14,89 | - - | PREDICTED: putative mediator of RNA polymerase II transcription subunit 26 isoform X1 [Bactrocera cucurbitae] | 3E-125 | gi\|751792604\|ref\|XP_011205955.1\| |
| TRINITY_DN16851_c0_g2_i1 | 14 | - | 237 | 16,93 | - - | PREDICTED: putative mediator of RNA polymerase II transcription subunit 26 isoform X2 [Bactrocera cucurbitae] | 1E-120 | gi\|751792922\|ref\|XP_011206125.1\| |
| TRINITY_DN17005_c0_g1_i1 | 11 | - | 205 | 18,64 | GO:0016740 trehalose-phosphate synthase | PREDICTED: alpha,alpha-trehalose-phosphate synthase [UDP-forming] A-like [Ceratitis capitata] | 0 | gi\|498936802\|ref\|XP_004520492.1\| |
| TRINITY_DN13610_c0_g2_i7 | 13202 | - | 17 | 0,0013 | - | Cell wall-associated hydrolase [Trichuris trichiura] | 2E-60 | gi\|498925032\|ref\|XP_004517575.1\| |
| TRINITY_DN9842_c0_g1_i2 | 293 | - | 11 | 0,0375 | GO:0006413 protein translation factor sui1 homolog | PREDICTED: protein translation factor SUI1 homolog isoform X1 [Ceratitis capitata]<>PREDICTED: protein translation factor SUI1 homolog [Bactrocera cucurbitae]<>PREDICTED: protein translation factor SUI1 homolog [Bactrocera dorsalis] | 8E-51 | gi\|751469073\|ref\|XP_011189700.1\| |
| TRINITY_DN17060_c1_g4_i2 | 2729 | - | 52 | 0,0191 | - - - | PREDICTED: uncharacterized protein LOC101451335 [Ceratitis capitata] | 4E-15 | gi\|751464112\|ref\|XP_011186987.1\| |
| TRINITY_DN10186_c0_g1_i4 | 300 | - | 23 | 0,0767 | GO:0005875 heat shock protein 27 | PREDICTED: heat shock protein 27 [Bactrocera dorsalis] | 6E-83 | gi\|498936802\|ref\|XP_004520492.1\| |
| TRINITY_DN15356_c0_g1_i2 | 498 | - | 11 | 0,0221 | GO:0045454 thioredoxin domain-containing protein 9 | PREDICTED: thioredoxin domain-containing protein 9 [Bactrocera dorsalis] | 5E-97 | gi\|751461235\|ref\|XP_011185415.1\| |
| TRINITY_DN12145_c0_g1_i3 | 7214 | - | 11 | 0,0015 | - | unnamed protein product [Schistosoma mansoni] | 2E-17 | gi\|751442834\|ref\|XP_011196081.1\| |
| TRINITY_DN11806_c0_g1_i1 | 1123 | - | 42 | 0,0374 | - - | PREDICTED: uncharacterized protein LOC105212305 [Bactrocera cucurbitae] | 8E-36 | gi\|156765993\|ref\|YP_001434551.1\| |
| TRINITY_DN15227_c0_g1_i4 | 978 | - | 48 | 0,0491 | GO:0003677 transcription factor adf-1-like isoform x2 | PREDICTED: transcription factor Adf-1-like isoform X1 [Ceratitis capitata] | 2E-123 | gi\|751802877\|ref\|XP_011211550.1\| |
| TRINITY_DN17307_c1_g1_i1 | 768 | - | 13 | 0,0169 | GO:0046961 atp synthase subunit mitochondrial-like isoform x2 | PREDICTED: ATP synthase subunit epsilon, mitochondrial-like isoform X2 [Ceratitis capitata] | 4E-21 | gi\|751770384\|ref\|XP_011199103.1\| |
| TRINITY_DN14569_c0_g1_i1 | 239 | - | 12 | 0,0502 | GO:0005080 guanine nucleotide-binding protein subunit beta-like protein | PREDICTED: guanine nucleotide-binding protein subunit beta-like protein [Bactrocera cucurbitae]<>PREDICTED: guanine nucleotide-binding protein subunit beta-like protein [Bactrocera dorsalis] | 3E-123 | gi\|498934011\|ref\|XP_004519784.1\| |
| TRINITY_DN16719_c0_g3_i3 | 377 | - | 28 | 0,0743 | GO:0030307 isoform b | PREDICTED: egl nine homolog 1 isoform X2 [Bactrocera cucurbitae] | 3E-116 | gi\|498962681\|ref\|XP_004525052.1\| |
| TRINITY_DN15804_c0_g2_i1 | 1197 | - | 40 | 0,0334 | - -- | PREDICTED: protein spitz [Bactrocera dorsalis] | 7E-69 | gi\|2852127\|gb\|AAC01961.1\| |
| TRINITY_DN10357_c0_g1_i1 | 320 | - | 10 | 0,0313 | GO:0000786 histone h2a | PREDICTED: histone H2A-like [Linepithema humile] | 1E-52 | gi\|751456424\|ref\|XP_011182780.1\| |
| TRINITY_DN13275_c0_g3_i3 | 158 | - | 10 | 0,0633 | - - | PREDICTED: baculoviral IAP repeat-containing protein 5.2-like [Ceratitis capitata] | 9E-65 | gi\|751786655\|ref\|XP_011202717.1\| |
| TRINITY_DN9911_c0_g1_i3 | 261 | - | 11 | 0,0421 | GO:0022008 nascent polypeptide-associated complex subunit alpha | PREDICTED: nascent polypeptide-associated complex subunit alpha-like isoform X1 [Ceratitis capitata]<>PREDICTED: nascent polypeptide-associated complex subunit alpha-like isoform X2 [Ceratitis capitata]<>PREDICTED: nascent polypeptide-associated complex subunit alpha-like isoform X3 [Ceratitis capitata] | 1E-60 | gi\|498942252\|ref\|XP_004521685.1\| |
| TRINITY_DN15856_c0_g3_i1 | 132 | - | 10 | 0,0758 | GO:0007049 transcription factor dp | PREDICTED: transcription factor Dp [Bactrocera cucurbitae]<>PREDICTED: transcription factor Dp [Bactrocera cucurbitae]<>PREDICTED: transcription factor Dp [Bactrocera cucurbitae]<>PREDICTED: transcription factor Dp [Bactrocera cucurbitae] | 2E-177 | gi\|751456422\|ref\|XP_011182778.1\| |
| TRINITY_DN14543_c0_g1_i2 | 617 | - | 10 | 0,0162 | GO:0000786 histone 1 | GK18811 [Drosophila willistoni]<>GK18811 [Drosophila willistoni] | 2E-30 | gi\|751456424\|ref\|XP_011182780.1\| |
| TRINITY_DN10960_c0_g2_i2 | 850 | - | 34 | 0,04 | GO:0000022 ribosomal protein s18 | FI09342p [Drosophila melanogaster] | 2E-71 | gi\|499007987\|ref\|XP_004536206.1\| |
| TRINITY_DN17494_c0_g1_i3 | 549 | - | 32 | 0,0583 | - -- | PREDICTED: mucin-5AC isoform X1 [Bactrocera dorsalis]<>PREDICTED: mucin-5AC isoform X1 [Bactrocera dorsalis]<>PREDICTED: mucin-5AC isoform X1 [Bactrocera dorsalis]<>PREDICTED: mucin-5AC isoform X1 [Bactrocera dorsalis]<>PREDICTED: mucin-5AC isoform X1 [Bactrocera dorsalis] | 3E-179 | gi\|669215019\|emb\|CDW61002.1\| |
| TRINITY_DN15741_c0_g1_i3 | 291 | - | 23 | 0,079 | - - - | PREDICTED: protein daughter of sevenless [Bactrocera dorsalis] | 0 | gi\|498934530\|ref\|XP_004519899.1\| |
| TRINITY_DN15627_c0_g1_i2 | 341 | - | 14 | 0,0411 | GO:0006909 nimrod c4 | PREDICTED: delta-like protein A [Bactrocera dorsalis] | 7E-143 | gi\|498981536\|ref\|XP_004529632.1\| |
| TRINITY_DN13678_c0_g1_i1 | 20264 | - | 30 | 0,0015 | GO:0005524 heat shock protein 70 | heat shock protein 70 [Bactrocera correcta] | 0.0 | gi\|751778168\|ref\|XP_011198116.1\| |
| TRINITY_DN15745_c0_g1_i2 | 337 | - | 27 | 0,0801 | - - - | PREDICTED: probable phosphorylase b kinase regulatory subunit alpha isoform X6 [Bactrocera cucurbitae] | 0 | gi\|751786534\|ref\|XP_011202652.1\| |
| TRINITY_DN14198_c3_g11_i1 | 512 | - | 38 | 0,0742 | - - - | PREDICTED: heat shock protein 23-like [Bactrocera cucurbitae] | 4E-69 | gi\|350646623\|emb\|CCD58743.1\| |
| TRINITY_DN16644_c0_g1_i7 | 171 | - | 14 | 0,0819 | - - - | PREDICTED: uncharacterized protein LOC105220311 [Bactrocera cucurbitae] | 8E-11 | gi\|751439118\|ref\|XP_011182502.1\| |
| TRINITY_DN14656_c0_g1_i2 | 162 | - | 11 | 0,0679 | - -- | PREDICTED: transcription factor Adf-1-like isoform X2 [Ceratitis capitata] | 2E-59 | gi\|498948366\|ref\|XP_004522684.1\| |
| TRINITY_DN16414_c0_g1_i1 | 31892 | - | 43 | 0,0013 | GO:0005524 heat shock protein 70 | heat shock protein 70 [Rhagoletis pomonella] | 0.0 | gi\|498938775\|ref\|XP_004521016.1\| |
| TRINITY_DN16414_c0_g1_i2 | 3436 | - | 10 | 0,0029 | GO:0005524 heat shock protein 70 | heat shock protein 70 [Rhagoletis pomonella] | 0.0 | gi\|751451998\|ref\|XP_011180350.1\| |
| TRINITY_DN16226_c0_g2_i2 | 5934 | - | 333 | 0,0561 | GO:0007411 tubulin beta-3 chain | PREDICTED: tubulin beta-3 chain-like [Ceratitis capitata]<>PREDICTED: tubulin beta-3 chain [Bactrocera cucurbitae]<>PREDICTED: tubulin beta-3 chain [Bactrocera dorsalis] | 7E-178 | gi\|751458710\|ref\|XP_011184036.1\| |
| TRINITY_DN16480_c0_g3_i3 | 5506 | - | 95 | 0,0173 | GO:0005158 downstream of kinase | PREDICTED: uncharacterized protein LOC101450146 [Ceratitis capitata] | 0.0 | gi\|751804208\|ref\|XP_011212275.1\| |
| TRINITY_DN17415_c0_g1_i5 | 1641 | - | 23 | 0,014 | GO:0006200 dna topoisomerase 2 isoform x2 | PREDICTED: DNA topoisomerase 2 isoform X2 [Ceratitis capitata] | 0.0 | gi\|815814502\|ref\|XP_012228276.1\| |
| TRINITY_DN13101_c0_g3_i2 | 2344 | - | 99 | 0,0422 | - -- | PREDICTED: uncharacterized protein LOC105210637 [Bactrocera cucurbitae] | 3E-17 | gi\|498967166\|ref\|XP_004525995.1\| |
| TRINITY_DN17639_c1_g2_i9 | 305 | - | 24 | 0,0787 | GO:0006468 serine threonine-protein kinase greatwall | PREDICTED: serine/threonine-protein kinase greatwall isoform X1 [Bactrocera dorsalis] | 0 | gi\|499006474\|ref\|XP_004535833.1\| |
| TRINITY_DN14198_c1_g1_i2 | 532 | - | 26 | 0,0489 | - -- | PREDICTED: heat shock protein 23-like [Bactrocera dorsalis] | 7E-62 | gi\|751458995\|ref\|XP_011184190.1\| |
| TRINITY_DN15631_c0_g1_i1 | 2625 | - | 61 | 0,0232 | GO:0007411 tubulin beta-3 chain | GK22203 [Drosophila willistoni]<>GK22203 [Drosophila willistoni] | 4E-93 | gi\|195466282\|ref\|XP_002075981.1\| |
| TRINITY_DN15804_c0_g1_i1 | 651 | - | 21 | 0,0323 | - - - | PREDICTED: protein spitz [Bactrocera cucurbitae]<>PREDICTED: protein spitz [Bactrocera cucurbitae] | 3E-34 | gi\|211938661\|gb\|ACJ13227.1\| |
| TRINITY_DN10865_c0_g29_i1 | 568 | - | 10 | 0,0176 | GO:0000022 probable 60s ribosomal protein l37-a | PREDICTED: probable 60S ribosomal protein L37-A [Bactrocera dorsalis] | 2E-29 | gi\|751786435\|ref\|XP_011202598.1\| |
