# Supplementary figures and images for: Transcriptome analysis of Anastrepha fraterculus sp. 1 males, females, and embryos: insights into development, courtship, and reproduction
Source: BMC Genet. 2020 Dec 18;21(Suppl 2):136. doi: 10.1186/s12863-020-00943-2 (PMC7747455; doi:10.1186/s12863-020-00943-2)

### Males vs. females

$r = 0,87$ ;  $P < 0,0001$

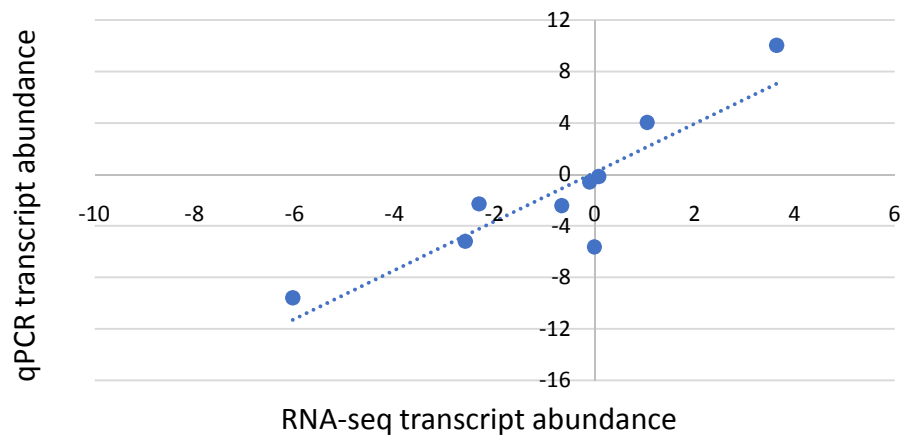

### Embryos vs. males

$r = 0,93$ ;  $P < 0,0001$

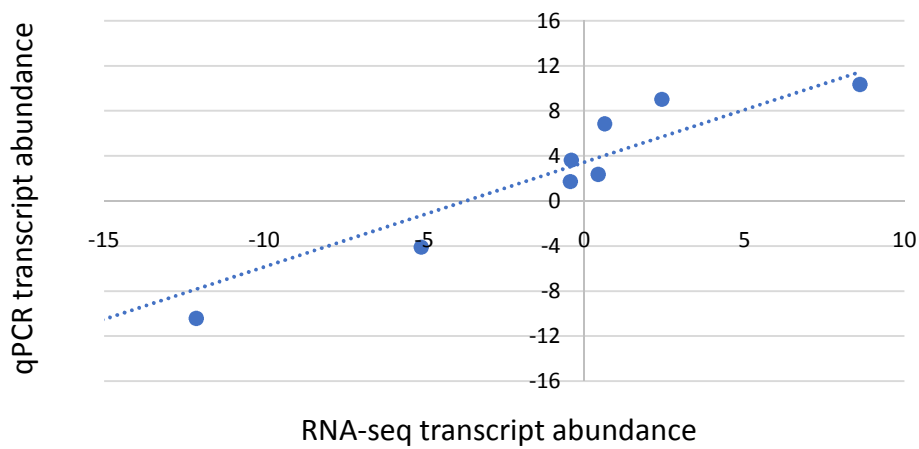

### Embryos vs. females

$r = 0,87$ ;  $P < 0,0001$

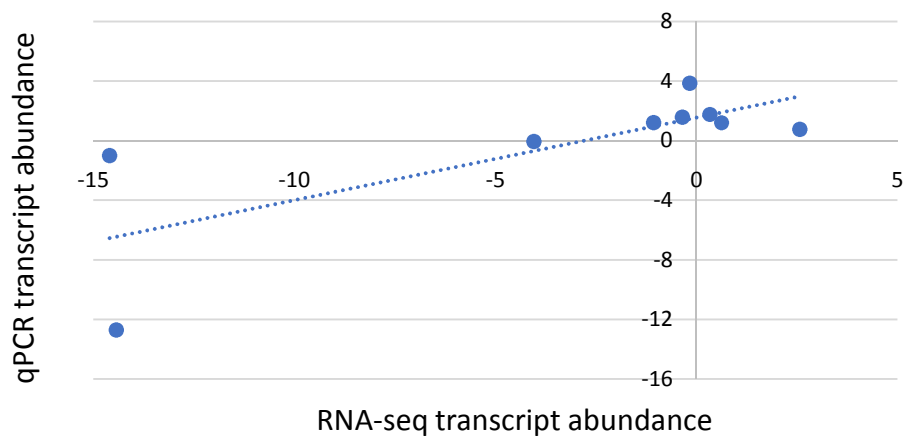

Supplement: Supplementary file 8 — Additional file 8. Correlation between RNA-Seq and qPCR datasets for the nine analyzed transcripts (Table 3) in 72 h embryos, virgin males and virgin females of A. fraterculus. For each comparison, transcripts log2 FC from RNA-Seq analysis are plotted against the mean NRQs log2 fold change from qPCR analysis. Spearman’s rank correlation coefficients (r) for each comparison are shown. For the three comparisons, r values were highly significant (P < 0.0001) and demonstrate a high degree of correlation between the two datasets. Data used to create these graphs are detailed in Additional File 7 (RNA-Seq data) and Fig. 4 (qPCR data). [file 12863_2020_943_MOESM8_ESM.pdf]
